# Single-Cell Metabolic Profiling in a Glioblastoma Coculture Model Using AP-MALDI-Based Mass Spectrometry Imaging

**DOI:** 10.1021/acs.analchem.5c07924

**Published:** 2026-04-07

**Authors:** Une Kontrimaite, Kei F. Carver Wong, Sandra Martínez-Jarquín, Phoebe McCrorie, Ruman Rahman, Dong-Hyun Kim

**Affiliations:** † Biodiscovery Institute, School of Medicine, 6123 University of Nottingham, Nottingham, NG7 2RD U.K.; ‡ Centre for Analytical Bioscience, Advanced Materials & Health Technologies Division, School of Pharmacy, University of Nottingham, Nottingham, NG7 2RD U.K.; § College of Pharmacy, Kyungpook National University, Daegu 41566, Republic of Korea

## Abstract

Mass spectrometry imaging enables spatially resolved, label-free detection of metabolites in tissue and culture systems, providing insight into their metabolic landscapes and spatial distribution. However, conventional approaches often lack the spatial resolution and specificity needed to investigate metabolic heterogeneity at the single-cell level, particularly in physiologically relevant models. Here, we present a single-cell ambient mass spectrometry imaging platform, enabling direct chemical mapping of metabolites at a 10 μm resolution. This method integrates cell labeling, high-resolution microscopy, and AP-MALDI Orbitrap mass spectrometry imaging to achieve cell-type-specific metabolite profiling. To demonstrate its application, we applied this approach to glioblastoma (GBM), an aggressive adult brain tumor characterized by cellular heterogeneity, metabolic adaptation, and infiltrative growth within the tumor microenvironment. A coculture model combining patient-derived glioblastoma invasive margin cells with human cortical astrocytes was used to recapitulate the invasive niche. Distinct metabolic signatures emerged upon glioblastoma–astrocyte interaction, involving pathways related to nucleotide metabolism, phospholipid turnover, and tyrosine metabolism. These findings suggest cell-type-specific metabolic activity and a potential intercellular metabolic interplay. Overall, this workflow offers a broadly accessible and robust approach for investigating metabolic heterogeneity at cellular resolution, enabling insights into metabolic interactions of heterogeneous cell types in both disease and nondisease settings.

## Graphical abstract

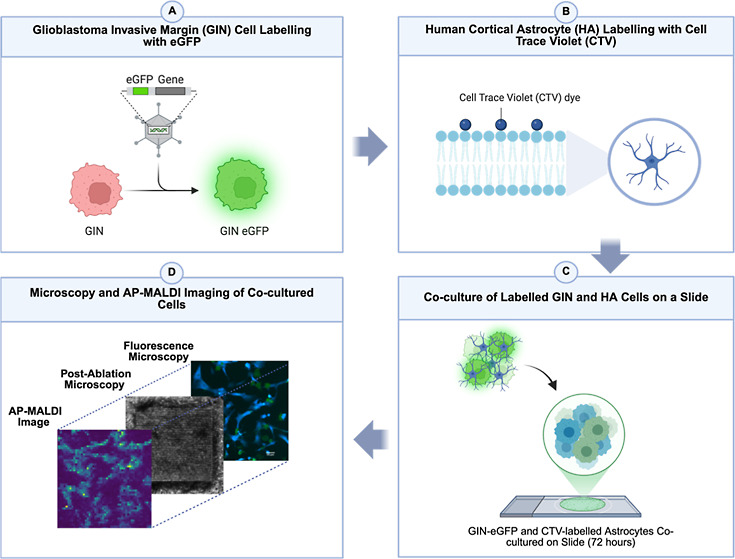

## Introduction

Bulk metabolomics has enabled insights into global metabolic trends by analyzing populations of thousands to millions of cells simultaneously.^1^ Such approaches often mask subtle but biologically important heterogeneity within and between individual cells.^2^ To overcome this limitation, a variety of single-cell metabolomics techniques have been developed to investigate cellular metabolism at the single-cell level, including secondary ion mass spectrometry (SIMS), matrix-assisted laser desorption/ionization mass spectrometry, and electrospray ionization MS (ESI–MS)-based approaches such as DESI, nano-DESI, and LESA.^2–5^ Among these, imaging mass spectrometry methods such as SIMS and MALDI are particularly valuable, as they provide label-free metabolite mapping.^6^ SIMS offers exceptional spatial resolution and sensitivity for small molecules, but extensive fragmentation can limit detection of larger metabolites and lipids.^7^ MALDI provides a softer ionization that enables the detection of a broad range of biomolecules, including metabolites, lipids, and peptides, making it particularly well-suited for single-cell metabolic analysis. Unlike vacuum MALDI, AP-MALDI-MS operates under ambient conditions, reducing sample preparation constraints and better preserving volatile metabolites such as short-chain fatty acids, volatile organic acids, and small polar compounds that may be lost under vacuum conditions. Although AP-MALDI-MS may generate lower ion signals than vacuum MALDI, coupling with a high-resolution Orbitrap mass analyzer provides high mass accuracy and resolving power, enabling discrimination of closely spaced and isobaric metabolites.^8–10^ This capability facilitates more confident metabolite annotation and supports spatially resolved metabolomic analyses.^3,11,12^

Recent innovations in spatial metabolite imaging have advanced the ability to visualize metabolic activity with cellular precision. Workflows such as SpaceM, HT SpaceM, and the approach developed by Zhang et al. have demonstrated the feasibility of imaging metabolites at single-cell resolution.^13–15^ Despite these advances, a critical limitation remains: most existing approaches lack the ability to precisely identify individual cell types within heterogeneous samples. This constraint limits their application to coculture and tissue systems where multiple, metabolically distinct cell populations interact. Bridging this gap by integrating spatially resolved metabolite mapping with robust cell-type identification would enable direct investigation of intercellular metabolic communication within complex microenvironments, such as tumor niches composed of cancer cells and diverse supportive stromal cells.

To address the unmet need, we developed a microscopy-guided single-cell AP-MALDI-mass spectrometry imaging (AP-MALDI-MSI) workflow, integrated with a high-resolution Orbitrap mass analyzer, to enable spatially resolved metabolomic profiling at the single-cell level. Given the highly invasive nature of glioblastoma and its interactions with surrounding astrocytes that contribute to recurrence, delineating metabolic crosstalk at the single-cell level is essential to uncovering potential vulnerabilities. To retain information regarding cell identity within mixed populations, we incorporated fluorescence cell-labeling for human cortical astrocytes and patient-derived cancer cells. This approach supported the analysis of complex coculture systems while maintaining single-cell specificity and spatial context. To our knowledge, this represents the first demonstration of glioblastoma–astrocyte metabolic crosstalk captured at single-cell resolution using AP-MALDI-MSI, a versatile framework for interrogating heterogeneous intercellular metabolic interactions in disease and nondisease states.

## Experimental Section

### Cell Culture

Patient-derived GBM cells used for single-cell mass spectrometry analysis were isolated from the 5-aminolevulinic acid (5-ALA) fluorescence-positive invasive margin of human GBM biopsy tissue during fluorescence-guided neurosurgery.^16^ These GBM invasive margin cells, termed GIN 8, GIN 28, and GIN 31, correspond to individual patients. All GIN cell lines were cultured in Dulbecco’s Modified Eagle’s Medium (Sigma-Aldrich, St. Louis, MO, USA) supplemented with 10% fetal bovine serum, 1 g/L glucose, and 2 mM *l* -glutamine (Sigma-Aldrich). Human cortical astrocytes (human astrocytes (HA)) (ScienCell Research Laboratories, Cat. No. 1800) were cultured in Astrocyte Medium (ScienCell, Cat. No. 1801) following the manufacturer’s instructions. Both GBM and astrocyte cultures were maintained at 37 °C in a humidified atmosphere with 5% CO_2_ and 90% relative humidity. For coculture experiments, both cell types were maintained in Astrocyte Medium (ScienCell, Cat. No. 1801) to support optimal astrocyte and cancer cell viability.

GIN cells were genetically labeled with eGFP using a lentiviral construct expressing eGFP under the control of the EF1-α promoter. Viral particles were introduced at a multiplicity of infection of 50–100, depending on the cell line, with Polybrene being used to enhance transduction efficiency. Human cortical astrocytes were labeled with CellTrace Violet Cell Proliferation Kit (Invitrogen, Thermo Fisher Scientific, Cat. No. C34557) at a dilution of 2 μL per 5 mL of phosphate-buffered saline (PBS), following the manufacturer’s protocol.

### Sample Preparation

Cells were cultured in 8-well μ-slides with removable chambers (Ibidi, cat. no. 80826), precoated with poly- *l* -lysine (PLL; ScienCell, cat. no. 0413) at a concentration of 15 μg/mL to enhance cell adhesion. For monoculture conditions, 10,000 cells were seeded per well. In coculture conditions designed to mimic the GBM invasive margin, 3000 GIN cells and 7000 HA cells were seeded onto each well. On day 5 of culture, cells were washed with PBS, fixed with 4% PFA for 15 min, and subsequently rinsed with PBS followed by sterile water. The slide was then desiccated for 15 min to remove residual moisture prior to analysis. The chosen seeding ratio (30% GIN to 70% HA) reflects proportions reported in human GBM tissue at the invasive margin.^17,18^

### Pre- and Postimaging Microscopy

Microscopy was performed on the same day as AP-MALDI-MSI analysis using a Nikon Eclipse Ti inverted microscope equipped with a Plan Fluor 10×/0.30 NA objective. Brightfield and fluorescence images were acquired using standard acquisition software. eGFP fluorescence (Ex ∼488 nm and Em ∼509 nm) and CellTrace Violet (CTV; Ex ∼405 nm and Em ∼450 nm) were used to visualize GBM and astrocyte populations, respectively. Postablation images were acquired after AP-MALDI-MSI to visualize laser ablation marks, which were then used for image alignment and overlay to aid in single-cell regions of interest (ROI) identification. Imaging parameters were kept consistent across all samples, and analyses were complemented using ImageJ software.

### Matrix Application

Matrix application was performed using a SunCollect automated pneumatic sprayer (SunChrom, Friedrichsdorf, Germany) to uniformly coat the surface of fixed GBM and astrocyte cell samples. For matrix optimization, three matrices were investigated: 2,5-dihydroxybenzoic acid (DHB) prepared at 7.5 mg/mL in 70:30 (v/v) acetonitrile/water (ACN/H_2_O), α-cyano-4-hydroxycinnamic acid (CHCA) at 5 mg/mL in 70:30 (v/v) ACN/H_2_O, and 5 mg/mL 9-aminoacridine (9AA) dissolved in 70:30 (v/v) methanol (MeOH)/water. Matrices were applied in 18 layers, beginning with a flow rate of 20 μL/min for the first layer, followed by 40 μL/min for the second layer, and 60 μL/min for all subsequent layers. The nozzle-to-sample distance was maintained at 20 mm, with a spraying velocity of 800 mm/min and a drying time of 2 s between each layer to ensure uniform crystallization and matrix coverage.

### AP-MALDI-MSI and Data Processing

Samples were analyzed using an AP-MALDI UHR (MassTech Inc., Columbia, MD, USA) ion source coupled to a Q Exactive Orbitrap mass spectrometer (Thermo Fisher Scientific, Hemel Hempstead, UK). Instrument parameters for the AP-MALDI source were configured via Target-ng software (MassTech Inc.). The source was operated in the raster mode with a laser spot size of 10 μm, the laser energy set to 4.5%, and a laser repetition rate of 2.5 kHz. The ion transfer capillary temperature was maintained at 400 °C, and the spray voltage was set to 2.5 kV.

Mass spectrometric acquisition was performed in both positive and negative ion modes over an *m*/*z* range of 75–1050. The mass resolving power was set to 140,000 at *m*/*z* 200. The automatic gain control target was 4 × 10^6^, with a maximum injection time of 400 ms and a scan rate of 1 scan/s.

Mass spectrometry imaging data was acquired using an AP-MALDI system (MassTech Inc., Columbia, MD, USA), and mass spectral data were collected using Xcalibur software (Thermo Fisher Scientific). Raw data files were converted to the imzML and ibd formats using ProteoWizard and the MT imzML Converter (MassTech Inc.), enabling compatibility with downstream analysis platforms.^19^

Preprocessing and normalization were performed using the *Cardinal* package in R.^20^ Total ion current (TIC) normalization was applied to correct for overall intensity variation. Baseline correction was carried out by interpolating a baseline from local minima in the spectra.^21^ Peak picking was performed by estimating a signal-to-noise ratio (SNR) of 5, calculated from the mean absolute deviation of wavelet-convolved spectra. Peaks were then aligned across spectra using a 5 ppm of *m*/*z* tolerance.

MS images were first generated as ion intensity maps for selected *m*/*z* values in the *Cardinal* package in R. These were then aligned with pre-MS fluorescence microscopy images and postablation brightfield images to identify ROI corresponding to single cells. Ion intensity values were subsequently extracted from these ROIs for downstream statistical analysis.

### Single-Cell Region of Interest Selection

Pre- and post-AP-MALDI-MSI microscopy images were first used to identify the overall location of the ablated region by visualizing laser ablation marks and their correspondence to the sample area (Figure S2). This provided an initial, coarse spatial reference for alignment between microscopy and MSI data.

Subsequently, higher-magnification microscopy images were used to more precisely identify and delineate individual cells within these regions. Cell selection was based on morphological characteristics, including cell shape, size, and boundary clarity, to ensure an accurate identification of single cells. To ensure the analysis of true single-cell profiles, regions where cells were overlapping, clustered, or not clearly distinguishable were excluded. Only well-isolated cells with clearly defined boundaries were included in the final analysis.

To preserve the native metabolic state of the cells, no nuclear or fluorescent staining was used prior to MSI. This approach minimized postculture processing steps and reduced the risk of extracellular metabolite loss associated with additional washing procedures.

### Metabolite Identification

Metabolite and lipid identification was conducted using a molecular formula prediction (MFP) approach implemented in a tool developed by the School of Pharmacy, University of Nottingham.^22^ Mass spectral features acquired in both positive and negative ion modes were matched to candidate molecular formulas based on accurate mass (±5 ppm), with elemental composition and double bond equivalence criteria applied.

Candidate formulas were subsequently screened against the Human Metabolome Database (HMDB) and LIPID MAPS to assign putative metabolite and lipid identities. Annotation was restricted to chemically plausible compositions and compounds consistent with the experimental context. Given the absence of chromatographic separation, metabolite annotations were primarily based on accurate mass and molecular formula predictions and were therefore considered putative (level 3 identification). Where available, selected metabolites were further supported by comparison of MS/MS fragmentation spectra acquired from LC–MS analyses of coculture experiments under comparable experimental conditions, corresponding to level 2 identification confidence.

Following annotation, a biological relevance selection step was applied to prioritize metabolites for downstream analysis. Features were retained if they corresponded to known endogenous mammalian metabolites and were associated with metabolic pathways relevant to cancer biology, including amino acid metabolism, nucleotide metabolism, central carbon metabolism, lipid metabolism, and redox processes.^23,24^ Annotation plausibility was assessed using HMDB and KEGG database information alongside literature reports on cancer and glioblastoma metabolism. Features considered unlikely to represent biologically meaningful endogenous metabolites, such as environmental contaminants, sample preparation-derived species, or low-mass heterocyclic features potentially arising from in-source fragmentation or ambiguous formula matching, were excluded from further analysis.

### AP MALDI Data Analysis

AP-MALDI-MSI data were generated across three experimental data sets: (i) matrix optimization using HA cells (with DHB, CHCA, and 9AA matrices), (ii) monoculture experiments comparing GIN (GIN 8, GIN 28, and GIN 31) and HA cells, and (iii) coculture experiments modeling GBM–astrocyte interactions.

#### Matrix Optimization Analysis

Intermatrix comparisons were performed using annotated metabolites to evaluate the number and overlap of detected features across matrices, visualized using Venn diagrams. Metabolites were further grouped into major biochemical classes, including nucleotides and nucleobases, amino acids and derivatives, central carbon metabolites, phenolic compounds, glycerophospholipids, sterol lipids, polyketides, and related lipids, and unclassified other metabolites.

#### Monoculture Experiment Analysis

To visualize global metabolic variation across GIN and HA cells, a heatmap was generated using the *pheatmap* package in R (version 4.3.1). The data set used for this analysis corresponded to the monoculture AP-MALDI-MSI data set (GIN 8, GIN 28, GIN 31 vs HA). Following initial annotation against the HMDB and LIPID MAPS databases, a curated subset of metabolites and lipids (*n* = 53) was selected based on biological relevance and used for downstream analysis.

Prior to visualization, intensity values were log_10_-transformed and subjected to *z*-score normalization across each feature to center the data and facilitate comparative interpretation. Samples were arranged in a predefined group order without clustering to preserve the experimental structure, while metabolites were clustered using unsupervised hierarchical clustering based on Euclidean distance to reveal patterns of coabundance.

Principal Component Analysis (PCA) was conducted to visualize variance in metabolite abundance across experimental groups and to identify underlying patterns in the data. Prior to PCA, metabolite intensity values were log_10_-transformed to reduce right skewness, stabilize variance, and improve the normality of the data distribution. Pareto scaling was subsequently applied to moderate the dominance of high-intensity ions and preserve relative variance among metabolites.^25,26^ This approach has recently been shown in Orbitrap-based imaging data sets to enhance the interpretability of multivariate analyses compared with unscaled data.

To classify samples and identify discriminative features, a Random Forest (RF) model was implemented by using the *caret* and *randomForest* packages in R (version 4.3.1). Model training involved 10-fold cross-validation, repeated three times to ensure robust performance evaluation. Each model comprised 500 trees, with the number of variables randomly sampled at each split optimized automatically by cross-validation. Reproducibility was maintained by setting a fixed random seed (set.seed = 123). The classification model was evaluated based on the area under the receiver operating characteristic curve (AUC-ROC), providing a measure of model discrimination. Feature importance was assessed using both Mean Decrease in Accuracy (MDA) and Mean Decrease in Gini index (MDG) derived from a RF classifier.^27,28^ Features with MDA >1 and MDG >0 were considered significant contributors to class separation consistent with previous applications of RF in metabolomics. These thresholds were empirically supported by the distribution of feature scores within our data set and validated using permutation testing (100 iterations, *rfPermute* package).

Univariate statistical analysis was performed using MetaboAnalyst 6.0.^29^ To compare metabolite levels between monocultured GIN and HA cells, an unpaired, nonparametric Mann–Whitney *U* test was applied. This test was chosen due to the non-normal distribution of the data and differences in group variance, as assessed by Levene’s test. Benjamini–Hochberg correction was used to control the false discovery rate (FDR) associated with multiple testing. Metabolites were considered significantly altered if |log_2_FC| > 1.5 and FDR-adjusted *p* < 0.05.

#### Coculture Experiment Analysis

For coculture experiments, two comparisons were performed: (1) HA in coculture vs HA in monoculture and (2) GIN in coculture vs GIN in monoculture, using data across 10 replicates (i.e., 10 individual cells per cell line).

Multivariate and univariate analyses were performed using the same preprocessing workflow as that described for monoculture data. PCA was used to assess variance within each comparison group, followed by RF classification to identify discriminative features.

Univariate statistical analysis was performed using a Mann–Whitney *U* test with a Benjamini–Hochberg correction (FDR). In addition, pairwise comparisons between individual cell line combinations were conducted using the Kruskal–Wallis test. Metabolites were considered significantly altered if |log_2_FC| > 1.5 and FDR-adjusted *p* < 0.05.

### Liquid Chromatography–Mass Spectrometry (LC–MS) Analysis

LC–MS/MS was performed to validate and compare metabolites identified by AP-MALDI-MS, providing confirmation and increasing the confidence in metabolite assignments.

Monoculture (GIN and HA) and coculture samples were prepared under comparable experimental conditions to those used for AP-MALDI-MSI. Cells were cultured to a density of approximately 5 × 10^5^ cells in T75 flasks on the day of extraction. For coculture conditions, the HA/GIN ratio (70:30) was maintained consistent with AP-MALDI-MSI experiments.

Cells were fixed with 4% PFA for 15 min at room temperature, followed by sequential washes with PBS and sterile water to remove residual fixative and salts. To quench metabolism and initiate extraction, cells were treated with 500 μL of ice-cold (−20 °C) LC-MS-grade MeOH. Metabolites were extracted by manually scraping the cells and collecting the supernatant. The cell extracts were then incubated on a thermomixer at 4 °C and 2000 rpm for 1 h to facilitate metabolite release. Following incubation, samples were centrifuged at 13,000 × g for 5 min at 4 °C to separate the cellular debris from the metabolite-containing supernatant. The MeOH supernatants were collected and evaporated using a vacuum concentrator (SpeedVac; Thermo Fisher Scientific) at room temperature.

Dried extracts were then reconstituted in 70 μL of LC-MS-grade MeOH, followed by a second centrifugation at 13,000 × g for 10 min at 4 °C to remove any remaining particulate matter. The resulting supernatants were carefully transferred to LC–MS vials and stored at −80 °C until analysis.

Prior to LC–MS analysis, quality control (QC) samples were prepared by pooling 5 μL aliquots from each individual extract to assess the system stability and reproducibility across the run.

Samples were analyzed on a Q Exactive Orbitrap mass spectrometer (Thermo Fisher Scientific, Hemel Hempstead, UK) equipped with a HESI-II electrospray ionization source operating in the positive and negative ion switching mode. Full-scan MS data were acquired over an *m*/*z* range of 75–1050 at a resolution of 140,000 at *m*/*z* 200. Data-dependent MS/MS acquisition was conducted on pooled QC samples using a top-5 method at a resolution of 17,500 at *m*/*z* 200 with stepped normalized collision energies of 20, 30, and 40.

Chromatographic separation was achieved using a ZIC-pHILIC column (150 × 4.6 mm^2^, 5 μm particle size) maintained at 45 °C on a Dionex UltiMate 3000 LC system (Thermo Fisher Scientific, Hemel Hempstead, UK). Elution was performed using a solvent system consisting of phase A (10 mM ammonium carbonate in water) and phase B (acetonitrile). A linear gradient from 80% to 5% of mobile phase B was applied over 15 min, followed by a return to 80% B over 2 min and a 7 min re-equilibration at 80% B, giving a total run time of 24 min. The flow rate was 300 μL/min, the injection volume was 10 μL, and samples were maintained at 4 °C throughout.

Raw LC–MS data were processed using *Compound Discoverer* 3.3 SP2 (Thermo Fisher Scientific, Hemel Hempstead, UK). An untargeted analysis workflow was employed for peak detection, alignment, and preliminary compound annotation. Accurate mass values were matched against the HMDB and mzCloud spectral library (https://www.mzcloud.org/). For features with MS/MS spectra, compound identification was supported by spectral matching in mzCloud. To further support identification confidence, analytical standards were run alongside biological samples. These standards were used to confirm the retention times and MS/MS fragmentation patterns of selected metabolites, enabling assignment of Schymanski confidence levels.^32^

## Results and Discussion

### AP-MALDI-MSI Single-Cell Metabolomics Workflow

To enable single-cell level metabolic profiling, we developed and implemented a streamlined workflow integrating microscopy-guided cell identification with AP-MALDI-MSI. As illustrated in Figure 1A–D, cells were seeded onto chamber slides and fixed using 4% paraformaldehyde (PFA) to preserve spatial architecture prior to analysis. While PFA fixation can affect certain metabolites containing reactive functional groups such as amines and thiols, it was used here to stabilize cellular structure and minimize spatial metabolite redistribution during sample preparation.^30^ Following fixation, brightfield microscopy was used to capture cell morphology and guide image coregistration. After matrix application, AP-MALDI-MSI was performed by using high-resolution mass spectrometry at 10 μm spatial resolution.

**Figure 1:**
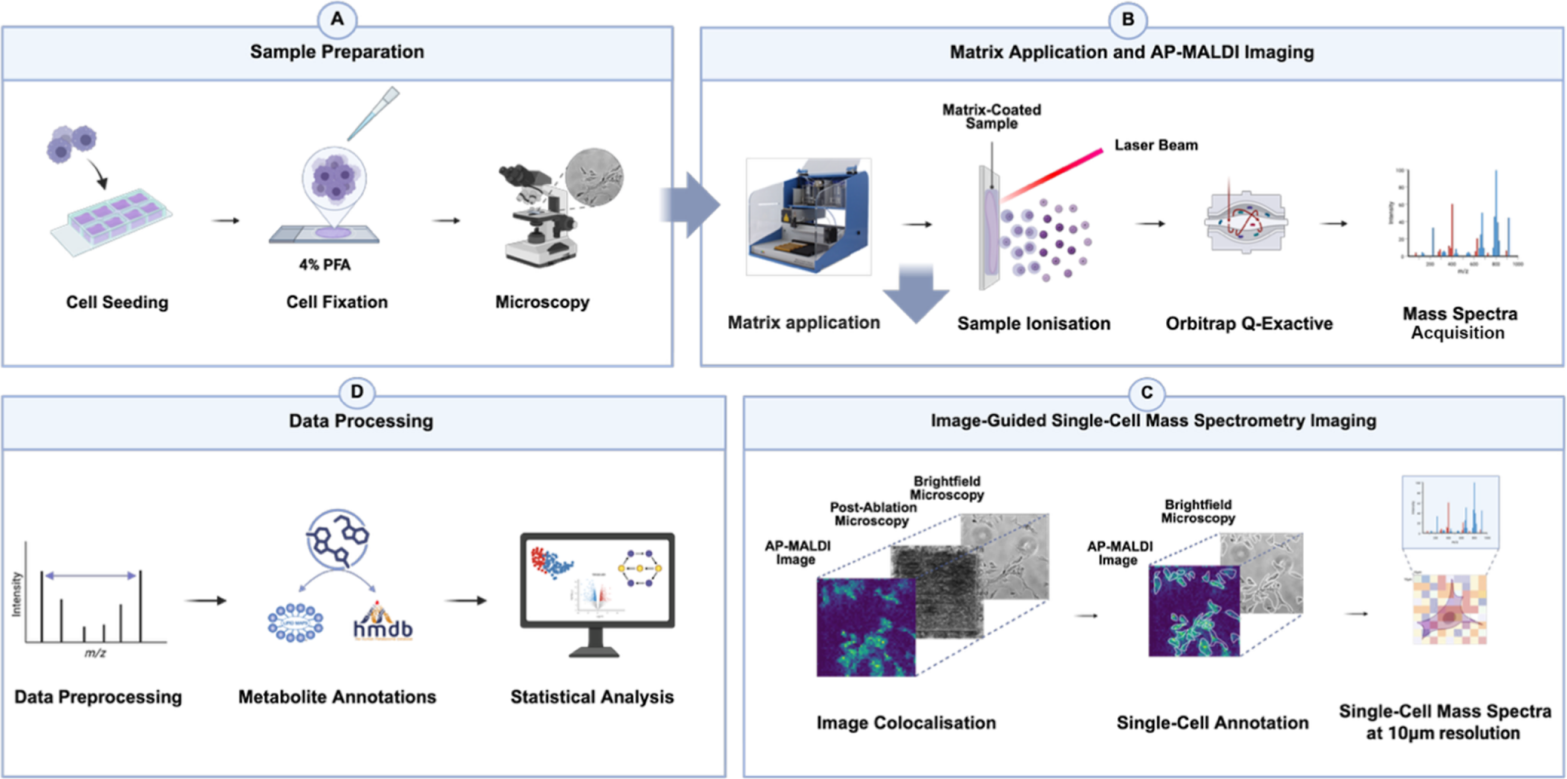
Workflow for AP-MALDI-MSI-based single-cell metabolomics. (A) Cells are seeded onto chamber slides, fixed using 4% PFA, and imaged using brightfield microscopy for cell identification. (B) Matrix is applied to the sample, followed by laser ablation and ionization to acquire AP-MALDI-MSI images. (C) Image registration is performed to align brightfield microscopy, ablated region, and AP-MALDI-MSI data, enabling extraction of single-cell metabolomes. (D) Data processing steps for generating and analyzing single-cell metabolic profiles.

Our workflow incorporated microscopy, both pre- and postablation, to precisely align ablated regions with microscopy images for single-cell identification. To integrate AP-MALDI-MSI data at the single-cell level, MSI data sets were processed and spatially registered in R using the *Cardinal* package, with adaptations for coordinate alignment and pixel-level intensity extraction from single cells. Preprocessing included TIC normalization, local-minima baseline reduction, and peak alignment at 5 ppm tolerance. Coordinate mapping between microscopy and MSI data sets was optimized to account for AP-MALDI-MSI discrete ablation geometry, enabling precise ion extraction from individual cells. Extracted features were subsequently annotated through MFP using in-house software developed at the School of Pharmacy, University of Nottingham, based on accurate mass.^22^ Although this approach has previously been applied in OrbiSIMS data sets, this study represents the first demonstration of its integration within AP-MALDI-MSI data for single-cell metabolomic analysis.

### Comparative Analysis of AP-MALDI-MSI Matrices for Single-Cell Metabolite Profiling

We first evaluated matrix selection for single-cell metabolomics using AP-MALDI-MSI by investigating three matrices: 2,5-dihydroxybenzoic acid (DHB), α-cyano-4-hydroxycinnamic acid (CHCA), and 9-aminoacridine (9AA). As shown in Figure 2A, DHB enabled clear identification of the matrix peak at *m*/*z* 155.0338 and a cell-specific metabolite peak at *m*/*z* 152.0566, corresponding to guanine. The spatial distribution of this ion delineated single-cell morphology, confirmed by overlaying the brightfield image with reduced opacity onto the AP-MALDI-MSI ion image. We performed parallel analyses using CHCA and 9AA to compare metabolite coverage and chemical class representation, as shown in Figure 2B,C and Table S1. While each matrix generated distinct profiles, CHCA and DHB exhibited the greatest overlap in detected features, followed by DHB and 9AA. These results are consistent with previous studies reporting matrix-dependent selectivity in metabolite detection.^31^ The chemical class distribution revealed further matrix-specific trends; DHB and CHCA primarily enriched amino acids and their derivatives, whereas 9AA preferentially ionized sterol lipids, Figure 2D. Importantly, DHB yielded the highest number of metabolite features overall (*n* = 218), compared to CHCA (*n* = 147) and 9AA (*n* = 108). To enable broad metabolomic coverage encompassing both small polar metabolites (e.g., amino acids) and diverse lipid classes at the single-cell level, DHB was selected as the matrix for subsequent comparisons between glioblastoma invasive margin cells (GIN) and astrocytes (HA). This systematic optimization provides the first direct comparison of matrix-dependent metabolite coverage at the single-cell scale in ambient conditions, offering practical guidance for future single-cell workflows. Having established DHB as the optimal matrix, we next compared single-cell AP-MALDI-MSI with conventional bulk LC–MS to characterize differences in metabolite coverage and to evaluate whether cross-platform overlap can be used to increase confidence in metabolite assignments.

**Figure 2:**
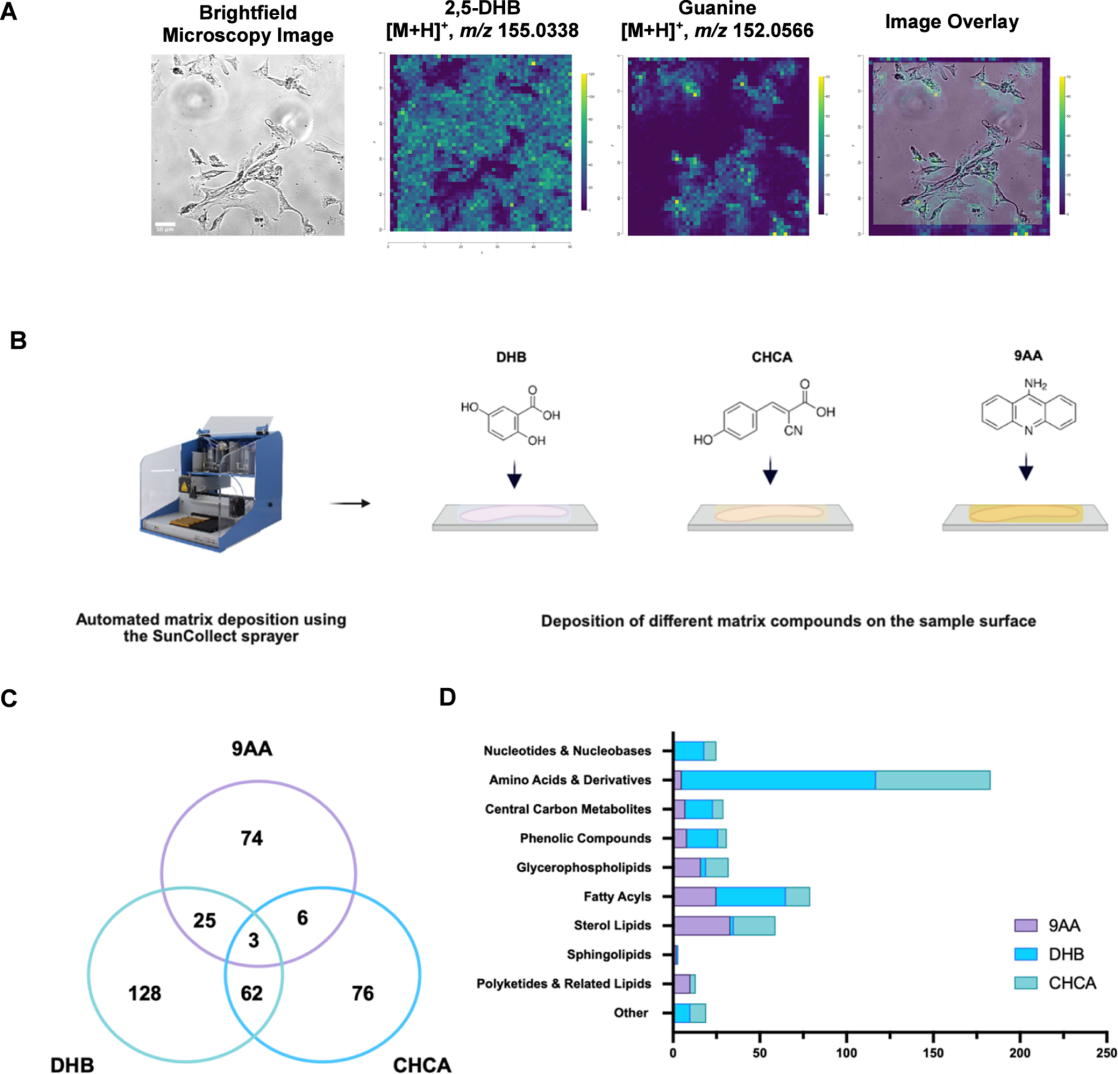
Comparative analysis of metabolite detection in human cortical astrocytes using AP-MALDI-MSI with different matrices. (A) Brightfield microscopy, magnification ×10 and AP-MALDI-MSI ion images representing the DHB matrix peak at *m*/*z* 155.0338, followed by cell-specific marker guanine at *m*/*z* 152.0566, and AP-MALDI-MSI ion image and brightfield microscopy image overlay to illustrate spatial metabolite distribution in HA. (B) Schematic of automated matrix deposition using the SunCollect sprayer and chemical structures of DHB, CHCA, and 9AA. (C) Venn diagram showing the overlap of metabolites detected using AP-MALDI-MSI with DHB, CHCA, and 9AA matrices. (D) Bar graph comparing the number and class of metabolites detected with different matrices across various compound categories.

When comparing HA cells analyzed by AP-MALDI-MSI with bulk populations of approximately 5 × 10^5^ cells profiled by LC–MS as shown in Figure 3A, we observed higher number of metabolite annotations in the bulk LC–MS method compared to the single-cell AP-MALDI-MSI method, Figure 3B,C. Nevertheless, the single-cell AP-MALDI-MSI workflow yielded over a hundred annotated metabolites, representing a remarkably high number relative to sample size. We observed limited overlap between platforms, with 19 shared metabolites detected by both, Figure 3B. LC–MS predominantly captured polar metabolites, including amino acids and derivatives and central carbon metabolites, Figure S1, whereas AP-MALDI-MSI detected amino acid and lipid species, as shown earlier in Figure 2D. To improve identification confidence, we acquired LC–MS/MS annotating structural assignments for metabolites initially observed by AP-MALDI-MSI, Table S1.^32^ The limited overlap of metabolites between two techniques observed is consistent with previous reports, reflecting intrinsic differences in ionization efficiency, detection sensitivity, and chromatographic separation associated with the LC method.^33^

**Figure 3:**
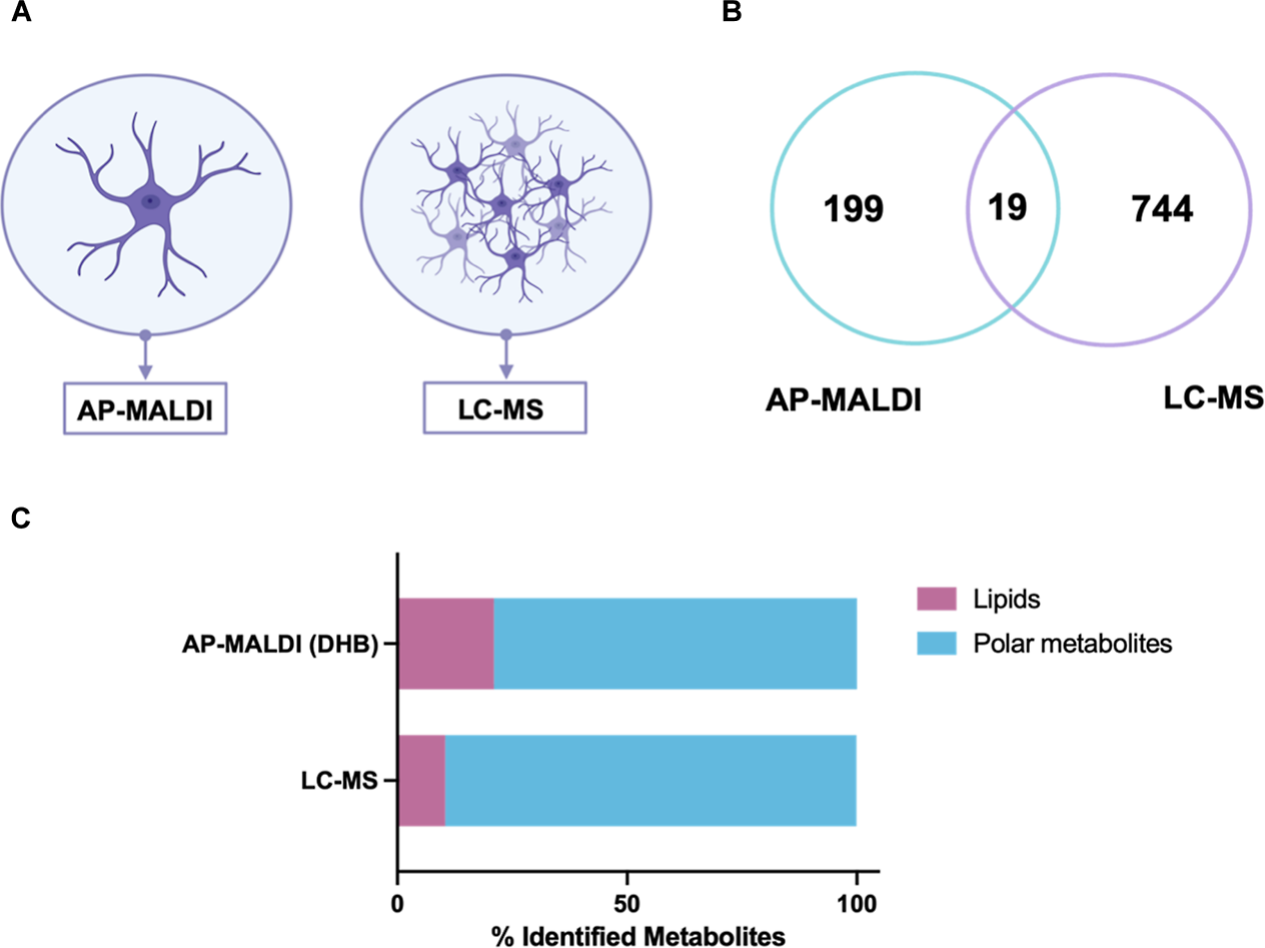
Comparative overview of AP-MALDI-MSI and LC–MS metabolite detection. (A) Schematic representation of sample input: AP-MALDI-MSI analysis of a single astrocyte versus LC–MS analysis of 5 × 10^5^ astrocytes. (B) Venn diagram showing the number of metabolites uniquely detected by LC–MS (*n* = 744), AP-MALDI-MSI with the DHB matrix (*n* = 199) and the overlap between platforms (*n* = 19). (C) Distribution of single cell-identified metabolites by chemical class, highlighting differences in lipid (violet) and polar metabolite (blue) coverage.

Overall, these results establish a robust single-cell AP-MALDI-MSI workflow capable of a broad, untargeted metabolite profile from single cells under ambient conditions, spanning small polar metabolites to multiple lipid subclasses. While bulk LC–MS remains advantageous for broader polar metabolite coverage, our cross-platform evaluation demonstrates that single-cell AP-MALDI-MSI uniquely enables cell-specific metabolic investigation with spatial context, which is an analytical capability not achievable by bulk methods. This foundational optimization provides the analytical basis for subsequent studies to investigate metabolic interactions between different cell types in coculture.

### Metabolite Profiling Reveals Cell-Specific Signatures for Astrocyte and GBM Single-Cell Annotations

We then performed a comparative analysis to investigate metabolic differences between HA and glioblastoma invasive margin cells (GIN) using AP-MALDI-MSI (Figure S3A−C. GIN cells exhibited broader intragroup variance than HA cells, as indicated by their wider distribution along PC1 (28.39%) and PC2 (24.06%), reflecting greater metabolic heterogeneity within the invasive tumor population. This is consistent with the pronounced heterogeneity reported in invasive glioblastoma populations, where metabolic plasticity supports adaptation and therapeutic resistance.^34,35^ In contrast, the tight clustering of HA cells reflects the more uniform metabolic profile of HA, whose core functions in glucose metabolism, neurotransmitter cycling, and energy support are conserved across individual cells.^36^

To further investigate metabolite-level variation between GIN and HA cells, we generated a heatmap of selected annotated metabolites and lipids (*n* = 53) with known biological relevance (Figure 4). Metabolites included in the heatmap were prioritized through a biologically guided selection step (see the Experimental Section) based on endogenous mammalian metabolism and cancer-associated pathways. The complete list of annotated features, including both retained and excluded compounds, is provided in Table S2.

**Figure 4:**
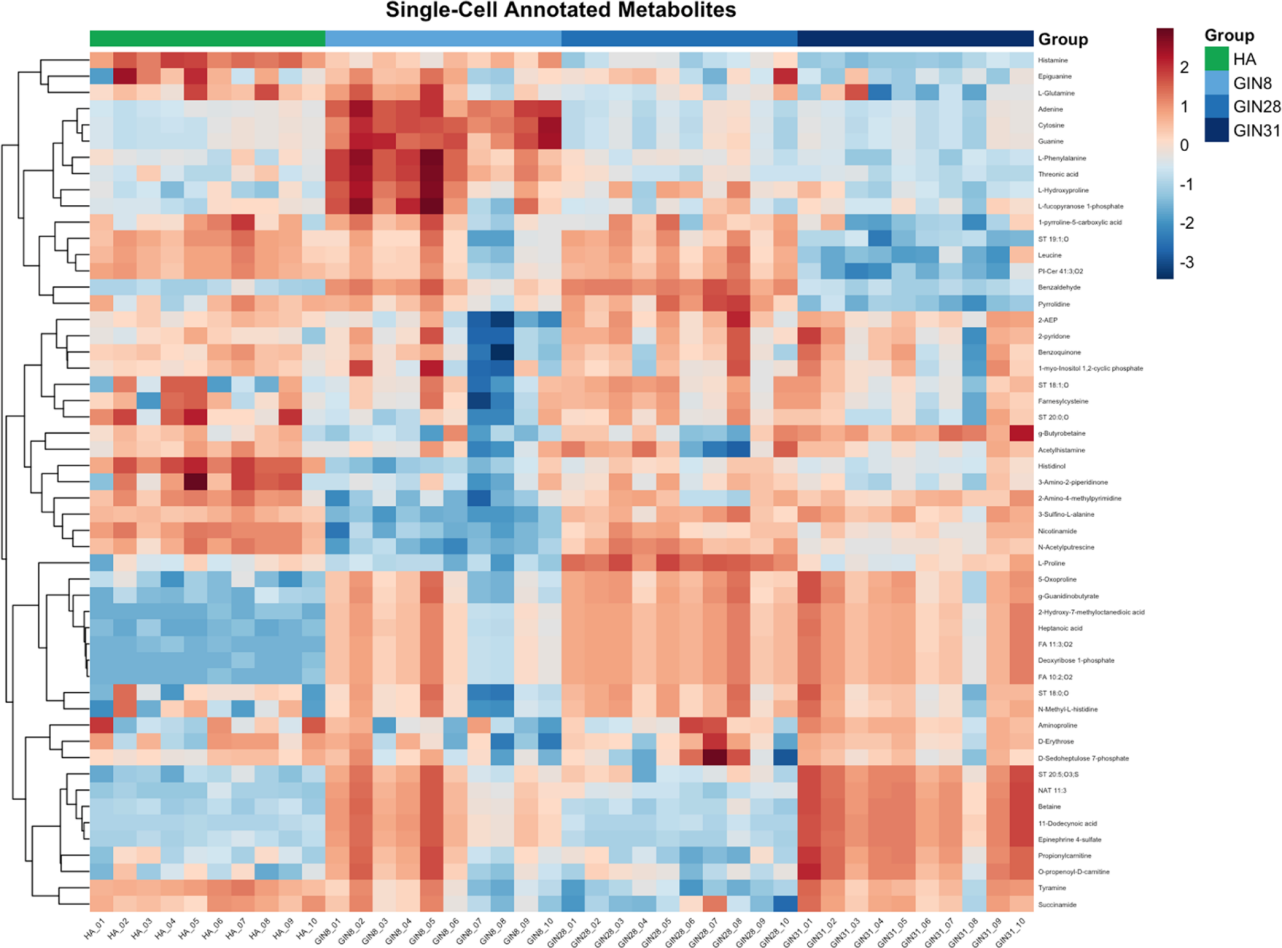
Heatmap showing the distribution of annotated metabolites in individual GIN cells (GIN 8, 28, and 31, *n* = 10) and HA cells (*n* = 10). Data were *z*-score normalized across each metabolite to facilitate comparison between samples. Colors represent relative metabolite abundance (red = high and blue = low). Hierarchical clustering based on Euclidean distance grouped metabolites and cells by similarity.

Hierarchical clustering based on Euclidean distance was applied to reveal patterns of coabundance and cell-to-cell variability within each population. This visualization revealed pronounced metabolic heterogeneity among GIN cells, characterized by diverse intensity patterns across individual cells from the same population. In contrast, HA cells exhibited a more uniform and tightly clustered metabolite profile, consistent with lower intragroup variability. These findings highlight the advantage of single-cell metabolomics in capturing cell-to-cell metabolic diversity that would be obscured in bulk analyses—thus providing important insights into the heterogeneity of tumor-associated metabolic reprogramming. While single-cell transcriptomic and genomic approaches are comparatively more advanced and have increasingly revealed cellular heterogeneity in tumors, single-cell metabolomics remains an emerging field.^37,38^ Our study, conducted in cell culture models, demonstrates that spatially resolved metabolic profiling at the single-cell level can uncover metabolic heterogeneity, offering a novel layer of information. This approach lays important groundwork for future applications in tissue samples, where it could further illuminate tumor biology in situ.

Building on these findings, we next examined how these metabolites were spatially distributed within individual cells using AP-MALDI-MSI, enabling the identification of characteristic biochemical markers.

As shown in Figure 5A, nucleotides and nucleobases were detected in both cell types, with guanine displaying particularly distinct spectral features. Guanine is a purine nucleobase, and elevated purine metabolism has been implicated in cell proliferation and therapeutic resistance of GBM cells, particularly through enhanced DNA repair pathways.^33^ Its consistent detection in both HA and GIN cells may reflect the fundamental role of purine turnover in brain cell function while also hinting at metabolic pathways that GBM may exploit for survival and growth. Figure 5B presents the amino acids and their derivatives identified across both HA and GIN cells, among which acetylhistamine emerged as the most spatially distinct and consistently detected metabolite. Histamine and its derivatives play a role in neuromodulation, vascular regulation, and sustaining the brain microenvironment, making acetylhistamine a plausible candidate biomarker for processes shared across healthy and malignant glial cells.^33,39^ Similarly, Figure 5C illustrates lipid species detected in the two cell types including sterol-related compounds present in both populations. Sterols play essential roles in membrane structure, signaling, and cellular homeostasis in the brain.^40^ The consistent detection of these lipid species across both astrocytes and GIN cells highlights their potential relevance in distinguishing cellular metabolic states. Consequently, guanine, acetylhistamine, and identified sterol lipids were chosen as reference ions because they were reproducibly detected in both astrocytes and GBM cells, showed distinct spatial localization, and are linked to key metabolic functions in brain cells. Together, these findings established a set of reproducible metabolic markers that enabled confident single-cell identification and mapping within AP-MALDI-MSI data sets. Building on this foundation, we next applied this approach to a coculture model to explore how spatial interactions between GBM and astrocytes influence their metabolic landscape.

**Figure 5:**
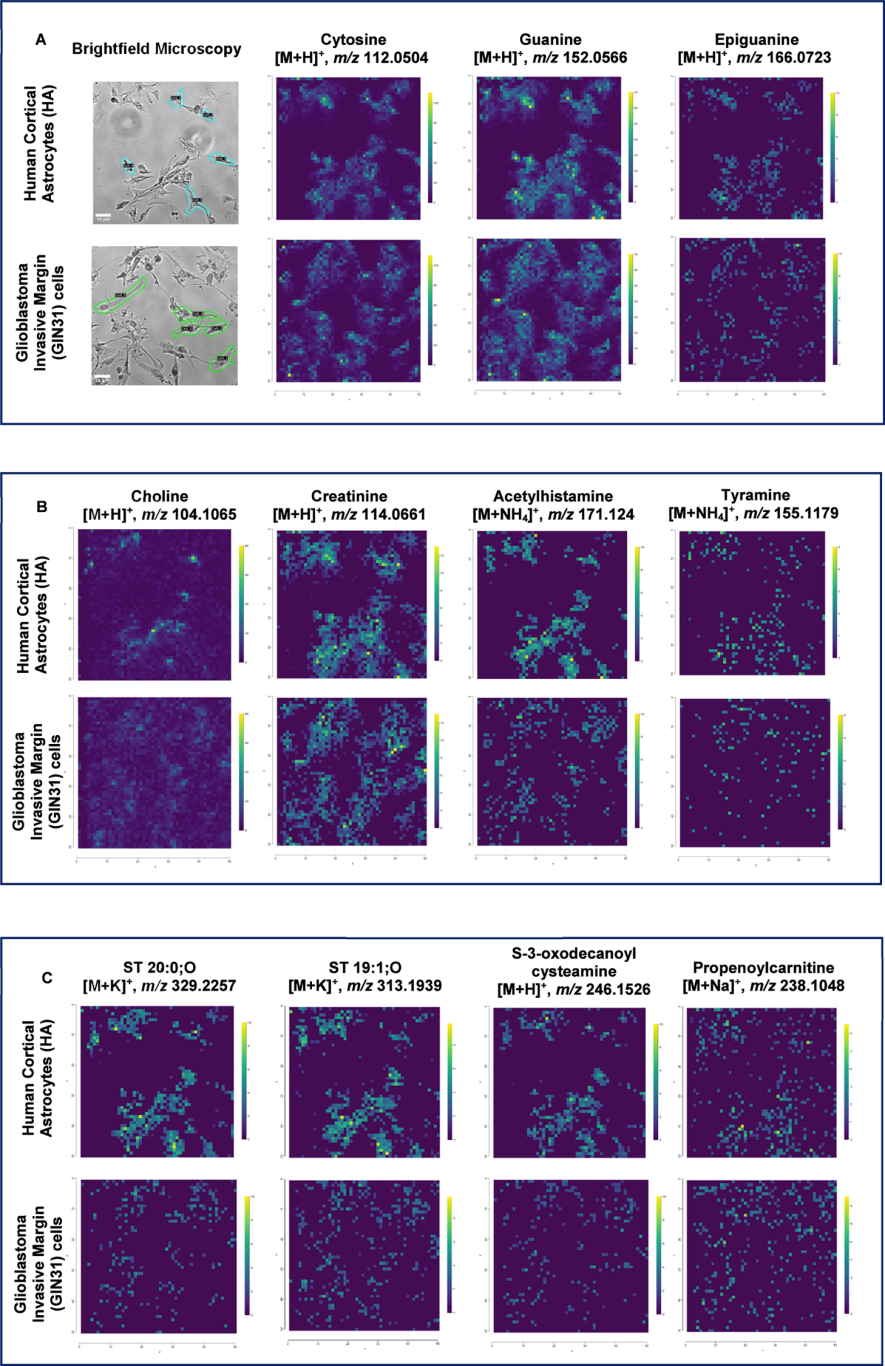
Identified metabolite and lipid classes in single cells of human cortical astrocytes and glioblastoma invasive margin cells using the DHB matrix. (A) Panel of brightfield microscopy images showing representative single-cell ROI selected for metabolite extraction, alongside corresponding AP-MALDI-MSI ion images of selected nucleotides and nucleobases. (B) Panel of AP-MALDI-MSI ion images displaying annotated amino acids and derivative metabolites. (C) Panel of AP-MALDI-MSI ion images displaying lipid annotations.

### Single-Cell Spatial Metabolomics in a Coculture Model of Glioblastoma and Astrocytes

Having identified distinct metabolic signatures in isolated cell populations, we next applied the single-cell AP-MALDI-MSI methodology to explore metabolic crosstalk between HA and GIN cells in a coculture setting, thus assessing feasibility for future application to a primary tumor tissue composed of heterogeneous disease/nondisease cell types and cellular states. Astrocytes are the most abundant glial cell type in the brain and play a key role in maintaining the tumor microenvironment.^41,42^ To mimic early-stage GBM infiltration, we established a mixed population composed of 70% astrocytes and 30% GBM patient-derived invasive margin cells.^17,18^ This model enabled direct investigation of metabolite-level interactions between the two cell types within a shared microenvironment while retaining single-cell spatial resolution (Figure 6 A–D).

**Figure 6:**
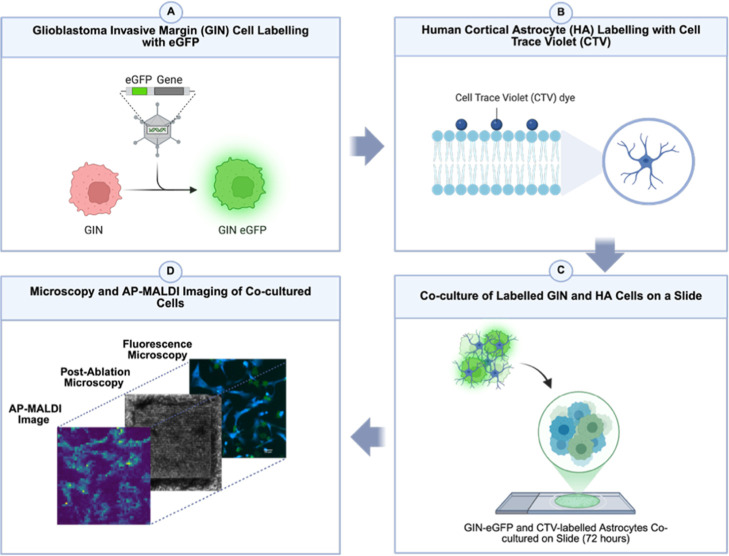
Application of single-cell AP-MALDI-MSI to investigate a metabolic crosstalk in a coculture of GIN and HA cells. (A) GIN cells were genetically tagged with enhanced green fluorescent protein (eGFP). (B) HA cells were labeled with CellTrace violet dye for identification. (C) Coculture of HA (CTV-labeled) and GIN (eGFP-expressing) cells was established on chamber slides. (D) AP-MALDI-MS image overlaid with postablation brightfield microscopy (10× magnification) and fluorescence microscopy images to enable single-cell analysis.

To enable precise identification of each cell type within the mixed culture, GIN cells were genetically tagged with eGFP, while HA cells were labeled with CellTrace Violet (CTV) membrane dye, allowing fluorescence-based single-cell discrimination. Integrating this dual-labeling strategy with AP-MALDI-MSI enabled direct correlation between fluorescence identity and metabolic profile of individual cells, establishing a robust framework for spatial single-cell metabolomics.^43^ The two cell types were cocultured on chamber slides for 5 days, followed by fixation with 4% PFA. Fluorescence microscopy and postablation brightfield imaging were combined with AP-MALDI-MSI to extract metabolic profiles at single-cell resolution.

While previous studies have explored metabolic communication in coculture models using mass spectrometry imaging, such as the work by Zhang et al. on Esophageal Squamous Cell Carcinoma cell–fibroblast interactions using MALDI-TOF MSI, their Transwell system lacked direct cell–cell contact and offered lower mass resolution and chemical specificity. In contrast, our approach integrates direct physical coculture with fluorescence-guided single-cell discrimination and a high-resolution mass analyzer, providing a unique platform for spatially resolved metabolic profiling of different cell populations.

To gain insight into spatially distinct metabolic features associated with cell–cell interactions, we examined the distribution patterns of annotated metabolites under coculture conditions. This exploratory analysis revealed several metabolites with markedly different localizations. As shown in Figure 7, metabolites such as cytosine (*m*/*z* 112.0504), adenine (*m*/*z* 136.0618), 2-aminohistamine (*m*/*z* 112.0868), and acetylcadaverine (*m*/*z* 145.1335) were primarily confined within cellular boundaries, indicative of intracellular retention and supporting spatially resolved metabolite detection, whereas metabolites such as 3-amino-2-piperidone (*m*/*z* 115.0865) and *N*-acetylputrescine (*m*/*z* 131.1179) were detected both within and along the periphery of cells. While such distributions may reflect extracellular metabolites arising from secretion or intercellular signaling processes, we acknowledge that some metabolite delocalization during sample preparation or matrix deposition cannot be fully excluded.^44,45^

**Figure 7:**
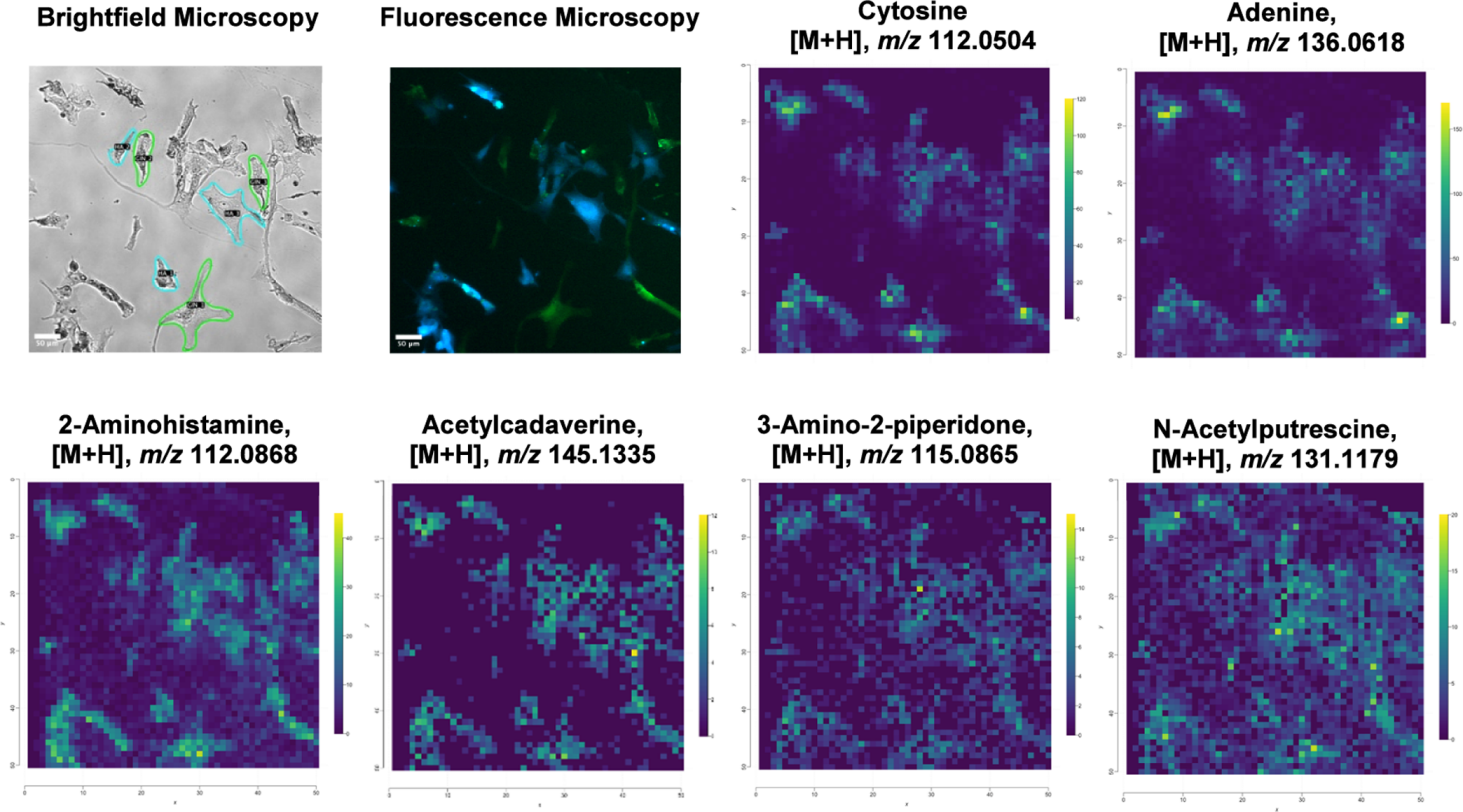
Metabolomic analysis of GIN and HA single cells from coculture. Representative panel showing brightfield microscopy, fluorescence microscopy, and AP-MALDI images used for accurate cell annotation and extraction of individual single-cell metabolomes. In fluorescence microscopy, glioblastoma cells (eGFP^+^) are shown in green and astrocytes (CTV^+^) in blue. In brightfield microscopy, selected HA–GIN cell pairs used for analysis are outlined to indicate the regions of interest.

Building on these spatial observations, we next examined how coculture conditions influenced the overall metabolic profile of GIN cells. In Figure 8A, PCA illustrates the distribution of GIN cell metabolomes under coculture and monoculture conditions. While both groups exhibit some overlap indicating shared core metabolic features, GIN cells in coculture display a broader distribution across both principal components. This dispersion may reflect increased metabolic heterogeneity arising from intercellular interactions with astrocytes, potentially representing early metabolic adaptation or signaling-driven shifts in tumor cell metabolism.

**Figure 8:**
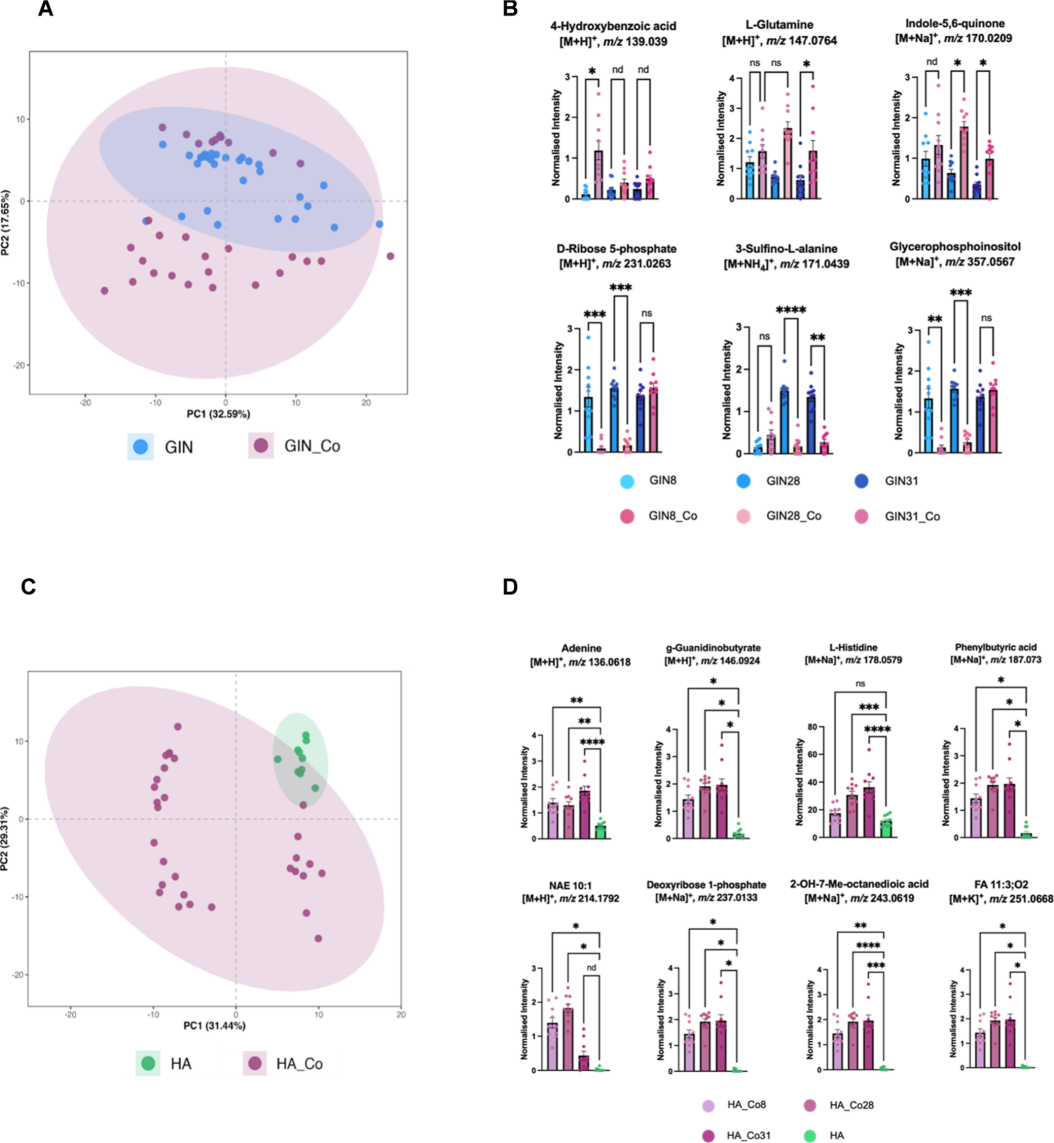
Comparison of GIN and HA cell metabolic profiles cultured in coculture versus monoculture. (A) PCA illustrating metabolic variation between GIN cells cultured alone (*n* = 10) and those cocultured with astrocytes (*n* = 10). (B) Significantly altered metabolites identified via univariate analysis (*p* < 0.05) and RF feature selection. Data are presented as mean ± SEM. (C) PCA plot showing metabolic differences between HA cells in monoculture (*n* = 10) and in coculture with GIN cells (*n* = 10). (D) Metabolites significantly altered under coculture conditions, identified using univariate statistics (*p* < 0.05) and RF feature selection. Data are presented as mean ± SEM.

Subsequent statistical analysis identified metabolites and lipids that were differentially abundant in GIN cells under coculture conditions relative to monoculture (Figure 8B, Table 1, Table S3). d-Ribose 5-phosphate (*m*/*z* 231.0263) and glycerophosphoinositol (*m*/*z* 357.0567) were significantly altered in cocultured GIN cells, suggesting enhanced nucleotide turnover and phospholipid-related signaling in the presence of astrocytes. This metabolic shift, associated with proliferative activity, is consistent with previous studies of astrocyte–glioblastoma crosstalk, such as studies demonstrating that mitochondrial transfer from astrocytes to glioblastoma cells promotes proliferation and tumorigenicity.^46,47^ Similarly, l-glutamine (*m*/*z* 147.0764) and indole-5,6-quinone (*m*/*z* 170.0209) levels were elevated in coculture, suggesting broader metabolic adaptations associated with tumor–astrocyte interactions. While glutamine supports central biosynthetic and bioenergetic processes, indole-derived species may reflect altered indole-associated chemistry, potentially consistent with previous evidence implicating tryptophan metabolism in GBM survival.^48^ Conversely, 3-sulfino- *l* -alanine (*m*/*z* 171.0439) showed a marked decrease in coculture, which may indicate reduced sulfur amino acid metabolism or an altered redox state. As an intermediate in cysteine catabolism, 3-sulfino- *l* -alanine plays a key role in the biosynthesis of hypotaurine and taurine, molecules involved in cellular redox regulation.^49,50^

**Table 1:** Significant Metabolites Identified in GIN Coculture (*n* = 10) with HA (*n* = 10). Metabolites Were Detected Using AP-MALDI Mass Spectrometry with the DHB Matrix. Metabolites Were Selected Using Variable Importance Measures from RF Analysis (Mean Decrease Accuracy and Mean Decrease Gini), Adjusted *p*-Values from Statistical Testing, and log_2_ Fold Change (log_2_FC) Values

metabolite name	HMDB/LIPID MAPS ID	formula	adduct	observed *m*/*z*	theoretical *m*/*z*	mass error	mean decrease accuracy	mean decrease gini	adjusted *p* value	log2(FC)
4-Hydroxybenzoic acid	HMDB0000500	C_7_H_7_O_3_	[M + H]^+^	139.0390	139.0390	0.21	0.6163	0.0998	1.0187 × 10^–4^	1.7788
l-Glutamine	HMDB0000641	C_5_ H_11_N_2_O_3_	[M + H]^+^	147.0764	147.0764	–0.13	1.6256	0.1061	2.6686 × 10^–5^	1.1162
Indole-5, 6-quinone	HMDB0006779	C_8_H_5_NO_2_Na	[M + K]^+^	170.0209	170.0214	–2.94	1.2952	0.0532	1.2432 × 10^–4^	1.0528
3-Sulfino- *l* -alanine	HMDB0000192	C_3_ H_11_N_2_O_4_S	[M + NH_4_]^+^	171.0439	171.0434	2.92	1.3623	0.0367	1.5132 × 10^–3^	–1.719
d-Ribose 5-phosphate	HMDB0001548	C_5_H_12_O_8_P	[M + H]^+^	231.0263	231.0264	–0.43	1.0010	0.0247	9.0840 × 10^–4^	–1.2105
Glycerophosphoinositol	HMDB0004249	C_9_H_19_O_11_PNa	[M + Na]^+^	357.0567	357.0557	2.80	1.5342	0.0732	1.2675 × 10^–3^	–1.1384

As shown in Figure 8C, we similarly examined the metabolic profiles of HA in coculture relative to monoculture conditions. Astrocytes grown in monoculture clustered more tightly, consistent with a uniform metabolic phenotype in the absence of tumor-derived influence. In contrast, astrocytes in coculture displayed broader variance and partial separation, particularly along PC2, suggesting metabolic change in response to the GBM microenvironment. Previous studies have shown that glioblastoma cells in coculture with astrocytes can undergo transcriptional reprogramming, metabolic rewiring, and enhanced proliferative and invasive behaviors in response to astrocytic cues.^51^ However, delineating specific metabolic changes within each cell population remains experimentally challenging and is not yet well established. The partial overlap along PC1 indicates retention of core metabolic features, consistent with previous reports that astrocytes maintain fundamental phenotypic characteristics in glioma coculture models reflecting shared metabolic profiles.^52^

To identify metabolites that were significantly altered in astrocytes during interaction with GBM cells, we compared metabolite intensities between HA cells in coculture and monoculture (Figure 8D, Table 2, Table S4). Nucleoside- and nucleotide-related metabolites, such as deoxyribose 1-phosphate (*m*/*z* 237.0133) and adenine (*m*/*z* 136.0618), showed significant increases in coculture, suggesting altered nucleotide turnover. Amino acid derivatives such as guanidinobutyrate (*m*/*z* 146.0924), histidine (*m*/*z* 178.0579), and phenylbutyric acid (*m*/*z* 187.073) were also elevated, potentially reflecting responses to oxidative stress, nitrogen metabolism, or protein turnover.^53,54^ Notably, lipid-related metabolites including NAE 10:1 (*m*/*z* 214.1792) and FA 11:3; O2 (*m*/*z* 251.0668) were significantly more abundant in cocultured astrocytes. These changes may indicate remodeling of membrane lipids and activation of inflammatory signaling pathways, consistent with evidence that astrocyte lipid metabolism contributes to brain pathology, including GBM.^55,56^

**Table 2:** Significant Metabolites Identified in HA Coculture (*n* = 10) with GIN Cells (*n* = 10). Metabolites Were Detected Using AP-MALDI Mass Spectrometry with the DHB Matrix. Metabolites Were Selected Using Variable Importance Measures from RF Analysis (Mean Decrease Accuracy and Mean Decrease Gini), Adjusted *p*-Values from Statistical Testing, and log_2_ Fold Change (log_2_FC) Values

metabolite name	HMDB/LIPID MAPS ID	formula	adduct	observed *m*/*z*	theoretical *m*/*z*	mass error	mean decrease accuracy	mean decrease gini	adjusted *p* value	log2(FC)
Adenine	HMDB0000034	C_5_H_6_N_5_	[M + H]^+^	136.0618	136.0617	0.73	1.0010	0.0336	6.2735 × 10^–5^	1.2344
Guanidinobutyrate	HMDB0003464	C_5_H_12_N_3_O_2_	[M + H]^+^	146.0924	146.0924	–0.02	1.0010	0.0364	2.0211 × 10^–6^	1.5874
*l* -Histidine	HMDB0000177	C_6_H_9_N_3_O_2_Na	[M + Na]^+^	178.0579	178.0586	–3.93	1.2309	0.0558	1.0555 × 10^–7^	3.3216
Phenylbutyric Acid	HMDB0000543	C_10_H_12_O_2_Na	[M + Na]^+^	187.0730	187.0729	0.53	1.9757	0.1186	1.9174 × 10^–5^	3.4169
NAE 10:1	LMFA08040004	C_12_H_24_NO_2_	[M + H]^+^	214.1792	214.1801	–4.20	1.0010	0.0279	5.4794 × 10^–5^	5.6035
Deoxyribose 1-phosphate	HMDB0001351	C_5_H_11_O_7_PNa	[M + Na]^+^	237.0133	237.0134	–0.42	1.0010	0.0279	1.9174 × 10^–5^	5.5414
2-Hydroxy-7-methyloctanedioic acid	HMDB0000403	C_9_H_16_O_5_K	[M + K]^+^	243.0619	243.0629	–4.11	1.0010	0.0351	1.9174 × 10^–5^	5.2665
FA 11:3; O2	No ID match	C_11_H_16_O_4_K	[M + K]^+^	251.0668	251.0680	–4.78	1.3441	0.0615	1.9174 × 10^–5^	5.8797

Comparison of the two cell populations in coculture highlights potential metabolic crosstalk. Both GIN cells and HA cells in coculture exhibited evidence of altered nucleotide turnover, with increased ribose-5-phosphate and glycerophosphoinositol in tumor cells paralleled by elevated deoxyribose 1-phosphate and adenine in HA. Similarly, lipid-related changes were observed in both cells: GIN cells showed reduced phosphoinositol accumulation, whereas astrocytes displayed increased abundance of NAE 10:1 and unsaturated fatty acid consistent with reciprocal remodeling of membrane and signaling lipids. Furthermore, the detection of 4-hydroxybenzoic acid in GIN cells together with phenylbutyric acid enrichment in astrocytes points to complementary regulation of tyrosine metabolism across the two populations. This pattern aligns with reports of metabolic support from the tumor microenvironment, while the specific role of astrocytes in modulating amino acid availability remains to be determined.^57^

## Conclusions

This study establishes a microscopy-guided AP-MALDI-MSI workflow that enables single-cell metabolomic profiling while preserving cell-type identity within the coculture model. By integrating cell-specific labeling, high-resolution mass spectrometry, and a tailored annotation strategy, this platform allows untargeted, spatially resolved analysis of intercellular metabolic interactions.

Application of this workflow to glioblastoma-astrocyte cocultures revealed distinct and complementary metabolic alterations across cell types, including altered nucleotide turnover, phospholipid metabolism, and modulation of tyrosine pathway. These findings suggest coordinated metabolic exchange between glioblastoma and astrocytes within the brain microenvironment, potentially influencing tumor cell energy balance and providing new insight into how astrocytes contribute to tumor invasiveness. Integrating these findings with functional studies will further extend the potential to uncover the mechanistic underpinnings in disease progression.

Beyond the coculture model, this workflow provides an accessible framework for investigating cell–cell metabolic interactions within more complex tissue environments and across diverse biological systems. Extending this approach to primary tumor samples and other multicellular models will allow direct exploration of metabolic relationships in their native microenvironments, advancing our understanding of how spatial metabolic organization contributes to disease pathophysiology and therapeutic response.

## Supplementary Material



## Data Availability

The data that support the findings of this study are available from the corresponding author upon reasonable request.

## Notes

Single-cell metabolites identified by AP-MALDI-MSI (DHB matrix) with LC–MS/MS-supported annotations (RT and/or MS/MS); class distribution of LC–MS-identified metabolites; spatial alignment of microscopy and AP-MALDI-MSI for single-cell ROI selection; statistical analyses of HA and GIN single-cell data sets; metabolites detected in the monoculture; and metabolites identified in the coculture (GIN cells and astrocytes, respectively), with LC–MS/MS support where available (PDF)

## References

[1] Johnson C. H., Ivanisevic J., Siuzdak G. (2016). Metabolomics: Beyond Biomarkers and
towards Mechanisms. Nat. Rev. Mol. Cell Biol..

[2] Wang Z., Ge S., Liao T., Yuan M., Qian W., Chen Q., Liang W., Cheng X., Zhou Q., Ju Z., Zhu H., Xiong W. (2025). Integrative Single-Cell Metabolomics and Phenotypic
Profiling Reveals Metabolic Heterogeneity of Cellular Oxidation and
Senescence. Nat. Commun..

[3] Kompauer M., Heiles S., Spengler B. (2017). Atmospheric
Pressure MALDI Mass Spectrometry
Imaging of Tissues and Cells at 1.4-Μm Lateral Resolution. Nat. Methods.

[4] Zhang H., Shi X., Lu H., Li L. (2025). Delineation of Subcellular Molecular
Heterogeneity in Single Cells via Ultra-Low Flow Rate Desorption Electrospray
Ionization Mass Spectrometry (u-DESI-MS). Anal.
Chem..

[5] Marques C., Friedrich F., Liu L., Castoldi F., Pietrocola F., Lanekoff I. (2023). Global and Spatial
Metabolomics of Individual Cells
Using a Tapered Pneumatically Assisted Nano-DESI Probe. J. Am. Soc. Mass Spectrom..

[6] Passarelli M. K., Ewing A. G. (2013). Single-Cell Imaging Mass Spectrometry. Curr. Opin. Chem. Biol..

[7] Zhang L., Vertes A. (2018). Single-Cell Mass Spectrometry Approaches
to Explore
Cellular Heterogeneity. Angew. Chem., Int. Ed..

[8] Keller C., Maeda J., Jayaraman D., Chakraborty S., Sussman M. R., Harris J. M., Ané J. M., Li L. (2018). Comparison of Vacuum Maldi and Ap-Maldi Platforms for the Mass Spectrometry
Imaging of Metabolites Involved in Salt Stress in Medicago Truncatula. Front. Plant Sci..

[9] Chen B., OuYang C., Tian Z., Xu M., Li L. (2018). High Resolution
Atmospheric Pressure Matrix-Assisted Laser Desorption/Ionization-Quadrupole-Orbitrap
MS Platform Enables In Situ Analysis of Biomolecules by MultiMode
Ionization and Acquisition. Anal. Chim. Acta.

[10] Buchberger A. R., DeLaney K., Johnson J., Li L. (2018). Mass Spectrometry
Imaging:
A Review of Emerging Advancements and Future Insights. Anal. Chem..

[11] Nisar M. S., Zhao X. (2020). High Resolution Mass Spectrometry
for Single Cell Analysis. Int. J. Mass Spectrom..

[12] Perry R. H., Cooks R. G., Noll R. J. (2008). Orbitrap Mass Spectrometry:
Instrumentation,
Ion Motion and Applications. Mass Spectrom.
Rev..

[13] Rappez L., Stadler M., Triana S., Gathungu R. M., Ovchinnikova K., Phapale P., Heikenwalder M., Alexandrov T. (2021). SpaceM Reveals
Metabolic States of Single Cells. Nat. Methods.

[14] Delafiori J., Shahraz M., Eisenbarth A., Hilsenstein V., Drotleff B., Bailoni A., Wadie B., Ekelöf M., Mattausch A., Alexandrov T. (2025). HT SpaceM:
A High-Throughput and
Reproducible Method for Small-Molecule Single-Cell Metabolomics. Cell.

[15] Zhang Y., Shi M., Li M., Qin S., Miao D., Bai Y. (2025). Dynamic Single-Cell
Metabolomics Reveals Cell-Cell Interaction between Tumor Cells and
Macrophages. Nat. Commun..

[16] Smith S. J., Rowlinson J., Estevez-Cebrero M., Onion D., Ritchie A., Clarke P., Wood K., Diksin M., Lourdusamy A., Grundy R. G., Rahman R. (2020). Metabolism-Based Isolation of Invasive
Glioblastoma Cells with Specific Gene Signatures and Tumorigenic Potential. Neurooncol. Adv..

[17] Finotello F., Mayer C., Plattner C., Laschober G., Rieder D., Hackl H., Krogsdam A., Loncova Z., Posch W., Wilflingseder D., Sopper S., Ijsselsteijn M., Brouwer T. P., Johnson D., Xu Y., Wang Y., Sanders M. E., Estrada M. V., Ericsson-Gonzalez P., Charoentong P., Balko J., De Miranda N. F. D. C. C., Trajanoski Z. (2019). Molecular and Pharmacological Modulators of the Tumor
Immune Contexture Revealed by Deconvolution of RNA-Seq Data. Genome Med..

[18] Baeten C. I. M., Wagstaff J., Verhoeven I. C. L., Hillen H. F. P., Griffioen A. W. (2002). Flow Cytometric
Quantification of Tumour Endothelial Cells; an Objective Alternative
for Microvessel Density Assessment. Br. J. Cancer.

[19] ProteoWizard : Home. https://proteowizard.sourceforge.io/ (accessed 2025–06–02).

[20] Bemis K. D., Harry A., Eberlin L. S., Ferreira C., Van De Ven S. M., Mallick P., Stolowitz M., Vitek O. (2015). Cardinal: An R Package
for Statistical Analysis of Mass Spectrometry-Based Imaging Experiments. Bioinformatics.

[21] Cardinal 3 : User Guide for Mass Spectrometry Imaging Analysis. https://www.bioconductor.org/packages/release/bioc/vignettes/Cardinal/inst/doc/Cardinal3-guide.html (accessed 2025–06–02).

[22] Edney M. K., Kotowska A. M., Spanu M., Trindade G. F., Wilmot E., Reid J., Barker J., Aylott J. W., Shard A. G., Alexander M. R., Snape C. E., Scurr D. J. (2022). Molecular Formula
Prediction for Chemical Filtering of 3D OrbiSIMS Datasets. Anal. Chem..

[23] Pavlova N. N., Thompson C. B. (2016). The Emerging Hallmarks
of Cancer Metabolism. Cell Metab..

[24] Quinones A., Le A. (2021). The Multifaceted Glioblastoma:
From Genomic Alterations to Metabolic
Adaptations. Adv. Exp. Med. Biol..

[25] Keenan M. R., Trindade G. F., Pirkl A., Newell C. L., Jin Y., Aizikov K., Dannhorn A., Zhang J., Matjačić L., Arlinghaus H., Eyres A., Havelund R., Goodwin R. J. A., Takats Z., Bunch J., Gould A. P., Makarov A., Gilmore I. S. (2025). Orbitrap Noise Structure and Method for Noise Unbiased
Multivariate Analysis. Nat. Commun..

[26] Ntshangase, S. ; Khan, S. ; Bezuidenhout, L. ; Gazárková, T. ; Kaczynski, J. ; Sellers, S. ; Rattray, N. J. ; Newby, D. E. ; Hadoke, P. W. ; Andrew, R. Spatial Lipidomic Profiles of Atherosclerotic Plaques: A Mass Spectrometry Imaging Study, 2025, 282:126954 10.1016/j.talanta.2024.126954. 39423636

[27] Njoku K., Campbell A. E., Geary B., MacKintosh M. L., Derbyshire A. E., Kitson S. J., Sivalingam V. N., Pierce A., Whetton A. D., Crosbie E. J. (2021). Metabolomic Biomarkers
for the Detection of Obesity-Driven Endometrial Cancer. Cancers.

[28] Crowley G., Kwon S., Haider S. H., Caraher E. J., Lam R., St-Jules D. E., Liu M., Prezant D. J., Nolan A. (2018). Metabolomics
of World Trade Center-Lung Injury: A Machine Learning Approach. BMJ. Open Respir. Res..

[29] Pang Z., Lu Y., Zhou G., Hui F., Xu L., Viau C., Spigelman A. F., Macdonald P. E., Wishart D. S., Li S., Xia J. (2024). MetaboAnalyst 6.0:
Towards a Unified Platform for Metabolomics Data
Processing, Analysis and Interpretation. Nucleic
Acids Res..

[30] Dannhorn A., Swales J. G., Hamm G., Strittmatter N., Kudo H., Maglennon G., Goodwin R. J. A., Takats Z. (2022). Evaluation
of Formalin-Fixed and FFPE Tissues for Spatially Resolved Metabolomics
and Drug Distribution Studies. Pharmaceuticals.

[31] Saharuka, V. ; Vieira, L. M. ; Stuart, L. ; Ekelöf, M. ; Molenaar, M. R. ; Bailoni, A. ; Ovchinnikova, K. ; Soltwisch, J. ; Bausbacher, T. ; Jakob, D. ; King, M. ; Müller, M. A. ; Oetjen, J. ; Pace, C. ; Pinto, F. E. ; Strittmatter, N. ; Velickovic, D. ; Spengler, B. ; Muddiman, D. C. ; Liebeke, M. ; Janfelt, C. ; Goodwin, R. ; Eberlin, L. S. ; Anderton, C. R. ; Hopf, C. ; Dreisewerd, K. ; Alexandrov, T. Large-Scale Evaluation of Spatial Metabolomics Protocols and Technologies. bioRxiv 2024, 2024. 10.1101/2024.01.29.577354.

[32] Schymanski E. L., Jeon J., Gulde R., Fenner K., Ruff M., Singer H. P., Hollender J. (2014). Identifying
Small Molecules via High
Resolution Mass Spectrometry: Communicating Confidence. Environ. Sci. Technol..

[33] Zhou W., Yao Y., Scott A. J., Wilder-Romans K., Dresser J. J., Werner C. K., Sun H., Pratt D., Sajjakulnukit P., Zhao S. G., Davis M., Nelson B. S., Halbrook C. J., Zhang L., Gatto F., Umemura Y., Walker A. K., Kachman M., Sarkaria J. N., Xiong J., Morgan M. A., Rehemtualla A., Castro M. G., Lowenstein P., Chandrasekaran S., Lawrence T. S., Lyssiotis C. A., Wahl D. R. (2020). Purine Metabolism
Regulates DNA Repair and Therapy Resistance in Glioblastoma. Nat. Commun..

[34] Badr C. E., Silver D. J., Siebzehnrubl F. A., Deleyrolle L. P. (2020). Metabolic
Heterogeneity and Adaptability in Brain Tumors. Cell. Mol. Life Sci..

[35] Seliger C., Meyer A. L., Leidgens V., Rauer L., Moeckel S., Jachnik B., Proske J., Dettmer K., Rothhammer-Hampl T., Kaulen L. D., Riemenschneider M. J., Oefner P. J., Kreutz M., Schmidt N. O., Merrill M., Uhl M., Renner K., Vollmann-Zwerenz A., Proescholdt M., Hau P. (2022). Metabolic Heterogeneity
of Brain Tumor Cells of Proneural and Mesenchymal Origin. Int. J. Mol. Sci..

[36] Le
Thuc O., Gruber T., Tschöp M. H., García-Cáceres C. (2021). Control of
Systemic Metabolism by Astrocytes in the Brain. Masterclass in Neuroendocrinology.

[37] Saraswat M., Rueda-Gensini L., Heinzelmann E., Gracia T., Memi F., De Jong G., Straub J., Schloo C., Hoffmann D. C., Jung E., Kindinger T., Weigel B., Lim B., Weil S., Gould O., Mair R., Mikulik K., Rohbeck M., Wick W., Winkler F., Bayraktar O. A., Stegle O., Mall M. (2025). Decoding Plasticity Regulators and
Transition Trajectories in Glioblastoma with Single-Cell Multiomics. bioRxiv.

[38] Darmanis S., Sloan S. A., Croote D., Mignardi M., Chernikova S., Samghababi P., Zhang Y., Neff N., Kowarsky M., Caneda C., Li G., Chang S. D., Connolly I. D., Li Y., Barres B. A., Gephart M. H., Quake S. R. (2017). Single-Cell RNA-Seq
Analysis of Infiltrating Neoplastic Cells at the Migrating Front of
Human Glioblastoma. Cell Rep..

[39] Xia P., Logiacco F., Huang Y., Kettenmann H., Semtner M. (2021). Histamine Triggers Microglial Responses
Indirectly
via Astrocytes and Purinergic Signaling. Glia.

[40] Dietschy J. M., Turley S. D. (2004). Thematic Review
Series: Brain Lipids. Cholesterol Metabolism
in the Central Nervous System during Early Development and in the
Mature Animal. J. Lipid Res..

[41] Zhou B., Zuo Y. X., Jiang R. T. (2019). Astrocyte
Morphology: Diversity,
Plasticity, and Role in Neurological Diseases. CNS Neurosci. Ther..

[42] Vasile F., Dossi E., Rouach N. (2017). Human Astrocytes: Structure and Functions
in the Healthy Brain. Brain Struct. Funct..

[43] Sofi, R. R. In Search for New Therapeutic Approaches for Glioblastoma Multiforme; University of Bristol. https://research-information.bris.ac.uk/en/studentTheses/in-search-for-new-therapeutic-approaches-for-glioblastoma-multifo (accessed 2025–06–02).

[44] Greer T., Sturm R., Li L. (2011). Mass Spectrometry Imaging for Drugs
and Metabolites. J. Proteomics.

[45] Figlia G., Willnow P., Teleman A. A. (2020). Metabolites Regulate
Cell Signaling
and Growth via Covalent Modification of Proteins. Dev. Cell.

[46] Watson D. C., Bayik D., Storevik S., Moreino S. S., Sprowls S. A., Han J., Augustsson M. T., Lauko A., Sravya P., Røsland G. V., Troike K., Tronstad K. J., Wang S., Sarnow K., Kay K., Lunavat T. R., Silver D. J., Dayal S., Joseph J. V., Mulkearns-Hubert E., Ystaas L. A. R., Deshpande G., Guyon J., Zhou Y., Magaut C. R., Seder J., Neises L., Williford S. E., Meiser J., Scott A. J., Sajjakulnukit P., Mears J. A., Bjerkvig R., Chakraborty A., Daubon T., Cheng F., Lyssiotis C. A., Wahl D. R., Hjelmeland A. B., Hossain J. A., Miletic H., Lathia J. D. (2023). GAP43-Dependent Mitochondria Transfer from Astrocytes
Enhances Glioblastoma Tumorigenicity. Nat. Cancer.

[47] Wu J., Li R., Wang J., Zhu H., Ma Y., You C., Shu K. (2025). Reactive Astrocytes
in Glioma: Emerging Opportunities and Challenges. Int. J. Mol. Sci..

[48] He W., Edney M. K., Paine S. M. L., Griffiths R. L., Scurr D. J., Rahman R., Kim D.-H. (2023). Untargeted
Metabolomic
Characterization of Glioblastoma Intra-Tumor Heterogeneity Using OrbiSIMS. Anal. Chem..

[49] Samaržija I., Trošelj K. G., Konjevoda P. (2023). Prognostic Significance of Amino
Acid Metabolism-Related Genes in Prostate Cancer Retrieved by Machine
Learning. Cancers (Basel).

[50] Ogretmen B. (2018). Sphingolipid
Metabolism in Cancer Signalling and Therapy. Nat. Rev. Cancer.

[51] Henrik
Heiland D., Ravi V. M., Behringer S. P., Frenking J. H., Wurm J., Joseph K., Garrelfs N. W. C., Strähle J., Heynckes S., Grauvogel J., Franco P., Mader I., Schneider M., Potthoff A. L., Delev D., Hofmann U. G., Fung C., Beck J., Sankowski R., Prinz M., Schnell O. (2019). Tumor-Associated
Reactive Astrocytes Aid the Evolution of Immunosuppressive Environment
in Glioblastoma. Nat. Commun..

[52] Gagliano N., Costa F., Cossetti C., Pettinari L., Bassi R., Chiriva-Internati M., Cobos E., Gioia M., Pluchino S. (2009). Glioma-Astrocyte Interaction
Modifies the Astrocyte
Phenotype in a Co-Culture Experimental Model. Oncol. Rep..

[53] Zhou Y., Zhu Y., Wu Y., Xiang X., Ouyang X., Liu L., Li T. (2024). 4-Phenylbutyric Acid Improves Sepsis-Induced Cardiac Dysfunction
by Modulating Amino Acid Metabolism and Lipid Metabolism via Comt/Ptgs2/Ppara. Metabolomics.

[54] Vera-Aviles M., Vantana E., Kardinasari E., Koh N. L., Latunde-Dada G. O. (2018). Protective
Role of Histidine Supplementation Against Oxidative Stress Damage
in the Management of Anemia of Chronic Kidney Disease. Pharmaceuticals.

[55] Huang M., Long A., Hao L., Shi Z., Zhang M. (2025). Astrocyte
in Neurological Disease: Pathogenesis and Therapy. MedComm (Beijing)..

[56] Hawkins C. C., Ali T., Ramanadham S., Hjelmeland A. B. (2020). Sphingolipid Metabolism in Glioblastoma
and Metastatic Brain Tumors: A Review of Sphingomyelinases and Sphingosine-1-Phosphate. Biomolecules.

[57] Yamashita D., Bernstock J. D., Elsayed G., Sadahiro H., Mohyeldin A., Chagoya G., Ilyas A., Mooney J., Estevez-Ordonez D., Yamaguchi S., Flanary V. L., Hackney J. R., Bhat K. P., Kornblum H. I., Zamboni N., Kim S. H., Chiocca E. A., Nakano I. (2021). Targeting Glioma-Initiating Cells via the Tyrosine
Metabolic Pathway. J. Neurosurg..

