# Supplementary material for: Single-Cell Metabolic Profiling in a Glioblastoma Coculture Model Using AP-MALDI-Based Mass Spectrometry Imaging
Source: Anal Chem. 2026 Apr 7;98(15):11246–60. doi: 10.1021/acs.analchem.5c07924 (PMC13103930; doi:10.1021/acs.analchem.5c07924)
Supplement: Supplementary file 1 [file ac5c07924_si_001.pdf]

Supporting information

Single-Cell Metabolic Profiling in a Glioblastoma Co-culture Model Using AP-MALDI-based Mass Spectrometry Imaging

Une Kontrimaite*[1], Kei F. Carver Wong[2], Sandra Martinez Jarquin[2], Phoebe McCrorie[1], Ruman Rahman[1], Dong-Hyun Kim*[2,3]

[1] Biodiscovery Institute, School of Medicine, University of Nottingham, NG7 2RD

[2] Centre for Analytical Bioscience, Advanced Materials & Health Technologies Division, School of Pharmacy, University of Nottingham, NG7 2RD

[3] College of Pharmacy, Kyungpook National University, Daegu 41566, Republic of Korea

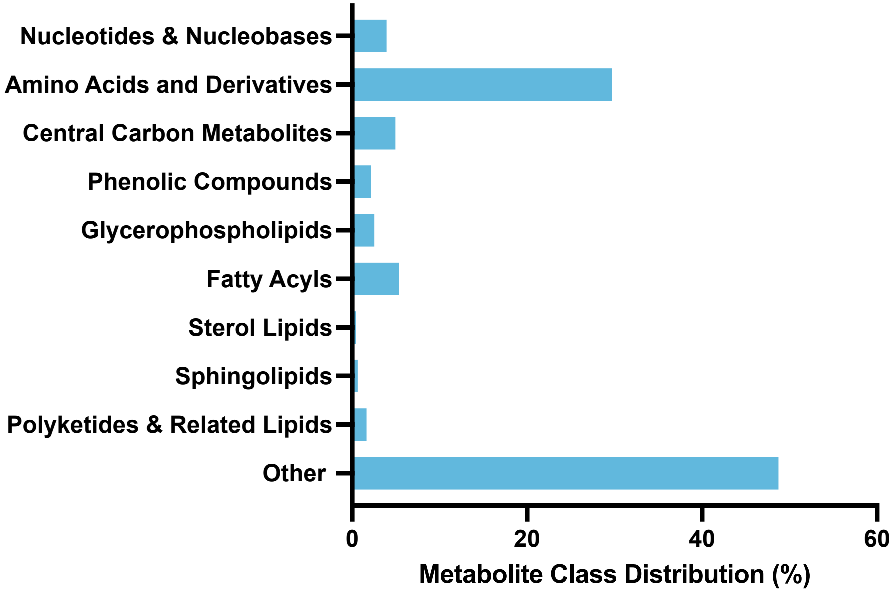

Figure S1. Class distribution of LC-MS-identified metabolites. Bar chart showing the percentage distribution of identified metabolites across major chemical classes detected by LC-MS analysis of $5x10^5$ cells. Classes include Nucleotides & Nucleobases, Amino Acids and Derivatives, Central Carbon Metabolites, Phenolic Compounds, Glycerophospholipids, Fatty Acyls, Sterol Lipids, Sphingolipids, and Polyketides & Related Lipids. The "Other" category contains metabolites that could not be confidently assigned to a specific class due to structural diversity or limited annotation. Percentages were calculated as the proportion of each class relative to the total number of LC-MS-identified metabolites.

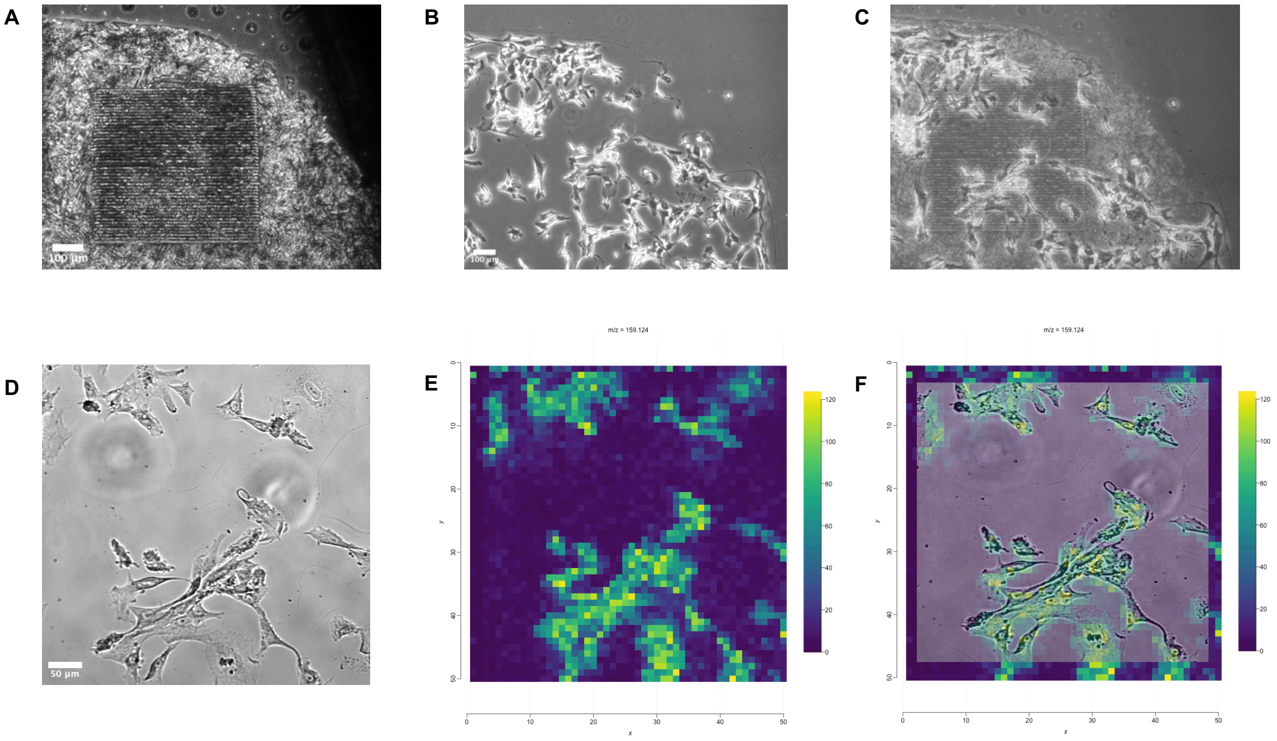

Figure S2. Spatial alignment of microscopy and AP-MALDI-MSI images and identification of single-cell regions of interest (ROIs). (A) Low-magnification (×4) brightfield microscopy image used for initial identification of the region of interest (ROI) prior to AP-MALDI imaging. (B) Brightfield image of the same region following AP-MALDI acquisition, showing the laser ablation raster pattern. (C) Overlay of pre- and post-ablation brightfield images to define the ablated region and confirm spatial correspondence. (D) Higher-magnification (×10) images of the selected region used to refine spatial alignment by matching individual cells and local morphological features. (E) Overlay of the AP-MALDI-MSI ion image with the corresponding microscopy image, demonstrating accurate spatial correspondence between MSI pixels and cellular morphology.

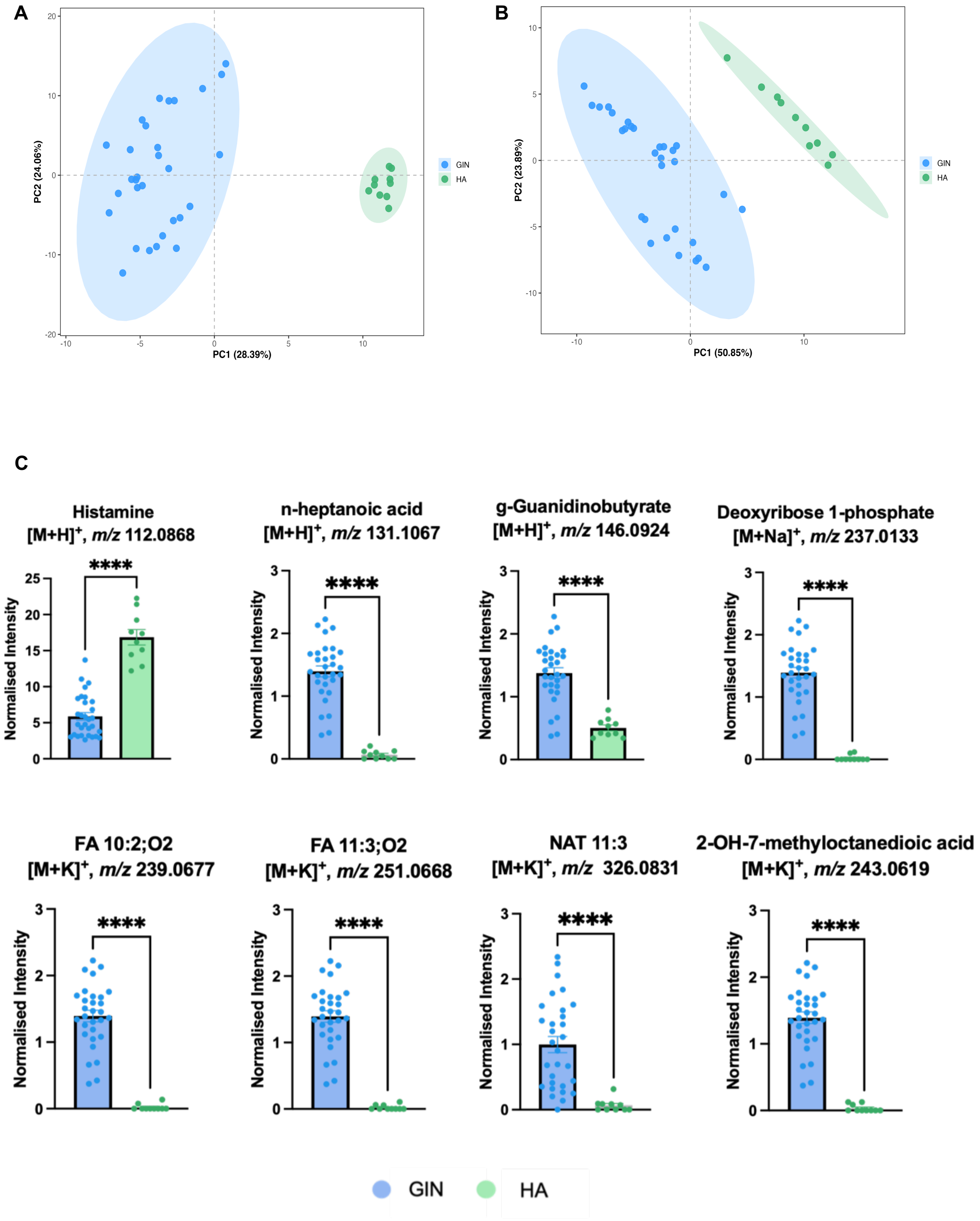

Figure S3. Comparative metabolomic analysis of HA and GIN single cells. PCA plot of HA and GIN single cells acquired in **(A)** positive ion mode and **(B)** negative ion mode. **(C)** Statistical comparison of metabolite profiles between GIN and HA cells. Significantly different metabolites were identified using univariate statistical analysis ($p < 0.05$) and Random Forest classification to assess feature importance.

Metabolite	*HMDB Identifier*	*Formula*	*Adduct*	*Observed m/z*	*Theoretical m/z*	*Mass Error (ppm)*	*LC-MS confirmed*	*LC-MS RT (min)*	*Identification Level*
3-[(4-Acetamidobutyl)amino]propylacetamide	No ID match	$C_{11}H_{24}N_3O_2$	$[M+H]^+$	230.1868	230.1869	2.16	Yes	15.03	Level 2
Salsoline	HMDB0012469	$C_{11}H_{16}NO_2$	$[M+H]^+$	194.1177	194.1181	0.75	No	-	Level 3
Metanephrine	HMDB0004063	$C_{10}H_{15}NO_2K$	$[M+K]^+$	236.0685	236.0684	0.63	No	-	Level 3
Metanephrine	HMDB0004063	$C_{10}H_{19}N_2O_3$	$[M+NH_4]^+$	215.1394	215.1396	1.77	No	-	Level 3
1,2,3-Heptanetriol	No ID match	$C_7H_{16}O_3Na$	$[M+Na]^+$	171.0992	171.0992	0.2	No	-	Level 3
1,2,3-Heptanetriol	No ID match	$C_7H_{17}O_3$	$[M+H]^+$	149.1171	149.1178	-0.81	No	-	Level 3
2,5-Dioxo-3-hexenal	No ID match	$C_6H_7O_3$	$[M+H]^+$	127.039	127.0395	0.23	No	-	Level 3
9-Octadecenamide	HMDB0002114	$C_{18}H_{36}NO$	$[M+H]^+$	282.2801	282.2797	3.4	No	-	Level 3
3-O-Methyldopa	HMDB0060747	$C_{10}H_{14}NO_4$	$[M+H]^+$	212.0919	212.0923	0.78	No	-	Level 3
Non-2-enal	HMDB0032107	$C_9H_{20}NO$	$[M+NH_4]^+$	158.154	158.1545	0.38	No	-	Level 3
1,3-Octadiene	No ID match	C_8H_{13}	$[M+H]^+$	109.1012	109.1017	0.22	No	-	Level 3
1,3-Octadiene	No ID match	$C_8H_{16}N$	$[M+NH_4]^+$	126.1278	126.1283	0.59	No	-	Level 3
1-Aminocyclopropane-1-carboxylate	HMDB0004403	$C_4H_8NO_2$	$[M+H]^+$	102.055	102.0555	0.44	No	-	Level 3
1-nonanoic acid	HMDB0000847	$C_9H_{17}O_2$	$[M-H]^-$	157.1234	157.1229	-0.02	No	-	Level 3
1-pyrroline-5-carboxylic acid	HMDB0001301	$C_5H_7NO_2K$	$[M+K]^+$	152.0103	152.0108	-3.53	No	-	Level 3
1-pyrroline-5-carboxylic acid	HMDB0001301	$C_5H_8NO_2$	$[M+H]^+$	114.055	114.0555	0.39	No	-	Level 3
1,3-dimethyluracil	HMDB0002144	$C_6H_9N_2O_2$	$[M+H]^+$	141.0659	141.0664	0.33	No	-	Level 3
1,4-Bipiperidine-1-carboxylic acid	No ID match	$C_{11}H_{21}N_2O_2$	$[M+H]^+$	213.16	213.1603	1.15	No	-	Level 3
12-Aminododecanamide	No ID match	$C_{13}H_{24}O_4K$	$[M+K]^+$	253.1667	253.1677	-3.83	No	-	Level 3
1H-Imidazole-4-ethanamine	HMDB0000870	$C_5H_{10}N_3$	$[M+H]^+$	112.0869	112.0875	-0.21	No	-	Level 3
2-(Diethylamino)ethanol	No ID match	$C_6H_{16}NO$	$[M+H]^+$	118.1227	118.1232	0.51	No	-	Level 3
2-Acetamidohexanedioic acid	No ID match	$C_8H_{14}NO_5$	$[M+H]^+$	204.0869	204.0872	1.22	No	-	Level 3
2-Amino-3-(1H-imidazol-4-yl)propanal	No ID match	$C_6H_{10}N_3O$	$[M+H]^+$	140.0819	140.0824	0.44	No	-	Level 3
2-Amino-3-(1H-imidazol-4-yl)propanal	No ID match	$C_6H_{13}N_4O$	$[M+NH_4]^+$	157.1085	157.1089	0.72	No	-	Level 3

2-Amino-4-methylpyrimidine	No ID match	$C_5H_{11}N_4$	$[M+NH_4]^+$	127.0979	127.0984	0.61	No	-	Level 3
2-Amino-4-methylpyrimidine	No ID match	$C_5H_8N_3$	$[M+H]^+$	110.0713	110.0718	0.24	Yes	13.39	Level 2
2-Aminomuconic acid semialdehyde	No ID match	$C_6H_{11}N_2O_3$	$[M+NH_4]^+$	159.0765	159.077	0.51	No	-	Level 3
2-Chromanone	No ID match	$C_9H_9O_2$	$[M+H]^+$	149.0597	149.0603	-0.04	No	-	Level 3
2-Deoxycytidine	HMDB0000014	$C_9H_{13}N_3O_4Na$	$[M+Na]^+$	250.0799	250.0798	0.29	No	-	Level 3
2-Furoic acid	HMDB0000617	$C_5H_3O_3$	$[M-H]^-$	111.0088	111.0082	0.28	Yes	14.76	Level 2
2-Methylfuran	No ID match	$C_5H_{10}NO$	$[M+NH_4]^+$	100.0757	100.0762	0.1	No	-	Level 3
2-Methylfuran	No ID match	C_5H_7O	$[M+H]^+$	83.0491	83.0497	-0.49	No	-	Level 3
2-pyridone	No ID match	C_5H_6NO	$[M+H]^+$	96.0444	96.0449	0.1	No	-	Level 3
2-pyridone	No ID match	$C_5H_9N_2O$	$[M+NH_4]^+$	113.071	113.0715	0.54	No	-	Level 3
2-Pyrrolidone	HMDB0002039	$C_4H_{11}N_2O$	$[M+NH_4]^+$	103.0866	103.0871	0.11	No	-	Level 3
2-Pyrrolidone	HMDB0002039	C_4H_7NOK	$[M+K]^+$	124.0156	124.0159	-2.59	No	-	Level 3
2-Pyrrolidone	HMDB0002039	C_4H_8NO	$[M+H]^+$	86.0601	86.0606	0.7	No	-	Level 3
3-(4-Hydroxy-3-methoxyphenyl)-2-oxiranecarboxylic acid	No ID match	$C_{10}H_9O_5$	$[M-H]^-$	209.0457	209.045	0.72	No	-	Level 3
3-(4-Hydroxyphenyl)pyruvate	HMDB0000707	$C_9H_{12}NO_4$	$[M+NH_4]^+$	198.0762	198.0766	0.58	No	-	Level 3
3-(4-Hydroxyphenyl)pyruvate	HMDB0000707	$C_9H_7O_4$	$[M-H]^-$	179.0351	179.0344	0.65	No	-	Level 3
3-(4-Hydroxyphenyl)pyruvate	HMDB0000707	$C_9H_9O_4$	$[M+H]^+$	181.0496	181.0501	0.35	No	-	Level 3
3-(5-methyl-2-furyl)prop-2-enal	No ID match	$C_8H_7O_2$	$[M-H]^-$	135.0453	135.0446	1.08	No	-	Level 3
3-(5-methyl-2-furyl)prop-2-enal	No ID match	$C_8H_9O_2$	$[M+H]^+$	137.0598	137.0603	0.69	No	-	Level 3
3-Amino-2-piperidinone	No ID match	$C_5H_{10}N_2OK$	$[M+K]^+$	153.0419	153.0425	-3.73	No	-	Level 3
3-Amino-2-piperidinone	No ID match	$C_5H_{11}N_2O$	$[M+H]^+$	115.0866	115.0871	0.09	No	-	Level 3
3-Butenoic acid	No ID match	$C_4H_5O_2$	$[M-H]^-$	85.0295	85.029	-0.05	No	-	Level 3
3-Butenoic acid	No ID match	$C_4H_7O_2$	$[M+H]^+$	87.0441	87.0446	0.5	No	-	Level 3
3-Hydroxy-2H-indole-5,6(3H,7H)-dione	No ID match	$C_8H_8NO_3$	$[M+H]^+$	166.05	166.0504	0.78	No	-	Level 3
3-hydroxy-N-(2-oxotetrahydrofuranyl)pentanamide	No ID match	$C_9H_{16}NO_4$	$[M+H]^+$	202.1075	202.1079	0.57	No	-	Level 3
3-Hydroxyphenylacetate	HMDB0000440	$C_8H_7O_3$	$[M-H]^-$	151.0402	151.0395	0.87	No	-	Level 3

3-Methoxy-4-hydroxyhippuric acid	No ID match	$C_{10}H_{12}NO_5$	$[M+H]^+$	226.0714	226.0715	1.77	No	-	Level 3
3-Methoxy-4-Hydroxyphenylglycolsulfate	No ID match	$C_9H_{12}O_7SNa$	$[M+Na]^+$	287.0195	287.0196	-0.33	No	-	Level 3
3-Methoxytyramine	HMDB0000022	$C_9H_{14}NO_2$	$[M+H]^+$	168.102	168.1025	0.56	No	-	Level 3
3-Methoxytyramine	HMDB0000022	$C_9H_{17}N_2O_2$	$[M+NH_4]^+$	185.1286	185.129	0.79	No	-	Level 3
3-Methyl-2-oxobutanoic acid	No ID match	$C_5H_7O_3$	$[M-H]^-$	115.0401	115.0395	0.27	No	-	Level 3
3-Methylbutanoic acid	HMDB0255021	$C_5H_9O_2$	$[M-H]^-$	101.0608	101.0603	-0.04	No	-	Level 3
3-Oxo-4-pentenoic acid	No ID match	$C_5H_5O_3$	$[M-H]^-$	113.0245	113.0239	0.72	No	-	Level 3
-Oxo-N-(2-oxotetrahydro-3-furanyl)heptanamide	No ID match	$C_{10}H_{17}N_3O_6KS$	$[M+K]^+$	266.0794	266.0789	1.82	No	-	Level 3
-Oxo-N-(2-oxotetrahydro-3-furanyl)heptanamide	No ID match	$C_{11}H_{18}NO_4$	$[M+H]^+$	228.1236	228.1236	2.48	No	-	Level 3
3-Phenylpropanal	HMDB0033716	$C_9H_{14}NO$	$[M+NH_4]^+$	152.1069	152.1075	-0.59	No	-	Level 3
3,4-Dihydroxyphenylacetate	HMDB0001336	$C_8H_{12}NO_4$	$[M+NH_4]^+$	186.0762	186.0766	0.62	No	-	Level 3
3,4-Dihydroxyphenylacetate	HMDB0001336	$C_8H_9O_4$	$[M+H]^+$	169.0496	169.0501	0.38	No	-	Level 3
3,4-Dimethoxyphenethylamine	No ID match	$C_{10}H_{16}NO_2$	$[M+H]^+$	182.1177	182.1181	0.8	No	-	Level 3
3,4-Dimethoxyphenethylamine	No ID match	$C_{10}H_{19}N_2O_2$	$[M+NH_4]^+$	199.1442	199.1447	0.48	No	-	Level 3
3,4-Dimethoxyphenethylamine	No ID match	$C_7H_{15}NO_4K$	$[M+K]^+$	220.0734	220.0734	-0.16	No	-	Level 3
4-(2-Aminophenyl)-2,4-dioxobutanoic acid	No ID match	$C_{10}H_{10}NO_4$	$[M+H]^+$	208.0607	208.061	1.27	No	-	Level 3
4-Aminobenzoate	HMDB0304171	$C_7H_{11}N_2O_2$	$[M+NH_4]^+$	155.0814	155.0821	-0.67	No	-	Level 3
4-Aminobenzoate	HMDB0304171	$C_7H_8NO_2$	$[M+H]^+$	138.055	138.0555	0.33	No	-	Level 3
4-Aminobutanoate	HMDB0000112	$C_4H_{10}NO_2$	$[M+H]^+$	104.0706	104.0712	-0.05	No	-	Level 3
4-Aminophenol	No ID match	$C_6H_{11}N_2O$	$[M+NH_4]^+$	127.0866	127.0871	0.09	No	-	Level 3
4-Hydroxybenzoic acid	HMDB0000500	$C_7H_5O_3$	$[M-H]^-$	137.0245	137.0239	0.59	Yes	4.78	Level 2
4-Hydroxybenzoic acid	HMDB0000500	$C_7H_7O_3$	$[M+H]^+$	139.039	139.0395	0.21	No	-	Level 3
4-Nitrophenol	No ID match	$C_6H_4NO_3$	$[M-H]^-$	138.0198	138.0191	0.96	No	-	Level 3
5-Aminolevulinate	HMDB0001149	$C_5H_{10}NO_3$	$[M+H]^+$	132.0656	132.0661	0.61	No	-	Level 3
5-Hydroxy-1,5-dihydro-2H-pyrrol-2-one	No ID match	$C_4H_6NO_2$	$[M+H]^+$	100.0393	100.0399	-0.05	No	-	Level 3
5-Hydroxytryptophol	No ID match	$C_{10}H_{11}NOK$	$[M+K]^+$	216.0422	216.0421	0.3	No	-	Level 3

5-Hydroxytryptophol	No ID match	$C_{10}H_{12}NO_2$	$[M+H]^+$	178.0864	178.0868	0.81	No	-	Level 3
5-Hydroxytryptophol	No ID match	$C_{10}H_{15}N_2O_2$	$[M+NH_4]^+$	195.1129	195.1134	0.49	No	-	Level 3
5-Methoxy-3-indoleaceate	No ID match	$C_{11}H_{11}NO_2K$	$[M+K]^+$	244.0375	244.0371	1.84	No	-	Level 3
5-Methoxy-3-indoleaceate	No ID match	$C_{11}H_{12}NO_3$	$[M+H]^+$	206.0814	206.0817	1.12	No	-	Level 3
5-Methoxytryptamine	HMDB0004095	$C_{11}H_{18}N_3O$	$[M+NH_4]^+$	208.1447	208.145	1.26	No	-	Level 3
5-Nitrilonorvaline	No ID match	$C_5H_8N_2O_2Na$	$[M+Na]^+$	151.0477	151.0478	-0.65	No	-	Level 3
5-Nitrilonorvaline	HMDB0000267	$C_5H_9N_2O_2$	$[M+H]^+$	129.0659	129.0664	0.36	No	-	Level 3
5-Oxoproline	HMDB0000267	$C_5H_7NO_3Na$	$[M+Na]^+$	152.0317	152.0318	-0.75	No	-	Level 3
5-Oxoproline	HMDB0000267	$C_5H_8NO_3$	$[M+H]^+$	130.05	130.0504	1	No	-	Level 3
6-Oxo-pipecolinic acid	HMDB0000716	$C_6H_{10}NO_3$	$[M+H]^+$	144.0655	144.0661	-0.14	No	-	Level 3
6-Oxohexanoic acid	HMDB0032921	$C_6H_9O_3$	$[M-H]^-$	129.0558	129.0552	0.63	No	-	Level 3
9-Aminononanoic acid	HMDB0033039	$C_9H_{20}NO_2$	$[M+H]^+$	174.149	174.1494	0.83	No	-	Level 3
Acetoin	HMDB0001694	$C_4H_7O_2$	$[M-H]^-$	87.0452	87.0446	0.53	No	-	Level 3
Acetylcadaverine	HMDB0033474	$C_7H_{17}N_2O$	$[M+H]^+$	145.1335	145.1341	-0.27	No	-	Level 3
Adenine	HMDB0000034	$C_5H_6N_5$	$[M+H]^+$	136.0618	136.0623	0.21	Yes	11.99	Level 2
Ribose 1,5-bisphosphate	HMDB0001341	$C_5H_{16}NO_{11}P_2$	$[M+NH_4]^+$	328.0196	328.0199	0.88	No	-	Level 3
Aminoproline	No ID match	$C_5H_{10}N_2O_2K$	$[M+K]^+$	169.0371	169.0374	-1.69	No	-	Level 3
Aminoproline	No ID match	$C_5H_{11}N_2O_2$	$[M+H]^+$	131.0816	131.0821	0.73	No	-	Level 3
Adenosine Monophosphate	HMDB0000045	$C_{10}H_{15}N_5O_7P$	$[M+H]^+$	348.071	348.0709	1.83	No	-	Level 3
Aspartate	HMDB0000191	$C_4H_8NO_4$	$[M+H]^+$	134.0448	134.0453	0.11	No	-	Level 3
Ala-Lys	HMDB0033077	$C_9H_{20}N_3O_3$	$[M+H]^+$	218.1503	218.1505	1.75	Yes	13.45	Level 2
Benzaldehyde	HMDB0000527	$C_7H_{10}NO$	$[M+NH_4]^+$	124.0758	124.0762	0.89	No	-	Level 3
Benzaldehyde	HMDB0000527	C_7H_7O	$[M+H]^+$	107.0492	107.0497	0.55	No	-	Level 3
Benzoate	HMDB0001877	$C_7H_{10}NO_2$	$[M+NH_4]^+$	140.0707	140.0712	0.68	No	-	Level 3
Benzoate	HMDB0001877	$C_7H_5O_2$	$[M-H]^-$	121.0296	121.029	0.79	No	-	Level 3

Benzoate	HMDB0001877	$C_7H_7O_2$	$[M+H]^+$	123.0441	123.0446	0.36	No	-	Level 3
benzoquinone	HMDB0033499	$C_6H_5O_2$	$[M+H]^+$	109.0285	109.029	0.86	No	-	Level 3
benzoquinone	HMDB0033499	$C_6H_8NO_2$	$[M+NH_4]^+$	126.055	126.0555	0.36	No	-	Level 3
benzoquinoneacetic acid	No ID match	$C_8H_7O_4$	$[M+H]^+$	167.034	167.0344	0.68	No	-	Level 3
Benzylamine	HMDB0000877	$C_7H_{10}N$	$[M+H]^+$	108.0808	108.0813	0.23	No	-	Level 3
Cytidine diphosphate	HMDB0000141	$C_9H_{19}N_4O_{11}P_2$	$[M+NH_4]^+$	421.0533	421.0526	3.07	No	-	Level 3
Choline	HMDB0000097	$C_5H_{14}NO$	$[M+H]^+$	104.107	104.1075	0.1	Yes	12.31	Level 2
Choline phosphate	HMDB0001565	$C_5H_{15}NO_4P$	$[M+H]^+$	184.0734	184.0739	0.43	No	-	Level 3
Citrate	HMDB0000094	$C_6H_7O_7$	$[M-H]^-$	191.0199	191.0192	0.9	No	-	Level 3
Cotinine N-oxide	HMDB0001048	$C_{10}H_{13}N_2O_2$	$[M+H]^+$	193.0973	193.0977	0.76	No	-	Level 3
Creatine	HMDB0000064	$C_4H_{10}N_3O_2$	$[M+H]^+$	132.0768	132.0773	0.36	Yes	12.82	Level 2
Creatinine	HMDB0000562	$C_4H_8N_3O$	$[M+H]^+$	114.0662	114.0667	0.1	Yes	11.03	Level 2
Cyclo(leucylprolyl)	HMDB0033437	$C_{11}H_{19}N_2O_2$	$[M+H]^+$	211.1443	211.1447	0.93	No	-	Level 3
Cyclo(leucylprolyl)	HMDB0033437	$C_{11}H_{22}N_3O_2$	$[M+NH_4]^+$	228.1713	228.1712	2.84	No	-	Level 3
Cyclohexane	No ID match	$C_6H_{16}N$	$[M+NH_4]^+$	102.1278	102.1283	0.73	No	-	Level 3
Cyclohexanecarboxylic acid	HMDB0033541	$C_7H_{16}NO_2$	$[M+NH_4]^+$	146.1175	146.1181	-0.38	No	-	Level 3
Cyclopentanone	No ID match	$C_5H_{12}NO$	$[M+NH_4]^+$	102.0914	102.0919	0.59	No	-	Level 3
Cytidine	HMDB0000089	$C_9H_{13}N_3O_5Na$	$[M+Na]^+$	266.0751	266.0747	1.35	No	-	Level 3
Cytosine	HMDB0000630	$C_4H_6N_3O$	$[M+H]^+$	112.0506	112.0511	0.55	Yes	11.3	Level 2
1-Piperideine-2-carboxylic acid	HMDB0000715	$C_6H_{10}NO_2$	$[M+H]^+$	128.0706	128.0712	-0.04	No	-	Level 3
Erythrose	HMDB0000270	$C_4H_8O_4K$	$[M+K]^+$	159.0054	159.0054	-0.11	No	-	Level 3
Homoproline	No ID match	$C_5H_{11}NO_2K$	$[M+K]^+$	168.0418	168.0421	-2	No	-	Level 3
Homoproline	No ID match	$C_6H_{12}NO_2$	$[M+H]^+$	130.0863	130.0868	0.35	No	-	Level 3
Decanoic acid	HMDB0000511	$C_{10}H_{19}O_2$	$[M-H]^-$	171.1391	171.1385	0.27	No	-	Level 3
dehydroalanine	No ID match	$C_3H_6NO_2$	$[M+H]^+$	88.0393	88.0399	-0.06	No	-	Level 3

Deoxyguanosine	HMDB0000085	$C_{10}H_{14}N_5O_4$	$[M+H]^+$	268.1051	268.1046	3.99	No	-	Level 3
Dicoumarol	HMDB0033758	$C_{19}H_{13}O_6$	$[M+H]^+$	337.0713	337.0712	1.88	No	-	Level 3
Deoxyinosine monophosphate	HMDB0001044	$C_{10}H_{14}N_4O_7P$	$[M+H]^+$	333.0587	333.06	-2.29	No	-	Level 3
Dopamine	HMDB0000073	$C_8H_{10}NO_2$	$[M-H]^-$	152.0724	152.0712	4.58	No	-	Level 3
Dopamine	HMDB0000073	$C_8H_{12}NO_2$	$[M+H]^+$	154.0861	154.0868	-1.01	No	-	Level 3
Dopamine	HMDB0000073	$C_8H_{15}N_2O_2$	$[M+NH_4]^+$	171.1129	171.1134	0.56	No	-	Level 3
Epiguanine	No ID match	$C_6H_8N_5O$	$[M+H]^+$	166.0724	166.0729	0.38	No	-	Level 3
Ethyl 1,2,5,6-tetrahydro-1-methyl-3-pyridinecarboxylate	No ID match	$C_9H_{16}NO_2$	$[M+H]^+$	170.1177	170.1181	0.85	No	-	Level 3
Ethyl 1,2,5,6-tetrahydro-1-methyl-3-pyridinecarboxylate	No ID match	$C_9H_{19}N_2O_2$	$[M+NH_4]^+$	187.1442	187.1447	0.51	No	-	Level 3
FA 10:2;O3	No ID match	$C_{10}H_{17}O_5$	$[M+H]^+$	217.1075	217.1076	2.07	No	-	Level 3
FA 11:0	HMDB0033022	$C_{11}H_{21}O_2$	$[M-H]^-$	185.1548	185.1542	0.52	No	-	Level 3
FA 11:2;O3	No ID match	$C_{11}H_{18}O_3K$	$[M+K]^+$	269.0779	269.0786	-2.53	No	-	Level 3
FA 11:3	No ID match	$C_{11}H_{15}O_2$	$[M-H]^-$	179.1079	179.1072	0.82	No	-	Level 3
FA 11:3;O2	No ID match	$C_{14}H_{29}NO_2K$	$[M+K]^+$	251.067	251.068	-4.05	No	-	Level 3
FA 12:0	HMDB0000637	$C_{12}H_{21}NO_2K$	$[M+K]^+$	239.1396	239.1408	-4.96	No	-	Level 3
FA 12:0	HMDB0000637	$C_{12}H_{23}O_2$	$[M-H]^-$	199.1706	199.1698	1.24	No	-	Level 3
FA 12:1	HMDB0033025	$C_{12}H_{23}O_2$	$[M+H]^+$	199.1694	199.1698	0.72	No	-	Level 3
FA 12:2;O3	No ID match	$C_{11}H_{18}O_5K$	$[M+K]^+$	283.0933	283.0942	-3.29	No	-	Level 3
FA 13:1;O	No ID match	$C_{12}H_{24}O_2K$	$[M+K]^+$	267.135	267.1357	-2.63	No	-	Level 3
FA 13:1;O2	No ID match	$C_{13}H_{24}O_3K$	$[M+K]^+$	283.1298	283.1306	-2.89	No	-	Level 3
FA 14:0	HMDB0000806	$C_{14}H_{27}O_2$	$[M-H]^-$	227.2019	227.2011	1.08	No	-	Level 3
FA 14:1	HMDB0003229	$C_{20}H_{24}NO_6KS$	$[M+K]^+$	265.1557	265.1564	-2.78	No	-	Level 3
FA 15:0	HMDB0000821	$C_{15}H_{29}O_2$	$[M-H]^-$	241.2176	241.2168	1.23	No	-	Level 3
FA 15:4	No ID match	$C_{15}H_{21}O_2$	$[M-H]^-$	233.1549	233.1542	0.84	No	-	Level 3
FA 15:4;O	No ID match	$C_{15}H_{21}O_3$	$[M-H]^-$	249.1498	249.1491	0.73	No	-	Level 3

FA 16:0	HMDB0000220	$C_{16}H_{31}O_2$	$[M-H]^-$	255.2332	255.2324	0.96	No	-		Level 3
FA 16:4	HMDB0033712	$C_{16}H_{23}O_2$	$[M-H]^-$	247.1705	247.1698	0.59	No	-		Level 3
Formylkynurenine	HMDB0000666	$C_{11}H_{13}N_2O_4$	$[M+H]^+$	237.0876	237.0875	2.6	No	-		Level 3
Furfural	HMDB0003344	$C_5H_3O_2$	$[M-H]^-$	95.0139	95.0133	0.49	No	-		Level 3
Furfural	HMDB0003344	$C_5H_5O_2$	$[M+H]^+$	97.0284	97.029	-0.06	No	-		Level 3
Furfuranol	HMDB0033864	$C_5H_5O_2$	$[M-H]^-$	97.0295	97.029	-0.04	No	-		Level 3
Furfuranol	HMDB0033864	$C_5H_7O_2$	$[M+H]^+$	99.0441	99.0446	0.44	No	-		Level 3
Aminobutyryl-lysine	HMDB0033100	$C_{10}H_{22}N_3O_3$	$[M+H]^+$	232.1661	232.1661	2.29	No	-		Level 3
Guanidinobutyrate	HMDB0000677	$C_5H_{12}N_3O_2$	$[M+H]^+$	146.0924	146.093	-0.02	No	-		Level 3
γ-Glu-gln	HMDB0033459	$C_{10}H_{18}N_3O_6$	$[M+H]^+$	276.1199	276.1196	3.21	No	-		Level 3
Glutamate	HMDB0000148	$C_5H_{10}NO_4$	$[M+H]^+$	148.0604	148.061	-0.24	No	-		Level 3
Glutamate	HMDB0000148	$C_5H_9NO_4Na$	$[M+Na]^+$	170.0425	170.0424	0.71	No	-		Level 3
Glutaral	No ID match	$C_5H_9O_2$	$[M+H]^+$	101.0598	101.0603	0.93	No	-		Level 3
Glutathione	HMDB0000125	$C_{10}H_{17}N_3O_4K$	$[M+K]^+$	346.0475	346.047	1.55	No	-		Level 3
Glutathione	HMDB0000125	$C_{10}H_{17}N_3O_6SNa$	$[M+Na]^+$	330.0737	330.073	2.04	No	-		Level 3
Glutathione	HMDB0000125	$C_{10}H_{18}N_3O_6S$	$[M+H]^+$	308.0919	308.0916	2.65	No	-		Level 3
Glycerophosphorylethanolamine	HMDB0000136	$C_5H_{15}NO_6P$	$[M+H]^+$	216.0636	216.0637	2.08	No	-		Level 3
Glycylglycine	HMDB0000714	$C_4H_9N_2O_3$	$[M+H]^+$	133.0608	133.0613	0.23	No	-		Level 3
Guanine	HMDB0000132	$C_5H_5N_5ONa$	$[M+Na]^+$	174.0387	174.0386	0.4	No	-		Level 3
Guanine	HMDB0000132	$C_5H_6N_5O$	$[M+H]^+$	152.0566	152.0572	-0.57	Yes		11.82	Level 2
Hercynine	HMDB0003251	$C_9H_{16}N_3O_2$	$[M+H]^+$	198.1238	198.1243	0.49	No	-		Level 3
Hippurate	HMDB0000712	$C_9H_{10}NO_3$	$[M+H]^+$	180.0656	180.0661	0.44	No	-		Level 3
Hippurate	HMDB0000712	$C_9H_{13}N_2O_3$	$[M+NH_4]^+$	197.0921	197.0926	0.16	No	-		Level 3
His-Tyr	HMDB0029060	$C_{15}H_{18}N_4O_3K$	$[M+K]^+$	357.0973	357.096	3.74	No	-		Level 3

Histidinol	HMDB0003434	$C_6H_{12}N_3O$	$[M+H]^+$	142.0975	142.098	0.08	No	-	Level 3
Histidinol	HMDB0003434	$C_6H_{15}N_4O$	$[M+NH_4]^+$	159.1241	159.1246	0.4	No	-	Level 3
Histidylphenylalanine	HMDB0028893	$C_{14}H_{18}N_4O_3K$	$[M+K]^+$	341.1024	341.101	3.96	No	-	Level 3
Hypoxanthine	HMDB0000157	$C_5H_5N_4O$	$[M+H]^+$	137.0459	137.0463	0.82	Yes	10.12	Level 2
Indole	HMDB0002329	C_8H_7NNa	$[M+Na]^+$	140.0468	140.0471	-1.92	No	-	Level 3
Indole-5,6-quinone	No ID match	$C_8H_5NO_2Na$	$[M+Na]^+$	170.0211	170.0213	-0.88	No	-	Level 3
Isobutanal	HMDB0032987	$C_4H_{12}NO$	$[M+NH_4]^+$	90.0914	90.0919	0.66	No	-	Level 3
Isobutanal	HMDB0032987	C_4H_9O	$[M+H]^+$	73.0648	73.0653	0.12	No	-	Level 3
Kynurenic acid	HMDB0000715	$C_{10}H_8NO_3$	$[M+H]^+$	190.0499	190.0504	0.16	No	-	Level 3
L-Alanine	HMDB0000161	$C_3H_8NO_2$	$[M+H]^+$	90.055	90.0555	0.5	No	-	Level 3
L-Arginine	HMDB0000517	$C_6H_{15}N_4O_2$	$[M+H]^+$	175.1191	175.1195	0.84	No	-	Level 3
L-Citrulline	HMDB0000904	$C_6H_{13}NO_2K$	$[M+K]^+$	214.0582	214.0589	-3.03	No	-	Level 3
L-Cysteate	HMDB0000063	$C_3H_{11}N_2O_5S$	$[M+NH_4]^+$	187.039	187.0389	3.64	No	-	Level 3
L-Cysteinylglycine	HMDB0000078	$C_5H_{11}N_2O_3S$	$[M+H]^+$	179.0485	179.049	0.06	No	-	Level 3
L-Glutamine	HMDB0000641	$C_5H_{10}N_2O_3K$	$[M+K]^+$	185.0324	185.0323	0.54	No	-	Level 3
L-Glutamine	HMDB0000641	$C_5H_{10}N_2O_3Na$	$[M+Na]^+$	169.0584	169.0584	0.22	No	-	Level 3
L-Glutamine	HMDB0000641	$C_5H_{11}N_2O_3$	$[M+H]^+$	147.0764	147.077	-0.13	Yes	15.96	Level 2
L-Glutaminyl-L-proline	HMDB0028994	$C_{10}H_{17}NO_3K$	$[M+K]^+$	282.0856	282.0851	1.9	No	-	Level 3
L-Histidine	HMDB0000177	$C_6H_{10}N_3O_2$	$[M+H]^+$	156.0769	156.0773	0.94	Yes	10.78	Level 2
L-Histidine	HMDB0000684	$C_6H_9N_3O_2Na$	$[M+Na]^+$	178.0581	178.0587	-3.35	No	-	Level 3
L-Kynurenine	HMDB0000182	$C_{10}H_{13}N_2O_3$	$[M+H]^+$	209.0923	209.0926	1.1	No	-	Level 3
L-Lysine	HMDB0000182	$C_6H_{15}N_2O_2$	$[M+H]^+$	147.1128	147.1134	-0.03	No	-	Level 3
L-Norleucine	HMDB0001645	$C_{10}H_{12}N_4O_6K$	$[M+K]^+$	170.0577	170.0578	-0.51	No	-	Level 3
L-Norleucine	HMDB0001645	$C_6H_{14}NO_2$	$[M+H]^+$	132.1019	132.1025	-0.04	Yes	10.65	Level 2

L-Phenylalanine	HMDB0001645	$C_9H_{12}NO_2$	$[M+H]^+$	166.0864	166.0868	0.87	Yes	12.66	Level 2
L-Phenylalanine	HMDB0000159	$C_9H_{15}N_2O_2$	$[M+NH_4]^+$	183.1129	183.1134	0.52	No	-	Level 3
L-Proline	HMDB0000162	$C_5H_{10}NO_2$	$[M+H]^+$	116.0706	116.0712	-0.04	No	-	Level 3
L-Proline	HMDB0000162	$C_5H_9NO_2K$	$[M+K]^+$	154.0259	154.0265	-3.81	No	-	Level 3
L-Serine	HMDB0000187	$C_3H_8NO_3$	$[M+H]^+$	106.0499	106.0504	0.28	No	-	Level 3
L-Tyrosine	HMDB0000158	$C_9H_{11}NOK$	$[M+K]^+$	220.037	220.0371	-0.23	No	-	Level 3
L-Tyrosine	HMDB0000158	$C_9H_{12}NO_3$	$[M+H]^+$	182.0813	182.0817	0.71	No	-	Level 3
L-Tyrosine	HMDB0000158	$C_9H_{15}N_2O_3$	$[M+NH_4]^+$	199.1078	199.1083	0.41	No	-	Level 3
L-Valine	HMDB0000158	$C_5H_{11}NK$	$[M+K]^+$	156.0416	156.0421	-3.44	No	-	Level 3
L-Valine	HMDB0000883	$C_5H_{12}NO_2$	$[M+H]^+$	118.0863	118.0868	0.38	No	-	Level 3
L-Xylonic acid	HMDB0000612	$C_5H_9O_6$	$[M-H]^-$	165.0409	165.0399	2.64	No	-	Level 3
Lactate	HMDB0000190	$C_3H_5O_3$	$[M-H]^-$	89.0245	89.0239	0.91	No	-	Level 3
Leu-Gln	HMDB0028999	$C_{11}H_{22}N_3O_4$	$[M+H]^+$	260.1615	260.161	3.91	No	-	Level 3
Leucamine	No ID match	$C_5H_{14}N$	$[M+H]^+$	88.1121	88.1126	0.28	No	-	Level 3
Leucyltyrosine	HMDB0028895	$C_{15}H_{23}N_2O_4$	$[M+H]^+$	295.1659	295.1658	2.26	No	-	Level 3
Linoleamide	HMDB0002076	$C_{18}H_{34}NO$	$[M+H]^+$	280.2644	280.264	3.25	No	-	Level 3
LPA O-10:2	No ID match	$C_{13}H_{26}O_6P$	$[M+H]^+$	309.1452	309.1467	-3.08	No	-	Level 3
Lys-Pro	HMDB0028899	$C_{11}H_{22}N_3O_3$	$[M+H]^+$	244.1663	244.1661	3	No	-	Level 3
Methionine sulfone	HMDB0002026	$C_4H_{13}N_2O_5S$	$[M+NH_4]^+$	201.0547	201.0545	3.63	No	-	Level 3
Methyl pyrrole	No ID match	$C_5H_{11}N_2$	$[M+NH_4]^+$	99.0917	99.0922	0.26	No	-	Level 3
Methyl pyrrole	No ID match	C_5H_8N	$[M+H]^+$	82.0651	82.0657	-0.31	No	-	Level 3
Methyl vinyl ketone	HMDB0033176	$C_4H_{10}NO$	$[M+NH_4]^+$	88.0757	88.0762	0.11	No	-	Level 3
Methylguanidine	HMDB0001964	$C_2H_8N_3$	$[M+H]^+$	74.0713	74.0718	0.36	No	-	Level 3
N-(2,3,4-Trimethoxybenzoyl)glycine	No ID match	$C_{12}H_{15}NO_4K$	$[M+K]^+$	308.0535	308.0531	1.31	No	-	Level 3
N-(2,3,4-Trimethoxybenzoyl)glycine	No ID match	$C_{12}H_{16}NO_6$	$[M+H]^+$	270.0982	270.0978	3.65	No	-	Level 3

N-(3-acetamidopropyl)pyrrolidin-2-one	No ID match	$C_9H_{20}N_3O_2$	$[M+NH_4]^+$	202.155	202.1556	-0.02	No	-	Level 3
N-[(2S)-2-Hydroxypropanoyl]methionine	No ID match	$C_8H_{19}N_2O_4S$	$[M+NH_4]^+$	239.1065	239.1066	2.07	No	-	Level 3
N-Acetyl-5-oxo-L-norvaline	No ID match	$C_7H_{12}NO_4$	$[M+H]^+$	174.0762	174.0766	0.66	No	-	Level 3
N-Acetyl-L-phenylalanine	HMDB0000702	$C_{11}H_{13}NO_2K$	$[M+K]^+$	246.053	246.0527	1.21	No	-	Level 3
N-Acetyl-L-phenylalanine	HMDB0000702	$C_{11}H_{14}NO_3$	$[M+H]^+$	208.097	208.0974	0.86	No	-	Level 3
N-Acetyl-L-phenylalanine	HMDB0000702	$C_{11}H_{17}N_2O_3$	$[M+NH_4]^+$	225.1238	225.1239	1.91	No	-	Level 3
N-Acetylputrescine	HMDB0001365	$C_6H_{15}N_2O$	$[M+H]^+$	131.118	131.1184	0.85	Yes	16.8	Level 2
N-Acetylvanilalanine	No ID match	$C_{12}H_{16}NO_5$	$[M+H]^+$	254.1031	254.1028	3.15	No	-	Level 3
N-Benzoylaspartic acid	No ID match	$C_{11}H_{12}NO_5$	$[M+H]^+$	238.0715	238.0715	2.1	No	-	Level 3
Heptanoic acid	HMDB0000849	$C_7H_{13}O_2$	$[M-H]^-$	129.0922	129.0916	0.74	No	-	Level 3
Heptanoic acid	HMDB0000849	$C_7H_{15}O_2$	$[M+H]^+$	131.1067	131.1072	0.33	No	-	Level 3
N6-Acetyl-L-lysine	HMDB0000439	$C_8H_{17}N_2O_3$	$[M+H]^+$	189.1235	189.1239	0.69	Yes	11.21	Level 2
NAE 10:1	No ID match	$C_{12}H_{24}NO_2$	$[M+H]^+$	214.1804	214.1807	1.14	No	-	Level 3
NAE 10:2	No ID match	$C_{12}H_{20}O_5K$	$[M+K]^+$	250.1195	250.1204	-3.54	No	-	Level 3
NAE 10:2	No ID match	$C_{12}H_{22}NO_2$	$[M+H]^+$	212.1647	212.1651	0.92	No	-	Level 3
NAE 12:0	No ID match	$C_{15}H_{28}O_3K$	$[M+K]^+$	282.1821	282.183	-3.14	No	-	Level 3
NAE 16:1	No ID match	$C_{18}H_{36}NO_2$	$[M+H]^+$	298.2747	298.2746	2.16	No	-	Level 3
NAE 16:2	No ID match	$C_{18}H_{34}NO_2$	$[M+H]^+$	296.2592	296.259	2.68	No	-	Level 3
NAT 11:1;O4	No ID match	$C_{13}H_{25}NO_8SNa$	$[M+Na]^+$	378.1187	378.1193	-1.61	No	-	Level 3
NAT 12:0;O4	No ID match	$C_{14}H_{29}NO_8SNa$	$[M+Na]^+$	394.1497	394.1506	-2.31	No	-	Level 3
NAT 12:1;O4	No ID match	$C_{14}H_{27}NO_8SNa$	$[M+Na]^+$	392.1343	392.135	-1.68	No	-	Level 3
NAT 12:2;O3	No ID match	$C_{14}H_{25}NO_7SNa$	$[M+Na]^+$	374.1237	374.1244	-1.86	No	-	Level 3
NAT 12:2;O4	No ID match	$C_{14}H_{25}NO_8SNa$	$[M+Na]^+$	390.1186	390.1193	-1.82	No	-	Level 3
NAT 12:3;O4	No ID match	$C_{14}H_{23}NO_8SNa$	$[M+Na]^+$	388.103	388.1037	-1.7	No	-	Level 3
Nicotinamide	HMDB0001406	$C_6H_7N_2O$	$[M+H]^+$	123.0553	123.0558	0.09	Yes	7.33	Level 2
Nicotinyl methylamide	No ID match	$C_7H_9N_2O$	$[M+H]^+$	137.071	137.0715	0.44	No	-	Level 3

Normetanephrine	HMDB0000425	$C_9H_{14}NO_3$	$[M+H]^+$	184.0969	184.0974	0.43	No	-	Level 3
Normetanephrine	HMDB0000425	$C_9H_{17}N_2O_3$	$[M+NH_4]^+$	201.1234	201.1239	0.15	No	-	Level 3
Octadecanoic acid	HMDB0000827	$C_{18}H_{35}O_2$	$[M-H]^-$	283.2645	283.2637	0.87	No	-	Level 3
Oxo-3-pyridinebutanal	No ID match	$C_9H_{10}NO_2$	$[M+H]^+$	164.0707	164.0712	0.58	No	-	Level 3
Oxo-3-pyridinebutanal	No ID match	$C_9H_{13}N_2O_2$	$[M+NH_4]^+$	181.0972	181.0977	0.25	No	-	Level 3
Pantothenate	HMDB0000210	$C_9H_{18}NO_5$	$[M+H]^+$	220.1184	220.1185	2.04	No	-	Level 3
Phenethylamine	HMDB0000872	$C_8H_{12}N$	$[M+H]^+$	122.0965	122.097	0.61	No	-	Level 3
Phenylacetylglycine	HMDB0000717	$C_{10}H_{11}NO_2K$	$[M+K]^+$	232.0372	232.0371	0.64	No	-	Level 3
Phenylacetylglycine	HMDB0000717	$C_{10}H_{12}NO_3$	$[M+H]^+$	194.0813	194.0817	0.67	No	-	Level 3
Phenylacetylglycine	HMDB0000717	$C_{10}H_{15}N_2O_3$	$[M+NH_4]^+$	211.108	211.1083	1.33	No	-	Level 3
Phenylbutyric Acid	HMDB0001887	$C_{10}H_{12}O_2Na$	$[M+Na]^+$	187.0732	187.073	1.34	No	-	Level 3
Phenylethyl alcohol	HMDB0001895	$C_8H_{14}NO$	$[M+NH_4]^+$	140.1071	140.1075	0.78	No	-	Level 3
Phenylglyoxal	No ID match	$C_8H_{10}NO_2$	$[M+NH_4]^+$	152.0705	152.0712	-0.69	No	-	Level 3
Phenylglyoxal	No ID match	$C_8H_7O_2$	$[M+H]^+$	135.0441	135.0446	0.33	No	-	Level 3
Phenylpyruvate	HMDB0000669	$C_9H_7O_3$	$[M-H]^-$	163.0402	163.0395	0.8	No	-	Level 3
Phenylpyruvate	HMDB0000669	$C_9H_9O_3$	$[M+H]^+$	165.0547	165.0552	0.48	No	-	Level 3
Pterin	HMDB0000929	$C_6H_6N_5O$	$[M+H]^+$	164.0568	164.0572	0.69	No	-	Level 3
Putrescine	HMDB0001414	$C_4H_{13}N_2$	$[M+H]^+$	89.1073	89.1079	-0.27	No	-	Level 3
Pyrazine	No ID match	$C_4H_5N_2$	$[M+H]^+$	81.0448	81.0453	0.94	No	-	Level 3
Pyrazine	No ID match	$C_4H_8N_3$	$[M+NH_4]^+$	98.0713	98.0718	0.27	No	-	Level 3
Pyrazinoate	No ID match	$C_5H_8N_3O_2$	$[M+NH_4]^+$	142.0611	142.0617	-0.02	No	-	Level 3
Pyridine	No ID match	C_5H_6N	$[M+H]^+$	80.0495	80.05	0.31	No	-	Level 3
Pyridine	No ID match	$C_5H_9N_2$	$[M+NH_4]^+$	97.0761	97.0766	0.78	No	-	Level 3
Pyridoxal	HMDB0000179	$C_8H_{10}NO_3$	$[M+H]^+$	168.0656	168.0661	0.48	No	-	Level 3
Pyridoxal	HMDB0000179	$C_8H_{13}N_2O_3$	$[M+NH_4]^+$	185.0922	185.0926	0.71	No	-	Level 3

Pyridoxine	HMDB0000262	$C_8H_{15}N_2O_3$	$[M+NH_4]^+$	187.1078	187.1083	0.43	No	-	Level 3
Pyrrolidine	No ID match	$C_4H_{10}N$	$[M+H]^+$	72.0808	72.0813	0.35	No	-	Level 3
Pyrrolidine	No ID match	C_4H_9NK	$[M+K]^+$	110.0363	110.0367	-3.24	No	-	Level 3
Allyl propanethioate	No ID match	$C_6H_{14}NOS$	$[M+NH_4]^+$	148.0797	148.0796	4.31	No	-	Level 3
Salicyluric acid	HMDB0000877	$C_9H_{10}NO_4$	$[M+H]^+$	196.0606	196.061	0.84	No	-	Level 3
SPB 16:1;O	No ID match	$C_{16}H_{34}NO$	$[M+H]^+$	256.2643	256.264	3.16	No	-	Level 3
Spermidine	HMDB0001257	$C_7H_{20}N_3$	$[M+H]^+$	146.1651	146.1657	-0.5	No	-	Level 3
Spermine	HMDB0001259	$C_{10}H_{27}N_4$	$[M+H]^+$	203.2232	203.2236	0.88	No	-	Level 3
ST 19:4;O8	No ID match	$C_{13}H_{24}O_4K$	$[M+K]^+$	419.1088	419.1103	-3.52	No	-	Level 3
ST 20:5;O3;S	No ID match	$C_{19}H_{24}O_8K$	$[M+K]^+$	445.0961	445.0956	1.14	No	-	Level 3
Stachydrine	HMDB0003219	$C_7H_{14}NO_2$	$[M+H]^+$	144.1019	144.1025	-0.04	No	-	Level 3
Homospermidine	No ID match	$C_8H_{22}N_3$	$[M+H]^+$	160.1809	160.1814	0.48	No	-	Level 3
Tryptophol	HMDB0001707	$C_9H_{11}NO_3K$	$[M+K]^+$	200.0469	200.0472	-1.61	No	-	Level 3
Tyramine	HMDB0000305	$C_7H_{11}NO_2K$	$[M+K]^+$	176.0469	176.0472	-1.82	No	-	Level 3
Tyramine	HMDB0000305	$C_8H_{12}NO$	$[M+H]^+$	138.0914	138.0919	0.43	No	-	Level 3
Tyramine	HMDB0000305	$C_8H_{15}N_2O$	$[M+NH_4]^+$	155.1178	155.1184	-0.57	No	-	Level 3
Xanthosine	HMDB0000783	$C_6H_{12}KS$	$[M+K]^+$	323.0404	323.0388	4.82	No	-	Level 3

Table S1. List of annotated metabolite features detected in individual cell from AP-MALDI-MSI dataset acquired using DHB matrix. The table includes all annotated metabolite features with corresponding adducts (n = 292 annotated adducts corresponding to n = 218 unique metabolites; multiple entries per metabolite reflect different detected adduct forms). Metabolite annotations were supported by LC–MS/MS validation using retention time (RT) and fragmentation matching to reference standards.

Metabolite	HMDB Identifier	Formula	Adduct	Observed m/z	Theoretical m/z	Mass Error (ppm)	LC-MS Confirmed	LC-MS RT (min)	Identification Level
Pyridine	HMDB0000926	$C_5H_9N_2$	[M+NH4]+	97.0756	97.0766	-4.36	No	-	Level 3
Cyanohydroxy butene	No ID match	C_5H_8NO	[M+H]+	98.0596	98.0606	-4.49	No	-	Level 3
Furfuranol	HMDB0013742	$C_5H_7O_2$	[M+H]+	99.0436	99.0446	-4.61	No	-	Level 3
1H-Pyrazol-4-ylmethanol	HMDB0060768	$C_4H_7N_2O$	[M+H]+	99.0548	99.0558	-4.94	No	-	Level 3
2-Methylfuran	HMDB0013749	C_5H_0NO	[M+NH4]+	100.0752	100.0762	-4.9	No	-	Level 3
Cyclohexylamine	HMDB0031404	C_6H_4N	[M+H]+	100.1116	100.1126	-4.75	No	-	Level 3
Cyclopentanone	HMDB0031407	C_5H_2NO	[M+NH4]+	102.091	102.0919	-3.33	No	-	Level 3
Triethylamine	HMDB0032539	C_6H_6N	[M+H]+	102.1273	102.1283	-4.16	No	-	Level 3
Benzaldehyde	HMDB0006115	C_7H_7O	[M+H]+	107.0489	107.0497	-2.25	No	-	Level 3
Benzoquinone	HMDB0003364	$C_6H_5O_2$	[M+H]+	109.0283	109.029	-0.97	No	-	Level 3
Pyrrolidine	HMDB0031641	C_4H_9KN	[M+K]+	110.0362	133.0259	-4.15	No	-	Level 3
2-Amino-4-methylpyrimidine	No ID match	$C_5H_8N_3$	[M+H]+	110.0712	110.0718	-0.66	Yes	13.39	Level 2
Cytosine	HMDB0000630	$C_4H_6N_3O$	[M+H]+	112.0504	112.0511	-1.23	Yes	10.25	Level 2
Histamine	HMDB0000870	$C_5H_0N_3$	[M+H]+	112.0868	112.0875	-1.1	Yes	8.60	Level 2
2-pyridone	HMDB0013751	$C_5H_9N_2O$	[M+NH4]+	113.0708	113.0715	-1.23	No	-	Level 3
1-pyrroline-5-carboxylic acid	HMDB0001301	$C_5H_8NO_2$	[M+H]+	114.0549	114.0555	-0.48	No	-	Level 3
3-Amino-2-piperidinone	No ID match	C_5HN_2O	[M+H]+	115.0865	115.0871	-0.77	No	-	Level 3
Cyclohexanone	No ID match	C_6H_4NO	[M+NH4]+	116.1069	116.1075	-0.78	No	-	Level 3
Benzoate	HMDB0304270	$C_7H_7O_2$	[M+H]+	123.0439	123.0446	-1.27	No	-	Level 3
Nicotinamide	HMDB0001406	$C_6H_7N_2O$	[M+H]+	123.0552	123.0558	-0.72	Yes	7.33	Level 2
2-Methoxy-3-methylpyrazine	HMDB0031852	$C_6H_9N_2O$	[M+H]+	125.0709	125.0715	-0.31	No	-	Level 3
2-AEP	HMDB0011747	$C_2H_9NO_3P$	[M+H]+	126.0311	126.032	-2.83	No	-	Level 3
(3E)-2,5-Dioxo-3-hexenal	No ID match	$C_6H_7O_3$	[M+H]+	127.0389	127.0395	-0.56	No	-	Level 3
4-Aminophenol	No ID match	C_6HN_2O	[M+NH4]+	127.0865	127.0871	-0.7	No	-	Level 3
2-Amino-4-methylpyrimidine	No ID match	C_5HN_4	[M+NH4]+	127.0977	127.0984	-0.96	No	-	Level 3

8-Azabicyclo[3.2.1]octan-3-ol	No ID match	C_7H_4NO	[M+H]+	128.1069	128.1075	-0.7	No	-	Level 3
2-Acetyl-1-pyrroline	No ID match	$C_6H_3N_2O$	[M+NH4]+	129.1022	129.1028	-0.3	No	-	Level 3
5-Oxoproline	HMDB0000267	$C_5H_8NO_3$	[M+H]+	130.0499	130.0504	0.23	No	-	Level 3
n-heptanoic acid	HMDB0000666	$C_7H_5O_2$	[M+H]+	131.1067	131.1072	0.33	No	-	Level 3
N-Acetylputrescine	HMDB0002064	$C_6H_5N_2O$	[M+H]+	131.1179	131.1184	0.08	Yes	16.8	Level 2
Agmatine	HMDB0001432	$C_5H_5N_4$	[M+H]+	131.1291	131.1297	-0.17	No	-	Level 3
3-Amino-2-piperidinone	No ID match	$C_5H_4N_3O$	[M+NH4]+	132.1131	132.1137	-0.29	No	-	Level 3
Cytosine	HMDB0000630	$C_4H_5N_3NaO$	[M+Na]+	134.0325	134.0325	0.13	No	-	Level 3
Phenylglyoxal	No ID match	$C_8H_7O_2$	[M+H]+	135.044	135.0446	-0.42	No	-	Level 3
Cyanohydroxy butene	No ID match	C_5H_7KNO	[M+K]+	136.0156	136.0159	-2.36	No	-	Level 3
Adenine	HMDB0000034	$C_5H_6N_5$	[M+H]+	136.0618	136.0623	0.21	Yes	11.99	Level 2
2-Aminoacetophenone	HMDB0032628	C_8H_0NO	[M+H]+	136.0757	136.0762	0.07	No	-	Level 3
4-Aminobenzoate	HMDB0304171	$C_7H_8NO_2$	[M+H]+	138.0549	136.0762	0.07	No	-	Level 3
Succinamide	HMDB0258539	$C_4H_8N_2NaO_2$	[M+Na]+	139.0478	138.0555	-0.4	No	-	Level 3
Nicotinamide	HMDB0001406	$C_6H_0N_3O$	[M+NH4]+	140.0818	140.0824	-0.27	No	-	Level 3
Histidinol	HMDB0003431	$C_6H_2N_3O$	[M+H]+	142.0975	142.098	0.08	No	-	Level 3
Isopelletierine	No ID match	C_8H_6NO	[M+H]+	142.1227	142.1232	0.42	No	-	Level 3
2-Ethyl-4,5-dimethylthiazole	No ID match	$C_7H_5N_2O$	[M+NH4]+	143.1179	143.1184	0.08	No	-	Level 3
g-Guanidinobutyrate	HMDB0003464	$C_5H_2N_3O_2$	[M+H]+	146.0924	146.093	-0.02	No	-	Level 3
Coumarin	HMDB0001218	$C_9H_7O_2$	[M+H]+	147.044	147.0446	-0.38	No	-	Level 3
L-Glutamine	HMDB0000641	$C_5HN_2O_3$	[M+H]+	147.0764	147.077	-0.13	Yes	15.96	Level 2
2-Chromanone	No ID match	$C_9H_9O_2$	[M+H]+	149.0597	149.0603	-0.04	No	-	Level 3
Guanine	HMDB0000132	$C_5H_6N_5O$	[M+H]+	152.0566	152.0572	-0.57	No	-	Level 3
Phenylglyoxal	No ID match	$C_8H_0NO_2$	[M+NH4]+	152.0706	152.0712	-0.03	No	-	Level 3
aminoproline	No ID match	$C_5H_0N_2NaO_2$	[M+Na]+	153.0634	153.0635	-0.31	No	-	Level 3
L-Proline	HMDB0000162	$C_5H_9KNO_2$	[M+K]+	154.026	154.0264	-3.16	No	-	Level 3
2-methyl-1-methylthio-2-butene	No ID match	C_6H_2KS	[M+K]+	155.0294	155.0291	1.75	No	-	Level 3
Tyramine	HMDB0000306	$C_8H_5N_2O$	[M+NH4]+	155.1178	155.1184	-0.57	No	-	Level 3

Betaine	HMDB0000043	C_5HKNO_2	[M+K]+	156.0417	156.0421	-2.79	No	-	Level 3
2-Amino-3-(1H-imidazol-4-yl)propanal	No ID match	$C_6H_3N_4O$	[M+NH4]+	157.1083	157.1083	-0.55	No	-	Level 3
Norpseudopelletierine	No ID match	$C_8H_7N_2O$	[M+NH4]+	157.1335	157.1335	-0.25	No	-	Level 3
D-Erythrose	No ID match	$C_4H_8KO_4$	[M+K]+	159.0052	159.0054	-1.37	No	-	Level 3
Histidinol	HMDB0003431	$C_6H_5N_4O$	[M+NH4]+	159.1239	159.1246	-0.86	No	-	Level 3
2-Amino-3-(1H-imidazol-4-yl)propanal	No ID match	$C_6H_9N_3NaO$	[M+Na]	162.0637	162.0638	-0.51	No	-	Level 3
Epiguanine	No ID match	$C_6H_8N_5O$	[M+H]+	166.0723	166.0729	-0.22	No	-	Level 3
L-Phenylalanine	HMDB0000159	$C_9H_2NO_2$	[M+H]+	166.0862	166.0868	-0.33	Yes	12.66	Level 2
3-Methoxytyramine	HMDB0000022	$C_9H_4NO_2$	[M+H]+	168.1018	168.1025	-0.63	No	-	Level 3
L-Hydroxyproline	HMDB0000725	$C_5H_9KNO_3$	[M+K]	170.0209	170.0213	-2.05	No	-	Level 3
Leucine	HMDB0000687	$C_6H_3KNO_2$	[M+K]	170.0576	170.0582	-0.42	Yes	10.36	Level 2
Ethyl 1,2,5,6-tetrahydro-1-methyl-3-pyridinecarboxylate	No ID match	$C_9H_6NO_2$	[M+H]+	170.1174	170.1176	-0.91	No	-	Level 3
Gallate	HMDB0005807	$C_7H_7O_5$	[M+H]+	171.0286	171.0294	-1.18	No	-	Level 3
3-Sulfino-L-alanine	HMDB0000996	$C_3HN_2O_4S$	[M+NH4]+	171.0439	171.044	2.89	No	-	Level 3
(2R,3R)-1,2,3-Heptanetriol	No ID match	$C_7H_6NaO_3$	[M+Na]	171.0991	171.0992	-0.38	No	-	Level 3
Acetylhistamine	HMDB0013253	$C_7H_5N_4O$	[M+NH4]+	171.1239	171.1246	-0.8	No	-	Level 3
Menadione	HMDB0001892	CH_9O_2	[M+H]+	173.0596	173.0603	-0.61	No	-	Level 3
Guanine	HMDB0000132	$C_5H_5N_5NaO$	[M+Na]	174.0386	174.0386	-0.17	Yes	11.81	Level 2
Threonic acid	HMDB0000943	$C_4H_8KO_5$	[M+K]+	175.0001	175.1195	-0.87	No	-	Level 3
6,7-Dihydroxycoumarin	HMDB0030819	$C_9H_7O_4$	[M+H]+	179.0337	179.0344	-1.04	No	-	Level 3
g-Butyrobetaine	HMDB0001161	$C_7H_5KNO_2$	[M+K]+	184.0732	184.0732	-0.46	No	-	Level 3
1,3,7-Octanetriol	No ID match	$C_8H_8NaO_3$	[M+Na]	185.1147	185.1148	-0.62	No	-	Level 3
3-Methoxytyramine	HMDB0000022	$C_9H_7N_2O_2$	[M+NH4]+	185.1283	185.129	-0.83	No	-	Level 3
1,5-Dimethylbicyclo(3.2.1)octan-8-one oxime	No ID match	$C_0H_2N_2O$	[M+NH4]+	185.1647	185.1654	-0.75	No	-	Level 3
3,4-Dihydroxyphenylacetate	No ID match	$C_8H_2NO_4$	[M+NH4]+	186.076	186.0766	-0.46	No	-	Level 3
(2R,3R)-1,2,3-Heptanetriol	No ID match	$C_7H_6KO_3$	[M+K]+	187.073	187.073	0.27	No	-	Level 3

N(pi)-Methyl-L-histidine	HMDB0000479	$C_7H_5N_4O_2$	[M+NH4]+	187.1188	187.1195	-0.81	No	-	Level 3
Scopoletin	HMDB0034344	$C_0H_9O_4$	[M+H]+	193.0493	193.0501	-1.22	No	-	Level 3
Salicyluric acid	HMDB0000840	$C_9H_0NO_4$	[M+H]+	196.0602	196.061	-1.2	No	-	Level 3
hercynine	HMDB0029422	$C_9H_6N_3O_2$	[M+H]+	198.1235	198.1243	-1.03	No	-	Level 3
Methcathinone	No ID match	C_0H_3KNO	[M+K]+	202.0623	225.0521	-2.83	No	-	Level 3
N-(3-acetamidopropyl)pyrrolidin-2-one	No ID match	$C_9H_{20}N_3O_2$	[M+NH4]+	202.1547	202.1556	-1.5	No	-	Level 3
5-Methoxy-3-indoleaceate	No ID match	CH_2NO_3	[M+H]+	206.0803	206.0817	-4.22	No	-	Level 3
6,8-Dihydroxy-7-methoxy-2H-chromen-2-one	No ID match	$C_0H_9O_5$	[M+H]+	209.0436	209.045	-4.07	No	-	Level 3
5-Nitro-2-propoxyaniline	No ID match	$C_9H_6N_3O_3$	[M+NH4]+	214.1177	214.1192	-4.29	No	-	Level 3
2-Isopropyl-3,5-dimethoxy-6-methylpyrazine	No ID match	$C_0H_{20}N_3O_2$	[M+NH4]+	214.154	214.1556	-4.68	No	-	Level 3
11-Dodecynoic acid	No ID match	$C_2H_{24}NO_2$	[M+NH4]+	214.1792	214.1807	-4.46	No	-	Level 3
1,9-Nonanedithiol	No ID match	$C_9H_{20}KS_2$	[M+K]+	231.0627	254.053	-4.76	No	-	Level 3
3-(4-Hydroxy-3-methoxyphenyl)-2-oxiranecarboxylic acid	No ID match	$C_0H_0NaO_5$	[M+Na]+	233.042	233.042	-0.19	No	-	Level 3
Deoxyribose 1-phosphate	HMDB0001351	C_5HNaO_7P	[M+Na]+	237.0133	237.0135	-0.68	No	-	Level 3
O-propenoyl-D-carnitine	HMDB0013124	$C_0H_7NNaO_4$	[M+Na]+	238.1048	238.105	-0.75	No	-	Level 3
FA 10:2;O2	No ID match	$C_0H_6KO_4$	[M+K]+	239.0677	262.0572	-1.33	No	-	Level 3
propionylcarnitine	HMDB0000824	$C_0H_9NNaO_4$	[M+Na]+	240.1204	240.1206	-0.95	Yes	9.44	Level 2
1D-myo-Inositol 1,2-cyclic phosphate	HMDB0001125	$C_6H_2O_8P$	[M+H]+	243.0254	243.027	-4.25	No	-	Level 3
2-Hydroxy-7-methyloctanedioic acid	No ID match	$C_9H_6KO_5$	[M+K]+	243.0619	251.0668	-4.25	No	-	Level 3
L-fucopyranose 1-phosphate	HMDB0001265	$C_6H_4O_8P$	[M+H]+	245.041	245.0426	-4.42	No	-	Level 3
S-3-oxodecanoyl cysteamine	No ID match	$C_2H_{24}NO_2S$	[M+H]+	246.1526	246.1528	1.52	Yes	3.65	Level 2
FA 11:3;O2	No ID match	CH_6KO_4	[M+K]+	251.0668	251.068	-4.85	No	-	Level 3
Epinephrine 4-sulfate	HMDB0001876	$C_9H_4NO_6S$	[M+H]+	264.0538	264.0542	0.62	No	-	Level 3
2,6-di-tert-butyl-4-ethylphenol	No ID match	$C_6H_{26}KO$	[M+K]+	273.1623	273.1623	2.85	No	-	Level 3
2-(4-Methylthiazol-5-yl)ethyl hexanoate	No ID match	$C_2H_9KNO_2S$	[M+K]+	280.0773	280.0773	1.76	No	-	Level 3

Epinephrine 4-sulfate	HMDB0001876	$C_9H_3NNaO_6S$	[M+Na]+	286.0359	286.0356	1.12	No	-	Level 3
3-Oxo-N-[(3S)-2-oxotetrahydro-3-thiophenyl]octanamide	No ID match	$C_2H_9KNO_3S$	[M+K]+	296.0725	296.0717	2.63	No	-	Level 3
ST 18:1;O	No ID match	$C_8H_{28}KO$	[M+K]+	299.1785	299.1772	4.44	No	-	Level 3
ST 18:0;O	No ID match	$C_8H_{30}KO$	[M+K]+	301.1942	301.1928	4.57	No	-	Level 3
(2E)-1-(4-Hydroxy-1-benzofuran-5-yl)-3-phenyl-2-propen-1-one	No ID match	$C_7H_2KO_3$	[M+K]+	303.0433	303.0418	4.94	No	-	Level 3
ST 19:1;O	No ID match	$C_9H_{30}KO$	[M+K]+	313.194	326.0831	3.76	No	-	Level 3
NAT 11:3	No ID match	$C_3H_2KNO_4S$	[M+K]+	326.0831	326.0831	2.49	No	-	Level 3
D-Sedoheptulose 7-phosphate	HMDB0001068	$C_7H_5KO_0P$	[M+K]+	329.0018	329.2257	-4.99	No	-	Level 3
ST 20:0;O	No ID match	$C_{20}H_{34}KO$	[M+K]+	329.2257	329.2241	4.79	No	-	Level 3
Farnesylcysteine	HMDB0011627	$C_8H_{35}N_2O_2S$	[M+NH4]+	343.2418	343.2414	1.24	No	-	Level 3
ST 20:5;O3;S	No ID match	$C_{20}H_{24}KNO_6S$	[M+K]+	445.0939	445.0956	-3.8	No	-	Level 3
PI-Cer 41:3;O2	No ID match	$C_{47}H_{88}NNaOP$	[M+Na]+	896.6007	896.5987	2.21	No	-	Level 3

Table S2. List of annotated metabolite features detected in individual cells from AP-MALDI-MSI datasets acquired under monoculture conditions (GIN8, GIN28, GIN31 glioblastoma cells and human astrocytes). The table includes all annotated metabolite features with corresponding adducts (*n* = 116 annotated adducts corresponding to *n* = 104 unique metabolites; multiple entries per metabolite reflect different detected adduct forms). Where available, annotations were further supported by LC–MS/MS data through retention time (RT) and/or fragmentation matching.

Metabolite	*HMDB Identifier*	*Formula*	*Adduct*	*Observed m/z*	*Theoretical m/z*	*Mass Error (ppm)*	*LC-MS Confirmed*	*LC-MS RT (min)*	*Identification Level*
1-(4-Aminobutyl)urea	No ID match	$C_5H_{14}N_3O$	$[M+H]^+$	132.1131	132.1137	-0.17	No	-	Level 3
Glycerophosphoinositol	HMDB0011649	$C_9H_{19}O_{11}PNa$	$[M+Na]^+$	357.0567	357.0557	-4.95	No	-	Level 3
Succinamide	No ID match	$C_4H_8N_2O_2Na$	$[M+Na]^+$	139.0478	139.0478	0.21	No	-	Level 3
1-pyrroline-5-carboxylic acid	HMDB0000086	$C_5H_8NO_2$	$[M+H]^+$	114.0549	114.0555	-0.35	No	-	Level 3
1,2,3-Heptanetriol	No ID match	$C_7H_{16}O_3Na$	$[M+Na]^+$	171.099	171.0992	2.89	No	-	Level 3
1,2,3-Heptanetriol	No ID match	$C_7H_{17}O_3$	$[M+H]^+$	149.1172	149.1178	-0.04	No	-	Level 3
1,3,7-Octanetriol	No ID match	$C_8H_{18}O_3Na$	$[M+Na]^+$	185.1147	185.1148	2.40	No	-	Level 3
1,9-Nonanedithiol	No ID match	$C_9H_{20}S_2K$	$[M+K]^+$	231.0627	231.0638	-0.57	No	-	Level 3
1D-myo-Inositol 1,2-cyclic phosphate	No ID match	$C_6H_{12}O_8P$	$[M+H]^+$	243.0254	243.0270	-1.37	No	-	Level 3
2-Acetamidohexanedioic acid	No ID match	$C_8H_{14}NO_5$	$[M+H]^+$	204.0859	204.0872	-4.05	No	-	Level 3
2-Acetyl-1-pyrroline	No ID match	$C_6H_{13}N_2O$	$[M+NH_4]^+$	129.1022	129.1028	-0.42	No	-	Level 3
2-Aminoethylphosphonic acid	No ID match	$C_2H_9NO_3P$	$[M+H]^+$	126.0311	126.0320	-0.31	No	-	Level 3
2-Amino-4-methylpyrimidine	No ID match	$C_5H_8N_3$	$[M+H]^+$	110.0712	110.0718	-4.14	No	-	Level 3
2-Amino-4-methylpyrimidine	No ID match	$C_5H_{11}N_4$	$[M+NH_4]^+$	127.0978	127.0984	0.09	No	-	Level 3
2-Aminoacetophenone	No ID match	$C_8H_{10}NO$	$[M+H]^+$	136.0757	136.0762	0.21	No	-	Level 3
2-Aminohistamine	No ID match	$C_5H_{10}N_3$	$[M+H]^+$	112.0868	112.0875	-1.23	No	-	Level 3
2-Aminohistamine	No ID match	$C_5H_{13}N_4$	$[M+NH_4]^+$	129.1134	129.1140	-0.30	No	-	Level 3
2-Chromanone	No ID match	$C_9H_9O_2$	$[M+H]^+$	149.0597	149.0603	-0.13	No	-	Level 3
2-Ethyl-4,5-dimethylthiazole	No ID match	$C_7H_{15}N_2O$	$[M+NH_4]^+$	143.118	143.1184	0.42	No	-	Level 3
2-Isopropyl-3,5-dimethoxy-6-methylpyrazine	No ID match	$C_{10}H_{20}N_3O_2$	$[M+NH_4]^+$	214.154	214.1556	-4.29	No	-	Level 3
2-Methoxy-3-methylpyrazine	No ID match	$C_6H_9N_2O$	$[M+H]^+$	125.0709	125.0715	-0.72	No	-	Level 3
2-methyl-1-methylthio-2-butene	No ID match	$C_6H_{12}SK$	$[M+K]^+$	155.0294	155.0291	-0.46	No	-	Level 3
2-Methylfuran	HMDB0013749	$C_5H_{10}NO$	$[M+NH_4]^+$	100.0752	100.0762	-4.78	No	-	Level 3
2-Oxo-5-pentyltetrahydro-3-furancarboxylic acid	No ID match	$C_{10}H_{16}O_4K$	$[M+K]^+$	239.0676	239.0680	-1.74	No	-	Level 3

2-pyridone	HMDB0013751	$C_5H_9N_2O$	$[M+NH_4]^+$	113.0709	113.0715	-0.94	No	-	Level 3
3-(4-Hydroxy-3-methoxyphenyl)-2-oxiranecarboxylic acid	No ID match	$C_{10}H_{10}O_5Na$	$[M+Na]^+$	233.0419	233.0420	-4.76	No	-	Level 3
3-(4-Hydroxyphenyl)pyruvate	HMDB0000235	$C_9H_9O_4$	$[M+H]^+$	181.0494	181.0501	-1.14	No	-	Level 3
3-Amino-2-piperidinone	No ID match	$C_5H_{11}N_2O$	$[M+H]^+$	115.0865	115.0871	-0.77	No	-	Level 3
3-Methoxy-4-hydroxymandelate	HMDB0000291	$C_9H_{10}O_5Na$	$[M+Na]^+$	221.0428	221.0422	3.57	No	-	Level 3
3-Methoxytyramine	HMDB0000022	$C_9H_{14}NO_2$	$[M+H]^+$	168.1018	168.1025	0.08	No	-	Level 3
3-Methoxytyramine	HMDB0000022	$C_9H_{17}N_2O_2$	$[M+NH_4]^+$	185.1283	185.1290	-0.83	No	-	Level 3
3-Sulfino-L-alanine	No ID match	$C_3H_{11}N_2O_4S$	$[M+NH_4]^+$	171.0439	171.0440	-1.18	No	-	Level 3
3,4-Dihydroxyphenylacetate	No ID match	$C_8H_{12}NO_4$	$[M+NH_4]^+$	186.076	186.0766	-0.75	No	-	Level 3
4-Aminobenzoate	HMDB0304171	$C_7H_8NO_2$	$[M+H]^+$	138.0549	138.0555	0.07	No	-	Level 3
4-Aminophenol	No ID match	$C_6H_{11}N_2O$	$[M+NH_4]^+$	127.0866	127.0871	-0.56	No	-	Level 3
4-Hydroxybenzoic acid	HMDB0000500	$C_7H_7O_3$	$[M+H]^+$	139.039	139.0395	-0.40	No	-	Level 3
5-(2-Carboxyethyl)-2-hydroxyphenyl beta-D-glucopyranosiduronic acid	No ID match	$C_{15}H_{18}O_{10}K$	$[M+K]^+$	397.0525	397.0532	-2.89	No	-	Level 3
Indole-5,6-quinone	HMDB0006779	$C_8H_5NO_2Na$	$[M+Na]^+$	170.0213	170.0209	-2.05	No	-	Level 3
5-Hydroxytryptophol	HMDB0001855	$C_{10}H_{12}NO_2$	$[M+H]^+$	178.0861	178.0868	-0.87	No	-	Level 3
5-Methoxy-3-indoleaceate	No ID match	$C_{11}H_{12}NO_3$	$[M+H]^+$	206.0803	206.0817	-3.68	No	-	Level 3
5-Methylcytosine	HMDB0002894	$C_5H_8N_3O$	$[M+H]^+$	126.0661	126.0667	-2.83	Yes	9.39	Level 2
5-Nitrilonorvaline	No ID match	$C_5H_9N_2O_2$	$[M+H]^+$	129.0658	129.0664	-0.70	No	-	Level 3
5-Oxoproline	HMDB0000267	$C_5H_8NO_3$	$[M+H]^+$	130.0499	130.0504	-0.56	No	-	Level 3
6-hydroxypseudooxynicotine	No ID match	$C_{10}H_{18}N_3O_2$	$[M+NH_4]^+$	212.1384	212.1399	-4.30	No	-	Level 3
6,7-Dihydroxycoumarin	HMDB0030819	$C_9H_7O_4$	$[M+H]^+$	179.0337	179.0344	-0.87	No	-	Level 3
6,8-Dihydroxy-7-methoxy-2H-chromen-2-one	No ID match	$C_{10}H_9O_5$	$[M+H]^+$	209.0436	209.0450	-4.42	No	-	Level 3
Acetylcadaverine	HMDB0002284	$C_7H_{17}N_2O$	$[M+H]^+$	145.1336	145.1341	-0.63	No	-	Level 3
Acetylhistamine	HMDB0013253	$C_7H_{15}N_4O$	$[M+NH_4]^+$	171.1239	171.1246	-0.96	No	-	Level 3
Acetyltaurine	HMDB0240253	$C_4H_{13}N_2O_4S$	$[M+NH_4]^+$	185.0595	185.0596	-1.28	No	-	Level 3

Adenine	HMDB0000034	$C_5H_6N_5$	$[M+H]^+$	136.0618	136.0623	-3.10	No	-	Level 3
Agmatine	HMDB0001432	$C_5H_{15}N_4$	$[M+H]^+$	131.1291	131.1297	0.08	No	-	Level 3
Benzaldehyde	HMDB0006115	C_7H_7O	$[M+H]^+$	107.0489	107.0497	-3.75	No	-	Level 3
Benzoate	No ID match	$C_7H_7O_2$	$[M+H]^+$	123.044	123.0446	-0.20	No	-	Level 3
Benzoquinoneacetic acid	No ID match	$C_8H_7O_4$	$[M+H]^+$	167.0339	167.0344	-0.33	No	-	Level 3
Cytidine 5'-diphosphate	No ID match	$C_9H_{19}N_4O_{11}P_2$	$[M+NH_4]^+$	421.0502	421.0526	0.73	No	-	Level 3
CerP 42:2;O4	No ID match	$C_{42}H_{83}NO_8P$	$[M+H]^+$	760.5836	760.5856	3.68	No	-	Level 3
Choline	HMDB0000097	$C_5H_{14}NO$	$[M+H]^+$	104.1066	104.1075	-4.16	Yes	19.74	Level 2
Choline phosphate	HMDB0001565	$C_5H_{15}NO_4P$	$[M+H]^+$	184.0732	184.0734	-0.56	No	-	Level 3
3-Hexenyl Pyruvate	No ID match	$C_9H_{18}NO_3$	$[M+NH_4]^+$	188.1279	188.1287	-0.81	No	-	Level 3
Coumarin	HMDB0001218	$C_9H_7O_2$	$[M+H]^+$	147.044	147.0446	-0.50	No	-	Level 3
Creatinine	HMDB0000562	$C_4H_8N_3O$	$[M+H]^+$	114.0661	114.0667	-0.48	No	-	Level 3
Cyclohexanone	No ID match	$C_6H_{14}NO$	$[M+NH_4]^+$	116.107	116.1075	-0.33	No	-	Level 3
Cyclohexylamine	HMDB0031404	$C_6H_{14}N$	$[M+H]^+$	100.1116	100.1126	-4.90	No	-	Level 3
Cyclopentanone	HMDB0031407	$C_5H_{12}NO$	$[M+NH_4]^+$	102.091	102.0919	-3.36	No	-	Level 3
Cytosine	HMDB0000630	$C_4H_6N_3O$	$[M+H]^+$	112.0504	112.0511	-0.66	Yes	11.4	Level 2
Cytosine	HMDB0000630	$C_4H_5N_3ONa$	$[M+Na]^+$	134.0324	134.0325	-0.29	No	-	Level 3
D-Sedoheptulose 7-phosphate	HMDB0001068	$C_7H_{15}O_{10}PK$	$[M+K]^+$	329.0018	329.0034	2.49	No	-	Level 3
Epiguanine	No ID match	$C_6H_8N_5O$	$[M+H]^+$	166.0723	166.0729	-0.13	No	-	Level 3
Epinephrine-4-sulfate	No ID match	$C_9H_{14}NO_6S$	$[M+H]^+$	264.0538	264.0542	-4.31	No	-	Level 3
Epinephrine-4-sulfate	No ID match	$C_9H_{13}NO_6SNa$	$[M+Na]^+$	286.0359	286.0356	1.05	No	-	Level 3
Erythrose	No ID match	$C_4H_8O_4K$	$[M+K]^+$	159.0052	159.0054	0.39	No	-	Level 3
Ethyl 1,2,5,6-tetrahydro-1-methyl-3-pyridinecarboxylate	No ID match	$C_9H_{16}NO_2$	$[M+H]^+$	170.1174	170.1181	-0.42	No	-	Level 3
Epinephrine-4-sulfate	No ID match	$C_{10}H_{16}O_4Na$	$[M+Na]^+$	223.0948	223.0941	3.23	No	-	Level 3
Riboflavin 5'-phosphate	No ID match	$C_{17}H_{21}N_4O_9P$	$[M+Na]^+$	479.0956	479.0938	4.91	No	-	Level 3
Furfuranol	No ID match	$C_5H_7O_2$	$[M+H]^+$	99.0436	99.0446	-4.49	No	-	Level 3

g-Guanidinobutyrate	HMDB0003464	$C_5H_{12}N_3O_2$	$[M+H]^+$	146.0924	146.0930	0.42	No	-	Level 3
Guanine	HMDB0000132	$C_5H_6N_5O$	$[M+H]^+$	152.0566	152.0572	-0.14	Yes	10.95	Level 2
Guanine	HMDB0000132	$C_5H_5N_5ONa$	$[M+Na]^+$	174.0385	174.0386	-0.61	No	-	Level 3
Heptanoic acid	HMDB0000666	$C_7H_{15}O_2$	$[M+H]^+$	131.1067	131.1072	0.23	No	-	Level 3
Hercynine	HMDB0029422	$C_9H_{16}N_3O_2$	$[M+H]^+$	198.1235	198.1243	-1.20	No	-	Level 3
Hex2Cer 33:1;O5	No ID match	$C_{45}H_{86}NO_{16}$	$[M+H]^+$	896.5976	896.5987	-1.95	No	-	Level 3
HHL	No ID match	$C_{10}H_{17}NO_3Na$	$[M+Na]^+$	222.1108	222.1101	-2.77	No	-	Level 3
Hippurate	HMDB0000714	$C_9H_{10}NO_3$	$[M+H]^+$	180.0653	180.0661	-1.04	No	-	Level 3
Histidinol	HMDB0003431	$C_6H_{12}N_3O$	$[M+H]^+$	142.0975	142.0980	-0.27	No	-	Level 3
Histidinol	HMDB0003431	$C_6H_{15}N_4O$	$[M+NH_4]^+$	159.1239	159.1246	-1.37	No	-	Level 3
Homocystine	HMDB0000676	$C_8H_{16}N_2O_4S_2Na$	$[M+Na]^+$	291.0454	291.0444	1.12	No	-	Level 3
Homospermidine	No ID match	$C_8H_{22}N_3$	$[M+H]^+$	160.1807	160.1814	-0.86	No	-	Level 3
Isopelletierine	No ID match	$C_8H_{16}NO$	$[M+H]^+$	142.1227	142.1232	0.08	No	-	Level 3
L-Arginine	HMDB0000517	$C_6H_{15}N_4O_2$	$[M+H]^+$	175.1188	175.1195	-1.90	No	-	Level 3
L-Cysteate	HMDB0002757	$C_3H_{11}N_2O_5S$	$[M+NH_4]^+$	187.0388	187.0389	-0.46	No	-	Level 3
L-fucopyranose 1-phosphate	No ID match	$C_6H_{14}O_8P$	$[M+H]^+$	245.041	245.0426	-4.25	No	-	Level 3
L-Glutamine	HMDB0000641	$C_5H_{11}N_2O_3$	$[M+H]^+$	147.0764	147.0770	-0.38	No	-	Level 3
L-Norleucine	No ID match	$C_6H_{13}NO_2K$	$[M+K]^+$	170.0576	170.0576	-0.20	No	-	Level 3
L-Phenylalanine	HMDB0000159	$C_9H_{12}NO_2$	$[M+H]^+$	166.0862	166.0868	-0.22	No	-	Level 3
L-Proline	HMDB0000162	$C_5H_9NO_2K$	$[M+K]^+$	154.026	154.0265	-0.31	No	-	Level 3
L-Valine	HMDB0000883	$C_5H_{11}NO_2K$	$[M+K]^+$	156.0417	156.0421	-0.57	No	-	Level 3
Metanephrine	HMDB0004063	$C_{10}H_{15}NO_3Na$	$[M+Na]^+$	220.0952	220.0944	3.55	No	-	Level 3
Methionine sulfone	HMDB0062174	$C_4H_{13}N_2O_5S$	$[M+NH_4]^+$	201.0544	201.0545	-1.03	No	-	Level 3
N-(3-acetamidopropyl)pyrrolidin-2-one	No ID match	$C_9H_{20}N_3O_2$	$[M+NH_4]^+$	202.1548	202.1556	-2.83	No	-	Level 3
N-Acetyl-L-phenylalanine	HMDB0000512	$C_{11}H_{14}NO_3$	$[M+H]^+$	208.0959	208.0974	-4.22	No	-	Level 3

N-Acetylputrescine	HMDB0002064	$C_6H_{15}N_2O$	$[M+H]^+$	131.1179	131.1184	0.33	No	-	Level 3
N-Methyl-L-histidine	No ID match	$C_7H_{15}N_4O_2$	$[M+NH_4]^+$	187.1188	187.1195	-0.55	No	-	Level 3
N2-(1-Carboxyethyl)lysine	No ID match	$C_9H_{22}N_3O_4$	$[M+NH_4]^+$	236.1595	236.1610	-0.62	No	-	Level 3
NAE 10:1	No ID match	$C_{12}H_{24}NO_2$	$[M+H]^+$	214.1792	214.1807	-4.46	No	-	Level 3
NAE 10:2	No ID match	$C_{12}H_{22}NO_2$	$[M+H]^+$	212.1636	212.1651	-4.27	No	-	Level 3
NAT 11:3	No ID match	$C_{13}H_{21}NO_4SK$	$[M+K]^+$	326.0831	326.0823	3.12	No	-	Level 3
Nicotinamide	HMDB0001406	$C_6H_7N_2O$	$[M+H]^+$	123.0552	123.0558	-0.46	No	-	Level 3
Nicotinamide	HMDB0001406	$C_6H_{10}N_3O$	$[M+NH_4]^+$	140.0818	140.0824	-0.27	No	-	Level 3
Norpseudopelletierine	No ID match	$C_8H_{17}N_2O$	$[M+NH_4]^+$	157.1336	157.1341	-0.55	No	-	Level 3
O-Acetylcarnitine	HMDB0000201	$C_9H_{17}NO_4Na$	$[M+Na]^+$	226.1057	226.1050	3.44	No	-	Level 3
O-propenoyl-D-carnitine	No ID match	$C_{10}H_{17}NO_4Na$	$[M+Na]^+$	238.1048	238.1050	-0.61	No	-	Level 3
Phenylbutyric acid	No ID match	$C_{10}H_{12}O_2Na$	$[M+Na]^+$	187.073	187.0731	2.57	No	-	Level 3
Phenylglyoxal	No ID match	$C_8H_{10}NO_2$	$[M+NH_4]^+$	152.0706	152.0712	-0.57	No	-	Level 3
Phenylpyruvate	HMDB0000205	$C_9H_9O_3$	$[M+H]^+$	165.0546	165.0552	-0.51	No	-	Level 3
Propionylcarnitine	HMDB0000824	$C_{10}H_{19}NO_4Na$	$[M+Na]^+$	240.1203	240.1206	-1.74	No	-	Level 3
Pyridine	HMDB0000926	$C_5H_9N_2$	$[M+NH_4]^+$	97.0756	97.0766	-1.53	No	-	Level 3
Pyrrolidine	No ID match	C_4H_9NK	$[M+K]^+$	110.0362	110.0367	-0.97	No	-	Level 3
Ribose 5-phosphate	HMDB0001548	$C_5H_{12}O_8P$	$[M+H]^+$	231.0263	231.0270	2.50	No	-	Level 3
3-oxodecanoyl cysteamine	No ID match	$C_{12}H_{24}NO_2S$	$[M+H]^+$	246.1526	246.1528	-4.70	No	-	Level 3
Salicyluric acid	HMDB0000840	$C_9H_{10}NO_4$	$[M+H]^+$	196.0602	196.0610	-0.25	No	-	Level 3
Scopoletin	HMDB0034344	$C_{10}H_9O_4$	$[M+H]^+$	193.0494	193.0501	-0.75	No	-	Level 3
sedoheptulose 1,7-bisphosphate	No ID match	$C_7H_{16}O_{13}P_2Na$	$[M+Na]^+$	392.9947	392.9958	-2.98	No	-	Level 3
Spermidine	HMDB0001257	$C_7H_{20}N_3$	$[M+H]^+$	146.1651	146.1657	-0.02	No	-	Level 3
Spermine	HMDB0001256	$C_{10}H_{27}N_4$	$[M+H]^+$	203.2222	203.2236	-1.00	No	-	Level 3
ST 18:1;O	No ID match	$C_{18}H_{28}OK$	$[M+K]^+$	299.1785	299.1772	2.63	No	-	Level 3
ST 18:5;O5;T	No ID match	$C_{20}H_{25}NO_7SK$	$[M+K]^+$	462.1006	462.0983	-4.03	No	-	Level 3

ST 19:1;O	No ID match	$C_{19}H_{30}OK$	$[M+K]^+$	313.1938	313.1928	4.94	No	-	Level 3
ST 20:0;O	No ID match	$C_{20}H_{34}OK$	$[M+K]^+$	329.2255	329.2241	-4.99	No	-	Level 3
ST 20:5;O3;S	No ID match	$C_{20}H_{24}NO_6SK$	$[M+K]^+$	445.0938	445.0956	-4.83	No	-	Level 3
Tetrahydro-2,5-furan-diacetic acid	No ID match	$C_8H_{12}O_5K$	$[M+K]^+$	227.0322	227.0316	3.19	No	-	Level 3
Thiamin diphosphate	No ID match	$C_{12}H_{19}N_4O_7P_2S$	$[M+H]^+$	425.0459	425.0450	-4.30	No	-	Level 3
Threonic acid	HMDB0000943	$C_4H_8O_5K$	$[M+K]^+$	175	175.0003	-0.75	No	-	Level 3
Triethylamine	HMDB0032539	$C_6H_{16}N$	$[M+H]^+$	102.1273	102.1283	-3.33	No	-	Level 3
Tyramine	HMDB0000306	$C_8H_{15}N_2O$	$[M+NH_4]^+$	155.1178	155.1184	1.75	No	-	Level 3
Uridine monophosphate	HMDB0000288	$C_9H_{17}N_3O_9P$	$[M+NH_4]^+$	342.068	342.0702	4.60	No	-	Level 3

Table S3. List of annotated metabolite features detected in glioblastoma (GIN) cells from AP-MALDI-MSI datasets acquired under co-culture conditions with human astrocytes. The table includes all annotated metabolite features with corresponding adducts (*n* = 135 annotated adducts corresponding to *n* = 125 unique metabolites; multiple entries per metabolite reflect different detected adduct forms). Where available, annotations were further supported by LC–MS/MS analysis through retention time (RT) and/or fragmentation matching to reference standards.

Metabolite	HMDB Identifier	Formula	Adduct	Observed m/z	Theoretical m/z	Mass Error (ppm)	LC-MS confirmed	LC-MS RT (min)	Identification Level
1-(sn-glycero-3-phospho)-1D-myo-inositol	No ID match	$C_9H_{19}NaO_{11}P$	$[M+Na]^+$	357.0567	357.0557	2.74	No	-	Level 3
1,2,3-Heptanetriol	No ID match	$C_7H_{17}O_3$	$[M+H]^+$	149.1173	149.1178	0.53	No	-	Level 3
1,2,3-Heptanetriol	No ID match	$C_7H_{16}NaO_3$	$[M+Na]^+$	171.0989	171.0992	-1.55	No	-	Level 3
1,3,7-Octanetriol	No ID match	$C_8H_{18}NaO_3$	$[M+Na]^+$	185.1147	185.1148	-0.62	No	-	Level 3
1,9-Nonanedithiol	No ID match	$C_9H_{20}KS_2$	$[M+K]^+$	231.0627	231.0638	-4.76	No	-	Level 3
NAE 10:1	HMDB0000339	$C_{12}H_{24}NO_2$	$[M+H]^+$	214.1792	214.1807	-4.46	No	-	Level 3
1D-myo-Inositol 1,2-cyclic phosphate	No ID match	$C_6H_{12}O_8P$	$[M+H]^+$	243.0255	243.027	-3.84	No	-	Level 3
1H-Pyrazol-4-ylmethanol	No ID match	$C_4H_{10}N_3O$	$[M+NH_4]^+$	116.0818	116.0824	-0.33	No	-	Level 3
2-(4-Methylthiazol-5-yl)ethyl hexanoate	No ID match	$C_{12}H_{19}KNO_2S$	$[M+K]^+$	280.0771	280.0768	1.05	No	-	Level 3
2-Acetamidohexanedioic acid	No ID match	$C_8H_{14}NO_5$	$[M+H]^+$	204.0858	204.0872	-4.17	No	-	Level 3
2-Amino-4-methylpyrimidine	HMDB0034285	$C_5H_8N_3$	$[M+H]^+$	110.0712	110.0718	-0.66	No	-	Level 3
2-Amino-4-methylpyrimidine	HMDB0034285	$C_5H_{11}N_4$	$[M+NH_4]^+$	127.0977	127.0984	-0.96	No	-	Level 3
2-Aminoacetophenone	HMDB0033525	$C_8H_{10}NO$	$[M+NH_4]^+$	136.0758	136.0762	0.81	No	-	Level 3
2-Aminoethylphosphonic acid	HMDB0000129	$C_2H_9NO_3P$	$[M+H]^+$	126.0312	126.032	-2.03	No	-	Level 3
2-Aminohistamine	HMDB0033615	$C_5H_{13}N_4$	$[M+NH_4]^+$	129.1134	129.114	-0.56	No	-	Level 3
2-Chromanone	No ID match	$C_9H_{12}NO_2$	$[M+H]^+$	166.0862	166.0868	-0.33	No	-	Level 3
2-Ethyl-4,5-dimethylthiazole	No ID match	$C_7H_{15}N_2O$	$[M+NH_4]^+$	143.1179	143.1184	0.08	No	-	Level 3
2-Hydroxy-7-methyloctanedioic acid	No ID match	$C_9H_{16}KO_5$	$[M+K]^+$	243.0619	243.0629	-4.25	No	-	Level 3

2-Methoxy-3-methylpyrazine	No ID match	$C_6H_9N_2O$	$[M+H]^+$	125.071	125.0715	0.49	No	-		Level 3
2-methyl-1-methylthio-2-butene	No ID match	$C_6H_{12}KS$	$[M+K]^+$	155.0293	155.0291	1.11	No	-		Level 3
2-Oxo-5-pentyltetrahydro-3-furancarboxylic acid	No ID match	$C_{10}H_{16}NaO_4$	$[M+Na]^+$	223.0947	223.0941	2.78	No	-		Level 3
2-pyridone	HMDB0033357	$C_5H_9N_2O$	$[M+NH_4]^+$	113.0708	113.0715	-1.23	No	-		Level 3
2,6-di-tert-butyl-4-ethylphenol	No ID match	$C_{16}H_{26}KO$	$[M+K]^+$	273.1622	273.1615	2.48	No	-		Level 3
3-(4-Hydroxy-3-methoxyphenyl)-2-oxiranecarboxylic acid	No ID match	$C_{10}H_{10}NaO_5$	$[M+Na]^+$	233.0419	233.042	-0.62	No	-		Level 3
3-(4-Hydroxyphenyl)pyruvate	HMDB0000774	$C_9H_9O_4$	$[M+H]^+$	181.0494	181.0501	-0.75	No	-		Level 3
3-Amino-2-piperidinone	HMDB0034025	$C_5H_{14}N_3O$	$[M+H]^+$	132.1131	132.1137	-0.29	No	-		Level 3
3-Methoxy-4-hydroxymandelate	HMDB0000465	$C_9H_{10}NaO_5$	$[M+Na]^+$	221.0427	221.042	2.96	No	-		Level 3
3-Phenylpropanoic acid	HMDB0000763	$C_9H_{14}NO_2$	$[M+H]^+$	168.1018	168.1025	-0.63	No	-		Level 3
3-Sulfino-L-alanine	HMDB0004000	$C_3H_{11}N_2O_4S$	$[M+NH_4]^+$	171.044	171.044	3.48	No	-		Level 3
3,4-Dihydroxyphenylacetate	HMDB0000513	$C_8H_{12}NO_4$	$[M+NH_4]^+$	186.076	186.0766	-0.46	No	-		Level 3
4-Aminobenzoate	HMDB0001403	$C_7H_8NO_2$	$[M+H]^+$	138.0549	138.0555	-0.4	No	-		Level 3
4-Aminophenol	No ID match	$C_6H_{11}N_2O$	$[M+NH_4]^+$	127.0866	127.0871	0.09	No	-		Level 3
4-Ethyl-2-heptylthiazole	No ID match	$C_{12}H_{21}KNS$	$[M+K]^+$	250.1038	273.0919	4.69	No	-		Level 3
4-Hydroxybenzoic acid	HMDB0000500	$C_7H_7O_3$	$[M+H]^+$	139.0389	139.0395	-0.51	No	-		Level 3
5-Aminolevulinate	HMDB0000567	$C_8H_5NNaO_2$	$[M+Na]^+$	170.0208	170.0214	-3.54	No	-		Level 3
5-Hydroxytryptophol	HMDB0000769	$C_{10}H_{12}NO_2$	$[M+H]^+$	178.0861	178.0868	-0.87	No	-		Level 3
5-Methoxy-3-indoleaceate	HMDB0000879	$C_{11}H_{12}NO_3$	$[M+H]^+$	206.0804	206.0817	-3.74	No	-		Level 3
5-Methylcytosine	HMDB0000982	$C_5H_8N_3O$	$[M+H]^+$	126.0661	126.0667	-0.7	Yes		9.39	Level 2
g-Guanidinobutyrate	No ID match	$C_5H_{12}N_3O_2$	$[M+H]^+$	146.0924	146.093	-0.02	No	-		Level 3
5-Nitro-2-propoxyaniline	No ID match	$C_9H_{16}N_3O_3$	$[M+NH_4]^+$	214.1177	214.1192	-4.29	No	-		Level 3
5-Oxoproline	HMDB0000267	$C_5H_8NO_3$	$[M+H]^+$	130.0499	130.0504	0.23	No	-		Level 3
6-hydroxypseudooxynicotine	No ID match	$C_{10}H_{18}N_3O_2$	$[M+NH_4]^+$	212.1384	212.1399	-4.49	No	-		Level 3
6,7-Dihydroxycoumarin	No ID match	$C_9H_7O_4$	$[M+H]^+$	179.0338	179.0344	-0.48	No	-		Level 3
6,8-Dihydroxy-7-methoxy-2H-chromen-2-one	No ID match	$C_{10}H_9O_5$	$[M+H]^+$	209.0436	209.045	-4.07	No	-		Level 3
ACer 44:2;O4	No ID match	$C_{42}H_{83}NO_8P$	$[M+H]^+$	760.5836	760.5852	-2.1	No	-		Level 3

Acetylhistamine	HMDB0000881	$C_7H_{15}N_4O$	$[M+NH_4]^+$	171.124	171.1246	-0.22	No	-	Level 3
Acetyltaurine	HMDB0002297	$C_4H_{13}N_2O_4S$	$[M+NH_4]^+$	185.0595	185.0596	2.4	No	-	Level 3
Adenine	HMDB0000034	$C_5H_6N_5$	$[M+H]^+$	136.0619	136.0623	0.95	No	-	Level 3
Agmatine	HMDB0001515	$C_5H_{15}N_4$	$[M+H]^+$	131.1292	131.1297	0.59	No	-	Level 3
Benzaldehyde	HMDB0000300	C_7H_7O	$[M+H]^+$	107.0489	107.0497	-2.25	No	-	Level 3
Benzoic acid	HMDB0001873	$C_7H_7O_2$	$[M+H]^+$	123.0439	123.0446	-1.27	No	-	Level 3
Coumarin	HMDB0001505	$C_9H_7O_2$	$[M+H]^+$	147.0441	147.0446	0.3	No	-	Level 3
Creatinine	HMDB0000562	$C_4H_8N_3O$	$[M+H]^+$	114.0662	114.0667	0.1	No	-	Level 3
Cyclohexanone	No ID match	$C_6H_{14}NO$	$[M+NH_4]^+$	116.1069	116.1075	-0.78	No	-	Level 3
Cyclohexylamine	No ID match	$C_6H_{14}N$	$[M+H]^+$	100.1116	100.1126	-4.75	No	-	Level 3
Cyclopentanone	No ID match	$C_5H_{12}NO$	$[M+NH_4]^+$	102.091	102.0919	-3.33	No	-	Level 3
Cytidine Diphosphate	HMDB0001341	$C_9H_{19}N_4O_{11}P_2$	$[M+NH_4]^+$	421.0503	421.0526	-4.06	No	-	Level 3
Cytosine	HMDB0000630	$C_4H_6N_3O$	$[M+H]^+$	112.0505	112.0511	-0.34	Yes	11.4	Level 2
Cytosine	HMDB0000630	$C_4H_5N_3NaO$	$[M+Na]^+$	134.0324	134.0325	-0.61	No	-	Level 3
Deoxyribose 1-phosphate	HMDB0001408	$C_5H_{11}NaO_7P$	$[M+Na]^+$	237.0133	237.0135	-0.68	No	-	Level 3
Epiguanine	No ID match	$C_6H_8N_5O$	$[M+H]^+$	166.0723	166.0729	-0.22	No	-	Level 3
Epinephrine 4-sulfate	HMDB0001342	$C_9H_{14}NO_6S$	$[M+H]^+$	264.0539	264.0542	1	No	-	Level 3
Epinephrine 4-sulfate	HMDB0001342	$C_9H_{13}NNaO_6S$	$[M+Na]^+$	286.036	286.0356	1.47	No	-	Level 3
Erythrose	HMDB0000289	$C_4H_8KO_4$	$[M+K]^+$	159.0051	159.0054	-1.99	No	-	Level 3
Ethyl 1,2,5,6-tetrahydro-1-methyl-3-pyridinecarboxylate	No ID match	$C_9H_{16}NO_2$	$[M+H]^+$	170.1175	170.1181	-0.32	No	-	Level 3
FA 10:2;O2	No ID match	$C_{10}H_{16}KO_4$	$[M+K]^+$	239.0676	239.068	-1.74	No	-	Level 3
FA 11:3;O2	No ID match	$C_{11}H_{16}KO_4$	$[M+K]^+$	251.0668	251.068	-4.85	No	-	Level 3
Farnesylcysteine	HMDB0003012	$C_{18}H_{35}N_2O_2S$	$[M+NH_4]^+$	343.2418	343.2419	1.24	No	-	Level 3
Ferulic acid	HMDB0000425	$C_{10}H_{10}NaO_4$	$[M+Na]^+$	217.0479	217.0471	3.55	No	-	Level 3
Furfural	No ID match	$C_5H_3O_2$	$[M-H]^-$	95.014	95.0133	1.54	No	-	Level 3
Furfuranol	No ID match	$C_5H_7O_2$	$[M+H]^+$	99.0436	99.0446	-4.61	No	-	Level 3
Guanine	HMDB0000132	$C_5H_6N_5O$	$[M+H]^+$	152.0567	152.0572	0.09	Yes	10.95	Level 2
Guanine	HMDB0000132	$C_5H_5N_5NaO$	$[M+Na]^+$	174.0385	174.0386	-0.75	No	-	Level 3

Heptanoic acid	HMDB0000847	$C_7H_{15}O_2$	$[M+H]^+$	131.1067	131.1072	0.33	No	-	Level 3
Hercynine	HMDB0003055	$C_9H_{16}N_3O_2$	$[M+H]^+$	198.1235	198.1243	-1.03	No	-	Level 3
Hex2Cer 33:1;O5	No ID match	$C_{45}H_{86}NO_{16}$	$[M+H]^+$	896.5976	896.5947	3.89	No	-	Level 3
Hexylresorcinol	No ID match	$C_{12}H_{22}NO_2$	$[M+H]^+$	212.1636	212.1651	-4.27	No	-	Level 3
Hippurate	HMDB0000714	$C_9H_{10}NO_3$	$[M+H]^+$	180.0654	180.0661	-0.67	No	-	Level 3
Hippuryl-L-histidyl-L-leucine	No ID match	$C_{10}H_{17}NNaO_3$	$[M+Na]^+$	222.1108	222.1101	3.31	No	-	Level 3
histidinol	HMDB0001715	$C_6H_{15}N_4O$	$[M+NH_4]^+$	159.1239	159.1246	-0.86	No	-	Level 3
Homocystine	HMDB0000742	$C_8H_{16}N_2NaO_4S_2$	$[M+Na]^+$	291.0453	291.0444	3.2	No	-	Level 3
Homospermidine	HMDB0002039	$C_8H_{22}N_3$	$[M+H]^+$	160.1807	160.1814	-0.77	No	-	Level 3
L-Arginine	HMDB0000517	$C_6H_{15}N_4O_2$	$[M+H]^+$	175.1188	175.1195	-0.87	No	-	Level 3
L-Cysteate	HMDB0000053	$C_3H_{11}N_2O_5S$	$[M+NH_4]^+$	187.0387	187.0389	2.03	No	-	Level 3
L-fucopyranose 1-phosphate	No ID match	$C_6H_{14}O_8P$	$[M+H]^+$	245.0412	245.0426	-3.6	No	-	Level 3
L-Glutamine	HMDB0000641	$C_5H_{11}N_2O_3$	$[M+H]^+$	147.0764	147.077	-0.13	No	-	Level 3
L-Histidine	HMDB0000177	$C_6H_9N_3NaO_2$	$[M+Na]^+$	178.0579	178.0587	-4.48	No	-	Level 3
L-Norleucine	HMDB0001645	$C_6H_{13}KNO_2$	$[M+K]^+$	170.0576	170.0578	-1.09	No	-	Level 3
L-Proline	HMDB0000162	$C_5H_9NNaO_2$	$[M+Na]^+$	154.026	154.0265	-3.16	No	-	Level 3
L-Threonic acid	HMDB0000133	$C_4H_8KO_5$	$[M+K]^+$	175.0001	175.0003	-1.33	No	-	Level 3
L-Valine	HMDB0000883	$C_5H_{11}KNO_2$	$[M+K]^+$	156.0416	156.0421	-3.44	No	-	Level 3
Metanephrine	HMDB0000318	$C_{10}H_{15}NNaO_3$	$[M+Na]^+$	220.0952	220.0944	3.57	No	-	Level 3
Methcathinone	No ID match	$C_{10}H_{13}KNO$	$[M+K]^+$	202.0622	202.0629	-3.32	No	-	Level 3
Methionine sulfone	HMDB0001879	$C_4H_{13}N_2O_5S$	$[M+NH_4]^+$	201.0543	201.0545	1.64	No	-	Level 3
N-3-oxo-pentanoyl-L-Homoserine lactone	No ID match	$C_9H_{13}NNaO_4$	$[M+Na]^+$	222.0744	222.0737	3.25	No	-	Level 3
N-Acetyl-L-phenylalanine	HMDB0000765	$C_{11}H_{14}NO_3$	$[M+H]^+$	208.096	208.0974	-3.94	No	-	Level 3
N-Acetylputrescine	HMDB0002189	$C_6H_{15}N_2O$	$[M+H]^+$	131.1179	131.1184	0.08	No	-	Level 3
N-Methyl-L-histidine	HMDB0000001	$C_7H_{15}N_4O_2$	$[M+NH_4]^+$	187.1188	187.1195	-0.81	No	-	Level 3
N2-(1-Carboxyethyl)lysine	HMDB0002037	$C_9H_{22}N_3O_4$	$[M+NH_4]^+$	236.1596	236.161	-3.74	No	-	Level 3
NAT 11:3	No ID match	$C_{13}H_{21}KNO_4S$	$[M+K]^+$	326.083	326.0823	2.19	No	-	Level 3

Nicotinamide	HMDB0000140	$C_6H_7N_2O$	$[M+H]^+$	123.0552	123.0558	-0.72	No	-	Level 3
Nicotinamide	HMDB0000140	$C_6H_{10}N_3O$	$[M+NH_4]^+$	140.0818	140.0824	-0.27	No	-	Level 3
Norpseudopelletierine	No ID match	$C_8H_{17}N_2O$	$[M+NH_4]^+$	157.1335	157.1341	-0.25	No	-	Level 3
O-Acetylcarnitine	HMDB0000201	$C_9H_{17}NNaO_4$	$[M+Na]^+$	226.1056	226.105	2.75	No	-	Level 3
O-propenoyl-D-carnitine	No ID match	$C_{10}H_{17}NNaO_4$	$[M+Na]^+$	238.1047	238.105	-1.17	No	-	Level 3
Pentanal	No ID match	$C_5H_{14}NO$	$[M+H]^+$	104.1065	104.1075	-4.71	No	-	Level 3
Phenylbutyric acid	HMDB0000886	$C_{10}H_{12}NaO_2$	$[M+Na]^+$	187.073	187.073	0.27	No	-	Level 3
Phenylpyruvate	HMDB0000660	$C_9H_9O_3$	$[M+H]^+$	165.0546	165.0552	-0.13	No	-	Level 3
Propionylcarnitine	HMDB0000824	$C_{10}H_{19}NNaO_4$	$[M+Na]^+$	240.1203	240.1206	-1.37	No	-	Level 3
Pyridine	No ID match	$C_5H_9N_2$	$[M+NH_4]^+$	97.0756	97.0766	-4.37	No	-	Level 3
Pyrrole 2-carboxylate	HMDB0000719	$C_5H_9N_2O_2$	$[M+H]^+$	129.0658	129.0664	-0.42	No	-	Level 3
Rboflavin 5'-phosphate	HMDB0001490	$C_{17}H_{21}N_4NaO_9P$	$[M+Na]^+$	479.0956	479.0938	3.68	No	-	Level 3
Ribose 5-phosphate	HMDB0000283	$C_5H_{12}O_8P$	$[M+H]^+$	231.0264	231.027	-0.14	No	-	Level 3
S-3-oxodecanoyl cysteamine	No ID match	$C_{12}H_{24}NO_2S$	$[M+H]^+$	246.1527	246.1528	1.93	No	-	Level 3
Salicyluric acid	HMDB0000874	$C_9H_{10}NO_4$	$[M+H]^+$	196.0603	196.061	-0.69	No	-	Level 3
Scopoletin	HMDB0000877	$C_{10}H_9O_4$	$[M+H]^+$	193.0495	193.0501	-0.19	No	-	Level 3
Sedoheptulose 1,7-bisphosphate	HMDB0000246	$C_7H_{16}NaO_{13}P_2$	$[M+Na]^+$	392.9947	392.9958	-2.89	No	-	Level 3
Sedoheptulose 7-phosphate	HMDB0000247	$C_7H_{15}KO_{10}P$	$[M+K]^+$	329.0019	329.0034	-4.69	No	-	Level 3
Spermidine	HMDB0001257	$C_7H_{20}N_3$	$[M+H]^+$	146.1652	146.1657	0.18	No	-	Level 3
Spermine	HMDB0001258	$C_{10}H_{27}N_4$	$[M+H]^+$	203.2223	203.2236	-3.55	No	-	Level 3
ST 18:1;O	No ID match	$C_{18}H_{28}KO$	$[M+K]^+$	299.1785	299.1772	4.44	No	-	Level 3
ST 18:5;O5;T	No ID match	$C_{20}H_{25}KNO_7S$	$[M+K]^+$	462.1006	462.0983	4.91	No	-	Level 3
ST 19:1;O	No ID match	$C_{19}H_{30}KO$	$[M+K]^+$	313.1938	313.1928	3.12	No	-	Level 3
ST 20:0;O	No ID match	$C_{20}H_{34}KO$	$[M+K]^+$	329.2255	329.2241	4.19	No	-	Level 3
ST 20:5;O3;S	No ID match	$C_{20}H_{24}KNO_6S$	$[M+K]^+$	445.0939	445.0956	-3.8	No	-	Level 3
Tetrahydro-2,5-furan-diacetic acid	No ID match	$C_8H_{12}NaO_5$	$[M+Na]^+$	227.0322	227.0316	2.5	No	-	Level 3
Thiamin diphosphate	HMDB0001388	$C_{12}H_{19}N_4O_7P_2S$	$[M+H]^+$	425.0465	425.045	4.89	No	-	Level 3

Triethylamine	No ID match	$C_6H_{16}N$	$[M+H]^+$	102.1274	102.1283	-3.18	No	-	Level 3
Tryptophol	HMDB0002367	$C_5H_{15}NO_4P$	$[M+H]^+$	184.0731	184.0733	-1	No	-	Level 3
Tyramine	HMDB0000306	$C_8H_{15}N_2O$	$[M+NH_4]^+$	155.1178	155.1184	-0.57	No	-	Level 3

Table S4. List of annotated metabolite features detected in human astrocytes from AP-MALDI-MSI datasets acquired under co-culture conditions with glioblastoma (GIN) cells. The table includes all annotated metabolite features with corresponding adducts (*n* = 130 annotated adducts corresponding to *n* = 124 unique metabolites; multiple entries per metabolite reflect different detected adduct forms). Where available, annotations were further supported by LC–MS/MS analysis through retention time (RT) and/or fragmentation matching to reference standards.
